# Supplementary material for: Acute psychological impact of coronavirus disease 2019 outbreak among psychiatric professionals in China: a multicentre, cross-sectional, web-based study
Source: BMJ Open. 2021 May 11;11(5):e047828. doi: 10.1136/bmjopen-2020-047828 (PMC8117468; doi:10.1136/bmjopen-2020-047828)
Supplement: Author's manuscript [file bmjopen-2020-047828.draft_revisions.pdf]

BMJ Open

BMJ Open is committed to open peer review. As part of this commitment we make the peer review history of every article we publish publicly available.

When an article is published we post the peer reviewers' comments and the authors' responses online. We also post the versions of the paper that were used during peer review. These are the versions that the peer review comments apply to.

The versions of the paper that follow are the versions that were submitted during the peer review process. They are not the versions of record or the final published versions. They should not be cited or distributed as the published version of this manuscript.

BMJ Open is an open access journal and the full, final, typeset and author-corrected version of record of the manuscript is available on our site with no access controls, subscription charges or pay-per-view fees (http://bmjopen.bmj.com).

If you have any questions on BMJ Open's open peer review process please email info.bmjopen@bmj.com

Acute psychological impact of Coronavirus Disease 2019 outbreak among psychiatric professionals in China : A multicenter study

Journal:	*BMJ Open*
Manuscript ID	bmjopen-2020-047828
Article Type:	Original research
Date Submitted by the Author:	11-Dec-2020
Complete List of Authors:	Guo, Xin; Renmin Hospital of Wuhan University,<br>Ma, Simeng ; Renmin Hospital of Wuhan University<br>Yang, Jun ; Huazhong University of Science and Technology<br>Wang, Ying ; Renmin Hospital of Wuhan University<br>Hu, Shaohua ; Zhejiang University, Department of Psychiatry<br>Wang, Gaohua ; Renmin Hospital of Wuhan University<br>Liu, Zhong-Chun; Renmin Hospital of Wuhan University, Department of Psychiatry<br>Chen, Jianhua ; Shanghai Clinical Research Center for Mental Health, Shanghai Key Laboratory of Psychotic Disorders, Shanghai Mental Health Center, Shanghai Jiao Tong University School of Medicine<br>McCutcheon, Robert; King's College London<br>Arumuham, Atheeshaan; King's College London<br>Pillinger, Toby; King's College London
Keywords:	COVID-19, MENTAL HEALTH, PSYCHIATRY

SCHOLARONE™
Manuscripts

I, the Submitting Author has the right to grant and does grant on behalf of all authors of the Work (as defined in the below author licence), an exclusive licence and/or a non-exclusive licence for contributions from authors who are: i) UK Crown employees; ii) where BMJ has agreed a CC-BY licence shall apply, and/or iii) in accordance with the terms applicable for US Federal Government officers or employees acting as part of their official duties; on a worldwide, perpetual, irrevocable, royalty-free basis to BMJ Publishing Group Ltd ("BMJ") its licensees and where the relevant Journal is co-owned by BMJ to the co-owners of the Journal, to publish the Work in this journal and any other BMJ products and to exploit all rights, as set out in our licence.

The Submitting Author accepts and understands that any supply made under these terms is made by BMJ to the Submitting Author unless you are acting as an employee on behalf of your employer or a postgraduate student of an affiliated institution which is paying any applicable article publishing charge ("APC") for Open Access articles. Where the Submitting Author wishes to make the Work available on an Open Access basis (and intends to pay the relevant APC), the terms of reuse of such Open Access shall be governed by a Creative Commons licence – details of these licences and which Creative Commons licence will apply to this Work are set out in our licence referred to above.

Other than as permitted in any relevant BMJ Author's Self Archiving Policies, I confirm this Work has not been accepted for publication elsewhere, is not being considered for publication elsewhere and does not duplicate material already published. I confirm all authors consent to publication of this Work and authorise the granting of this licence.

For peer review only

Word count: 3290

Acute psychological impact of Coronavirus Disease 2019 outbreak among psychiatric professionals in China： A multicenter study

Xin Guo[1,2], Robert McCutcheon[2,3], Toby Pillinger[2,3], Atheeshaan Arumuham[2,3], Jianhua Chen[4], Simeng Ma[1], Jun Yang[5], Ying Wang[1], Shaohua Hu[6], Gaohua Wang[1], Zhongchun Liu[1]

[1]Department of Psychiatry, RenMin Hospital of Wuhan University, Wuhan, China

[2]Department of Psychosis Studies, Institute of Psychiatry, Psychology & Neuroscience, King's college of London, London, UK.

[3]Psychiatric Imaging Group, MRC London Institute of Medical Sciences, Hammersmith Hospital, London, UK

[4]Shanghai Clinical Research Center for Mental Health, Shanghai Key Laboratory of Psychotic Disorders, Shanghai Mental Health Center, Shanghai Jiao Tong University School of Medicine, Shanghai, China.

[5]Computer Science and Technology, Huazhong University of Science and Technology, Wuhan, China.

[6]Department of Psychiatry, First Affiliated Hospital, Zhejiang University School of Medicine, Hangzhou, China

For peer review only - http://bmjopen.bmj.com/site/about/guidelines.xhtml

Corresponding author:

Zhongchun Liu, PhD

Department of Psychiatry, RenMin Hospital of Wuhan University, Wuhan, China

238 Jiefang Road, Wuchang District, Wuhan, China

Email: zcliu6@whu.edu.cn

For peer review only

For peer review only - http://bmjopen.bmj.com/site/about/guidelines.xhtml

Abstract

Objectives: To assess the magnitude of mental health outcomes and associated factors among psychiatric professionals taking part in mental health services during COVID-19 in China.

Design, Setting and Participants: This cross-sectional, survey-based, region-stratified study collected demographic data and mental health measurements from 610 psychiatric professionals in 34 hospitals from 29 January and 7 February 2020, in China. Health care workers in hospitals equipped with fever clinics or wards for patients with COVID-19 were eligible.

Primary Outcome and Measures: The degree of symptoms of depression, anxiety, insomnia, and distress was assessed by the Chinese versions of PHQ-9, GAD-7, ISI, IES-R, respectively. Multivariable logistic regression analysis and Structural Equation Model was performed to identify factors associated with mental health outcomes.

Results: A total of 610 respondents were included, with a participation rate of 78.5%. 29.8% of them were work in Wuhan, and 22.5% were frontline mental health care workers. A considerable proportion of participants reported symptom of depression (461 [75.6%]), anxiety (282 [46.2%]), insomnia (336 [55.1%]) and mental stress (481 [78.9%]). Psychiatric professionals working at frontline, employed in Wuhan, treating and diagnosing infected patient, working with infected colleague, worrying about

For peer review only - http://bmjopen.bmj.com/site/about/guidelines.xhtml

infection, with a worse self-perceived physical health status, and those who obtaining information about COVID-19 from relatives reported higher prevalence of all measurements of mental health symptoms. Mental health service through psychological support materials and media, having enough personal protection and information about COVID-19 from TV were significantly associated with lower prevalence of mental health symptoms. The SEM analysis indicated that worry about infection could be the primary mediator affect the mental health of psychiatric professionals during the COVID-19 break.

Conclusions: These findings emphasize the importance of being prepared to protect the mental health of psychiatric professionals themselves through mental health interventions at times of widespread crisis.

For peer review only

For peer review only - http://bmjopen.bmj.com/site/about/guidelines.xhtml

Strengths and limitations of this study

- Provide one of the first comprehensive nationwide evaluation of the acute psychological impact on psychiatric professionals at the early break of COVID-19 in China.
- Provide a set of potential risk and protective factors related to mental health of psychiatric professionals during the epidemic of COVID-19.
- Emphasize the importance of being prepared to protect the mental health of psychiatric professionals themselves through mental health interventions at times of widespread crisis.
- This cross-sectional study does not record the mental health status of psychiatric professionals before the break or track the efficacy of psychological services among psychiatric professionals. A randomized prospective study could better determine correlation and causation.
- A larger sample size is needed to verify the results.

For peer review only - http://bmjopen.bmj.com/site/about/guidelines.xhtml

For peer review only

Introduction

The 2019 epidemic of novel coronavirus disease (COVID-19) that initiated in Wuhan (Hubei Province, China),[1] is now recognized by the World Health Organization (WHO) as a global public health threat. [2]. In addition to the resulting increased burden on physical healthcare services, there is increasing recognition of the impact that the pandemic may have upon public mental health and thereby mental health services. An effectively functioning mental health service required sufficient levels of staffing, and staff that are in good physical and mental health themselves.

The mental health of health-care workers in general has been affected during the immediate wake of the COVID-19 epidemic.[3] [4] Psychological distress among medical staff has been noted in the form of increasing symptoms of anxiety, depression, insomnia and posttraumatic stress symptoms [5] [6]. In addition, workers may also experience isolation from their families or community because of infection transmission risk and stigmatization. [7]

The specific impact of the pandemic on the mental health of psychiatric professionals is less well characterised. In the current study, a multicenter survey was performed to assess the psychological distress in psychiatric professionals in China one week after Wuhan city locked down. A brief questionnaire was designed and distributed to psychiatric professionals of several hospitals through WeChat group chat application,

For peer review only - http://bmjopen.bmj.com/site/about/guidelines.xhtml

including the psychiatric professionals serving in psychiatric hospitals and a psychiatric department of general hospital in Hubei province (including Wuhan) and other provinces, and also including psychiatric professionals working in fever clinics, infectious disease department and other departments. In addition, we investigated which factors are associated with risk of mental health symptoms among psychiatric professionals, and whether there was any evidence for any potential protective factors.

2. Methods

2.1 Online survey

A WeChat-based online survey was carried out among psychiatric professionals between 29 January and 7 February 2020, the second week after Wuhan city locked down. During the survey period, the cumulative number of confirmed and suspected COVID-19 cases was climbing exponentially (see Fig. 1).

The survey was conducted with the WeChat-based survey program ‘Questionnaire Star’. The target population was psychiatric professionals working in hospitals that established fever clinics or wards for patients with COVID-19. Each WeChat account of participant was authenticated by his/her ID card in China. The Quick Response (QR) code directing to the online survey was distributed in psychiatric professionals’ working groups of several provinces. Filling the questionnaires outside of working

For peer review only - http://bmjopen.bmj.com/site/about/guidelines.xhtml

hours was advised. This study was reviewed and approved by the Clinical Research Ethics Committee of Renmin Hospital of Wuhan University (WDRY2020-K004). All subjects provided informed consent electronically prior to registration. Respondents could terminate the survey at any time or during any question if they wished to.

2.2 Patient and Public Involvement

No patient involved

2.3 Questionnaire

The questionnaire consists of seven parts: basic demographic data, mental health assessment, risks of exposure to COVID-19, source of information about COVID-19, mental healthcare services (MHS) accessed, worry about infection and self-perceived physical health that before the COVID-19 outbreak. The questionnaire required 15 minutes to complete.

2.3.1 Demographic data

Basic demographic data include gender, age, marital status (unmarried/other, married), educational level (high school or lower, undergraduate, postgraduate or higher), location, job type (doctor or nurse), job title (junior, intermediate, or senior), and hospital type (tertiary, secondary or other).

2.3.2 Mental health assessment

For peer review only - http://bmjopen.bmj.com/site/about/guidelines.xhtml

We used four well validated scales to assess the mental health status of psychiatric professionals. The 9-item Patient Health Questionnaire (PHQ-9), the 7-item Generalized Anxiety Disorder (GAD-7), the 7-item Insomnia Severity Index (ISI) and the 22-item Impact of Event Scale-Revised (IESR) were used to evaluate depression, anxiety, insomnia and stress, respectively. The PHQ-9 is a self-report measure used to assess the severity of depression and has good reliability and validity in Chinese population (Cronbach's α=0.89), [8] with the total scores ranged from 0-27, and a s score of 10 or greater defined as a high probability of meeting diagnostic criteria for depression. [9]The GAD-7 is a self-rated scale to evaluate the severity of anxiety and has good reliability and validity in Chinese population (Cronbach's α=0.89), [10] the total score of GAD-7 ranged from 0 to 21, and a score of greater than 10 was identified as a high probability of meeting diagnostic criteria for anxiety. [11] The ISI is a measure of insomnia severity that has been shown to be valid and reliable, and a total score of greater than 10 was defined as a high probability of meeting diagnostic criteria for insomnia. [12] Post traumatic symptoms response to a specific stressful life event were accessed using the IES-R, which also has extensive reliability and validity (Cronbach's α for 3 subscales were 0.89 (Intrusion), 0.85 (Avoidance), and 0.83 (Hyperarousal), respectively), [13] a score of greater than 34 was identified as mental stress. [14]

2.3.3 Risk of exposure to COVID-19

Exposure to COVID-19 was determined with the following questions asked to

For peer review only - http://bmjopen.bmj.com/site/about/guidelines.xhtml

psychiatric professionals: Have you been diagnosed with COVID-19? Do you manage patients diagnosed with COVID-19? Has your family been diagnosed with COVID-19? Have your friends been diagnosed? Have your neighbors (people living in the same community who may or may not know each other) been diagnosed? Participants were then asked whether there was anyone living with them with suspected symptoms. In terms of personal protection, participants were asked whether they had enough personal protection equipment (PPE) and enough training for personal protection. The answer to each question was yes or no.

2.3.4 Accessed mental health services (MHS)

The following question was used to determine which psychological services the subject had accessed. Have you ever received the following services: psychological support materials (leaflets, brochures and books provided by mental health workers and distributed to staff in the hospital), psychological resources available through media (psychological assistance methods and techniques provided by psychologists through social media, and online counseling or psychotherapy (including individual or group counselling, or structural psychotherapy)?

2.3.5 Source of information about COVID-19

The following question was used to determine from which source the subject had accessed for information of COVID-19. Which way do you used to get information about COVID-19: relatives (information through direct communication with family,

For peer review only - http://bmjopen.bmj.com/site/about/guidelines.xhtml

neighbors, colleagues, friends or strangers), social media (information from unofficial accounts in WeChat Moments, Weibo, TikTok, and other digital platform), TV (information from national and local official television channels), and other (other information sources of COVID-19) and daily time (hours) spending on information of COVID-19. Then, participants were asked how helpful the information they received were? Answer options, including considerably helpful, medium helpful, slightly helpful, not helpful at all.

2.3.6 Self-perceived physical health

Physical health status was determined by asking participants to compare their health status to normal people before the outbreak of COVID-19: How do you perceive your health status compared to normal people before the outbreak? Answer option included strong, normal and weak.

2.3.7 Worry about infection

Worry about infection was determined by asking participants if they worry about their vulnerability to infection of COVID-19 in workplace or at home, answer options were yes or no.

2.4 Statistical analysis

All statistical analyses were carried out in the statistical programming language R

For peer review only - http://bmjopen.bmj.com/site/about/guidelines.xhtml

(version-3.6.0). [15]

A bivariate analysis was performed firstly by using Chi-square test to determine the effect of six components (demographic data, risks of exposure to COVID-19, source of information about COVID-19, MHS accessed, worry about infection and self-perceived physical health) on prevalence of four mental health symptoms (depression, anxiety, insomnia and mental stress, assessed by using PHQ-9, GAD-7, ISI, and IES-R separately) among psychiatric professionals.

Stepwise logistic regression analysis according to Akaike information criterion (AIC) was then performed to examine the effects of the six components on four mental health assessment (PHQ-9, GAD-7, ISI, and IES-R scores) separately. The significance of association between the potential risk factors and outcomes were checked by analysis of variance (ANOVA) and prediction accuracy of models were counted by AUC (area under ROC curve).

Finally, a structural equation model (SEM) was constructed via R package lavaan 0.6-7 [16] to explore the relationship between mental health assessment (PHQ-9. GAD-7, ISI and IES-R) and the six components, namely demographic data, risks of exposure to COVID-19, source of information about COVID-19, MHS accessed, worry about infection and self-perceived physical health. The estimation method used weighted least squares with mean and variance adjustment test statistics.[17] We used a Monte

For peer review only

Carlo method with 1000 guided resamplings to construct a confidence interval for the estimation effect.[18] In SEM, several criteria, such as ratio of value of chi-square test of model fit and degree of freedom (x^2 / df) <3, root mean square error of approximation (RMSEA) values<0.08 and goodness-to-fit index (GFI) values>0.90, indicate acceptable models (Hu and Bentler, 1998).

P values < 0.05 indicated that a difference was statistically significant.

3. Results

Initially, 834 completed questionnaires were received, with a response rate of 78.5%. The duration (seconds) to complete the questionnaire was recorded by Questionnaire star in WeChat. We excluded 119 questionnaires with a completion duration of less than 5 minutes (300 seconds) and more than 20 minutes (1,200 seconds), to ensure that all questions had been well understood and completed consecutively by respondents. We further excluded 105 questionnaires from provinces that had recruited less than 20 participants. In total, 224 questionnaires were excluded, leaving 610 questionnaires for analysis. Respondents completing the included questionnaires and excluded questionnaires were comparable in age and sex.

3.1 Demographic characteristics

In total, 610 psychiatric professionals, including 164 (26.9%) doctors and 446 (73.1%)

For peer review only - http://bmjopen.bmj.com/site/about/guidelines.xhtml

For peer review only

nurses were included into analysis, 29.8% of these participants were employed in Wuhan, and 53.3% were employed in Hubei provinces other than Wuhan. 22.5% were frontline mental health care workers. 53.3% of participants were working in a tertiary hospital. The participants tended to be female (76.1%), be aged 26-40 years (63.1%), be married (68.2%), have an educational level of undergraduate (91.3%), and have a junior job title (62.1%), as shown in Table 1.

3.2 Mental health assessment

According to standard questionnaire cut-offs of the PHQ-9, GAD-7, ISI, IES-R scores, 461 (75.6%) of individuals met criteria for depression, 282 (46.2%) for anxiety, 336 (55.1%) for insomnia, and 481 (78.9%) for high levels of mental stress.

3.3 Assessed mental health services (MHS)

36.3% of participants had received psychological support materials, 50.4% had obtained psychological resources available through media, and 17.5% had participated in online psychological counseling/psychotherapy, as shown in Table 1.

3.3 Risk factors & protective factors of psychological impact of COVID-19 on psychiatric professionals

Bivariate Analysis

We found that psychiatric professionals took part in frontline work presented higher

For peer review only - http://bmjopen.bmj.com/site/about/guidelines.xhtml

prevalence of all four mental health problem (89.9% vs 71.5%, P<0.001 for depression; 66.4% vs 40.4%, P<0.001 for anxiety; 67.9% vs 51.4%, P<0.001 for insomnia; 69.3% vs 52.0%, P<0.001 for mental stress) compared with those who worked at secondary line. Treating and diagnosing infected patient (95% vs 72.6%, P<0.001 for depression; 75% vs 41.9%, P<0.001 for anxiety; 75% vs 52.1%, P<0.001 for insomnia; 82.5% vs 51.9%, P<0.001 for mental stress), working with infected colleague (87.9% vs 71.9%, P<0.001 for depression; 67.9% vs 39.8%, P<0.001 for anxiety; 66% vs 52%, P<0.001 for insomnia; 72.1% vs 51.1%, P<0.001 for mental stress), employed in Wuhan (Wuhan vs Hubei except Wuhan vs Other provinces except Hubei: 89% vs 72.6% vs 61.2%, P<0.001 for depression; 65.4% vs 39.1% vs 35%, P<0.001 for anxiety; 70% vs 49% vs 48.5%, P<0.001 for insomnia; 72.5% vs 49.5% vs 46.6, P<0.001 for mental stress), worse self-perceived physical health (strong vs normal vs weak: 60.7% vs 91.5% vs 100%, P<0.001 for depression; 26.4% vs 67.1% vs 88.9%, P<0.001 for anxiety; 34.6% vs 76.7% vs 100%, P<0.001 for insomnia; 39.6% vs 73.1% vs 88.9, P<0.001 for mental stress), worrying about infection (83.5% vs 46.2%, P<0.001 for depression; 54.2% vs 16.9%, P<0.001 for anxiety; 60.6% vs 34.6%, P<0.001 for insomnia; 63.5% vs 27.7%, P<0.001 for mental stress) and obtaining information about COVID-19 from relatives (79.4% vs 71.1%, P=0.02 for depression; 50.9% vs 40.8%, P=0.02 for anxiety; 59% vs 50%, P=0.03 for insomnia; 64.4% vs 46.1%, P<0.001 for mental stress) are also significantly associated with higher prevalence of all four mental health symptoms. Self-infection (100% vs 45.8%, p=0.049) and living with infected neighbor (65.4% vs 39.1, p=0.002) were significantly associated with higher prevalence of anxiety.

For peer review only - http://bmjopen.bmj.com/site/about/guidelines.xhtml

Mental health service through psychological support materials (70.7% vs 79.9%, P=0.01 for depression; 38.7% vs 52.9%, P<0.001 for anxiety; 49% vs 61%, P=0.004 for insomnia; 51.6% vs 59.8%, P=0.05 for mental stress), mental health service through media (71.5% vs 81.3%, P=0.007 for depression; 40.8% vs 54%, P=0.002 for anxiety), and obtaining information about COVID-19 from TV (70.3% vs 81.9%, P=0.001 for depression; 41.7% vs 51.6%, P=0.02 for anxiety; 51% vs 60%, P=0.02 for insomnia; 50.5% vs 62.5%, P=0.004 for mental stress) were significantly associated with lower prevalence of mental health symptoms, as shown in Table 2.

Logistic Regression

To explore the potential risk and protective factors of mental health of psychiatric professionals, stepwise logistic regression analyses on PHQ-9, GAD-7, ISI and IES-R scores separately was performed. After controlling for potential confounding variables, checking the significance of effect of each variable using ANOVA, we identified risk and protective factors as follows (Table 3):

PHQ-9

Treating and diagnosing infected patient (odds ratio [OR], 2.77; 95% CI, 1.05-7.27; P = .039), worry about infection (OR, 0.33; 95% CI, 0.21-0.52; p<0.001), having inadequate PPE (OR, 2.43; 95% CI, 1.32-4.47; p=0.005), worse self-perceived physical health (OR, 2.76; 95% CI, 1.73-4.41; p<0.001) were significantly associated with higher PHQ-9 score among psychiatric professionals. Obtaining information about

For peer review only - http://bmjopen.bmj.com/site/about/guidelines.xhtml

COVID-19 from relatives (OR, 1.64; 95% CI, 1.03-2.61; p=0.036) was also significantly associated with increased PHQ-9 score, while obtaining information from TV (OR, 0.57; 95% CI, 0.36-0.91; p=0.02) was significantly associated with lower PHQ-9 score.

GAD-7

Doctors were found higher GAD-7 score (OR, 0.55; 95% CI, 0.34-0.91; p=0.019) compared with nurses. Self-infection seems a primary risk factor to anxiety (OR, 2530278.72; 95% CI, 0-Inf; p<0.001), and all self-infection psychiatric professionals met criteria of sever anxiety. Treating and diagnosing infected patient (OR, 2.36; 95% CI, 1.27-4.39; p=0.007), working with infected colleague (OR, 2.17; 95% CI, 1.31-3.61; p=0.003), living with infected neighbor (OR, 2.13; 95% CI, 1.14-3.96; p=0.018), having inadequate PPE (OR, 1.7; 95% CI, 1.09-2.65; p=0.02), worry about infection (OR, 0.25; 95% CI, 0.14-0.44; p<0.001) and obtaining information from other methods (OR, 2.36; 95% CI, 1.29-4.32; p=0.005) were associated with increased GAD-7 score. MHS through psychological support materials (OR, 0.66; 95% CI, 0.43-0.99; p<0.001) and those who think information about COVID-19 was helpful (OR, 1.17; 95% CI, 1.01-1.36; p=0.35) showed lower GAD-7 score.

ISI

Treating and diagnosing infected patient (OR, 1.91; 95% CI, 1.06-3.45; p=0.032), worse physical health status (OR, 5.69; 95% CI, 3.93-8.25; p<0.001) and those

For peer review only - http://bmjopen.bmj.com/site/about/guidelines.xhtml

worrying about infection (OR, 0.57; 95% CI, 0.36-0.91; p=0.017) were significantly associated with higher ISI score.

IES-R

Treating and diagnosing infected patient (OR, 2.91; 95% CI, 1.51-5.58; p=0.001), worse self-perceived physical health (OR, 3.26; 95% CI, 2.27-4.68; p<0.001) and worry about infection (OR, 0.34; 95% CI, 0.21-0.54; p<0.001) were significantly associated with higher IES-R score among psychiatric professionals. Psychiatric professionals obtaining information from relatives showed significantly higher IES-R score (OR, 2.13; 95% CI, 1.44-3.15; p<0.001), while those who obtaining information from TV (OR, 0.52; 95% CI, 0.35-0.77; p=0.001), and those think information about COVID-19 was helpful (OR, 1.15; 95% CI, 1.1-1.31; p=0.043) showed lower IES-R score.

Structural Equation Modelling

Finally, we established an SEM of the association between the seven areas described in methods. The chi-square test of model fit yielded a value of 729.6, with degrees of freedom = 253, P value= 0.000, x^2 / df = 2.88, RMSEA = 0.056, GFI = 0.909, and TLI = 0.973, indicating a good fit. The results showed that gender, the risk factors of exposure, source of information about COVID-19 and physical health perception affected mental health through worry about infection, indicated that worry about infection could be the primary mediator affect the mental health of psychiatric

For peer review only - http://bmjopen.bmj.com/site/about/guidelines.xhtml

professionals during the COVID-19 break. And mental health services alleviated mental health symptoms through decreasing the worry about infection. The results are shown in Figure 2.

4. Discussion

For peer review only

The present cross-section study firstly investigated the psychological impact of COVID-19 among psychiatric professionals in China during the early break of epidemic. The overall prevalence of depression, anxiety, insomnia and stress in psychiatric in the second week after Wuhan city locked down were 75.6%, 46.2%, 55.1%, and 78.9%, respectively. In comparison of recent studies of general health care workers [6, 19, 20], the psychological impact of COVID-19 outbreak seem to be more severe among psychiatric professionals compared with general health care workers (the overall prevalence of depression, anxiety, insomnia and stress was estimated at 13.9%-46.9%, 16.1%-41.1%, 19.7% and 9.8%-69.1%, respectively). Firstly, most of participants in the present study were from Wuhan and other cities in Hubei province, according to these recent studies, the prevalence of depression, anxiety and stress among health care workers working in Hubei province (including Wuhan) were estimated at 82.9%-88.7%, 66.9%-80.4%, and 87.6%-92.9%, respectively. Secondly, patients with mental health disorders were believed more susceptible to infections for several reasons, including cognitive impairment, little awareness of risk, and diminished efforts regarding personal protection in patients, also the confined conditions in psychiatric hospital

For peer review only - http://bmjopen.bmj.com/site/about/guidelines.xhtml

and psychiatric department in general hospital. [21] Our research also proved this, risk of exposure to COVID-19, inadequate personal protection, worrying about being infected and self-perceived physical health were significantly associated with higher risk of mental health symptoms among psychiatric professionals. Thirdly, the psychological impact of COVID-19 in different epidemic stages were different, [19, 20] our study was performed in the early wake of break and finished by the end of the second week after Wuhan city locked down, the rapid rising number of identified & suspected cases and unprecedented scale of city locked down caused public panic and psychological stress. [3] This could exacerbate the mental health problems among health care workers including psychiatric professionals.

The study firstly explored the psychological effect of different source of information about COVID-19 on mental health among psychiatric professionals. At the early stage of break, the unpredictable future of this epidemic has been worsened by myths and misinformation, often driven by erroneous news reports from unofficial information source and public's misunderstanding of health messages, thus causing worry and increasing the risk of mental health problems in population. [3] The WHO uses the term "infodemic" to describe the impossible amounts of misinformation and disinformation in worldwide, and the doctors and scientists have long been called on to share their wisdom in the official media. [22] [23] Our study proved that information from social media, direct communication with relatives, and other unofficial information sources increased risk of mental health symptoms among

For peer review only - http://bmjopen.bmj.com/site/about/guidelines.xhtml

psychiatric professionals. The information of COVID-19 from official source such as TV seems a protective factor for mental health among psychiatric professionals. Indicating that trustable sources of reliable and accurate information are not just helpful for epidemic control, but also good to mental health.

To efficiently cope with psychological impact of the COVID-19 outbreak, the Chinese Government has implemented rapid and comprehensive psychological crisis intervention, including publishing psychological support materials, such as leaflets and books, establishing psychological intervention teams in site [4] and mental health service online through social media platform, and also telephone guidance to help deal with mental health problems.[24] Our study found that psychiatric professionals had benefited from availability of mental health care from public mental health services (psychological materials and psychological help from media) and personalized mental health services (online counseling and psychotherapy).

Our study also has some limitations. First, the study is cross-sectional and does not record the mental health status before the break or track the efficacy of psychological services among psychiatric professionals. A randomized prospective study could better determine correlation and causation. Second, a larger sample size is needed to verify the results.

In summary, the results demonstrate that a strikingly large portion of psychiatric

For peer review only - http://bmjopen.bmj.com/site/about/guidelines.xhtml

professionals in China are suffering from mental health disturbances during the early break of COVID-19. Their mental health was primarily affected by worry about being infected, being female, risks of exposure to COVID-19, physical health and unreliable information source of COVID-19 may exacerbate it and they would benefit from greater availability of mental health care from public and personalized mental health services. Among the steps needed to better prepare for future infectious disease outbreaks would be a greater investment in the infectious diseases' prevention and control in psychiatric hospitals and psychiatric department in general hospitals, and in the mental health services among psychiatric professionals' themselves to protect and care for future psychiatric professionals who find themselves unexpectedly on the dangerous front lines of disease response.

Acknowledgments

Author Contribution: Dr Guo, Dr McCutcheon and Prof Liu had full access to all the data in the study and takes responsibility for the integrity of the data and the accuracy of the data analysis.

Design and conduct of the study: Dr Guo, Dr McCutcheon, Prof Liu. Collection, management, analysis, and interpretation of the data: All authors.

Preparation, review, or approval of the manuscript: Dr Guo, Dr McCutcheon, Dr Chen, Prof Liu and Prof Wang.

Decision to submit the manuscript for publication: Prof Liu and Prof Wang.

For peer review only

Funding/Support: XG’s work is funded by the National Key R&D Program of China (no. 2018YFC1314600), and fellowship grants from Chinese Scholarship Council. ZCL’s work is funded by the National Key R&D Program of China (no. 2018YFC1314600). RM’s research is funded by the Wellcome Trust (no. 200102/Z/15/Z) and a NIHR clinical lectureship.

Declaration of interest:

The authors declare that they have no conflict of interest.

For peer review only

References

1 Chan JF, Yuan S, Kok KH, et al. A familial cluster of pneumonia associated with the 2019 novel coronavirus indicating person-to-person transmission: a study of a family cluster. *Lancet*

For peer review only - http://bmjopen.bmj.com/site/about/guidelines.xhtml

2020;395(1474-547X (Electronic)):514-23.
2 WHO. Statement on the second meeting of the International Health Regulations (2005) Emergency Committee regarding the outbreak of novel coronavirus (2019-nCoV). https://www.who.int/news-room/detail/30-01-2020-statement-on-thesecond-meeting-of-the-international-health-regulations-(2005)-emergency-committee-regarding-the-outbreak-of-novel-coronavirus-(2019-ncov) (assessed Feb 15, 2020). 2020
3 Bao Y, Sun Y, Meng S, et al. 2019-nCoV epidemic: address mental health care to empower society. *Lancet* 2020;395(1474-547X (Electronic)):e37-e38.
4 Kang L, Li Y, Hu S, et al. The mental health of medical workers in Wuhan, China dealing with the 2019 novel coronavirus. *Lancet Psychiatry* 2020(2215-0374 (Electronic)) doi: https://doi.org/10.1016/s2215-0366(20)30047-x
5 da Silva FCT, Neto MLR. Psychiatric symptomatology associated with depression, anxiety, distress, and insomnia in health professionals working in patients affected by COVID-19: A systematic review with meta-analysis. *Prog Neuropsychopharmacol Biol Psychiatry* 2020(1878-4216 (Electronic)) doi: 10.1016/j.pnpbp.2020.110057
6 Lin K, Yang BX, Luo D, et al. The Mental Health Effects of COVID-19 on Health Care Providers in China. *American Journal of Psyhciatry* 2020(1535-7228 (Electronic))
7 Van Bortel T, Basnayake A, Wurie F, et al. Psychosocial effects of an Ebola outbreak at individual, community and international levels. *Bull World Health Organ* 2016;94(1564-0604 (Electronic)):210-14.
8 Chen S, Fang Y, Chiu H, et al. Validation of the nine-item Patient Health Questionnaire to screen for major depression in a Chinese primary care population. *Asia Pac Psychiatry* 2013;5(1758-5872 (Electronic)):61-68.
9 Manea L, Gilbody S, McMillan D. Optimal cut-off score for diagnosing depression with the Patient Health Questionnaire (PHQ-9): a meta-analysis. *Canadian Medical Association Journal* 2012;184(1488-2329 (Electronic)):e191-e96.
10 Tong X, An D, McGonigal A, et al. Validation of the Generalized Anxiety Disorder-7 (GAD-7) among Chinese people with epilepsy. *Epilepsy Res* 2015(1872-6844 (Electronic))
11 Spitzer R, Kroenke K, Williams J, et al. A brief measure for assessing generalized anxiety disorder: the GAD-7. *Arch Intern Med* 2006;166(0003-9926 (Print)):1092-97.
12 Morin C, Belleville G, Bélanger L, et al. The Insomnia Severity Index: psychometric indicators to detect insomnia cases and evaluate treatment response. *Sleep* 2011;34(1550-9109 (Electronic)):601-08.
13 Wu K, Chan K. The development of the Chinese version of Impact of Event Scale--Revised (CIES-R). *Soc Psychiatry Psychiatr Epidemiol* 2003;38(0933-7954 (Print)):94-98.
14 Morina N, Ehring T, Priebe S. Diagnostic utility of the impact of event scale-revised in two samples of survivors of war. *PLoS One* 2013;31(1932-6203 (Electronic)):e83916.
15 Team RC. R: A Language and Environment for Statistical Computing. 2013
16 Rosseel Y. Lavaan: A R package for structural equation modeling. *Journal of Statistical Software* 2012;48(2):1-36.
17 DiStefano C, Morgan GB. A Comparison of Diagonal Weighted Least Squares Robust Estimation Techniques for Ordinal Data. *Structural Equation Modeling: A Multidisciplinary Journal* 2014;21(3):425-38. doi: 10.1080/10705511.2014.915373
18 Bauer DJ, Preacher KJ, Gil KM. Conceptualizing and testing random indirect effects and moderated

For peer review only - http://bmjopen.bmj.com/site/about/guidelines.xhtml

mediation in multilevel models: new procedures and recommendations. *Psychol Methods* 2006;11(2):142-63. doi: 10.1037/1082-989x.11.2.142 [published Online First: 2006/06/21]

19 Wang Y, Ma S, Yang C, et al. Acute psychological effects of Coronavirus Disease 2019 outbreak among healthcare workers in China: a cross-sectional study. *Transl Psychiatry* 2020;10(2158-3188 (Electronic)):348.

20 Wang L, Zhang M, Liu G, et al. Psychological impact of coronavirus disease (2019) (COVID-19) epidemic on medical staff in different posts in China: A multicenter study. *Journal of Psychiatric Research* 2020;129(1879-1379 (Electronic)):198-205.

21 Yao H, Chen JH, Xu YF. Patients with mental health disorders in the COVID-19 epidemic. *Lancet Psychiatry* 2020;7(2215-0374 (Electronic)):e21.

22 Cinelli M, Quattrociocchi W, Galeazzi A, et al. The COVID-19 social media infodemic. *Sci Rep* 2020(2045-2322 (Electronic)) doi: 10.1038/s41598-020-73510-5

23 Diseases TLI. The COVID-19 infodemic. *Lancet* 2020;20(8):875.

24 Liu S, Yang L, Zhang C, et al. Online mental health services in China during the COVID-19 outbreak. *Lancet Psychiatry* 2020(2215-0374 (Electronic)) doi: 10.1016/S2215-0366(20)30077-8

For peer review only

For peer review only - http://bmjopen.bmj.com/site/about/guidelines.xhtml

Figure & Table Legend

Figure 1. Timeline of COVID-19 and survey data. Wuhan city was locked down since 23 Jan 2020, and the online survey was conducted 1 week after that. The cumulative number seemed to have entered the rapid rise phase.

------------------------------ Figure 1----------------------------

Figure 2. Direct and indirect effects in SEM. The solid line represents a significant relationship between the two, while the dotted line represents the relationship is not significant.

------------------------------ Figure 2---------------------------

For peer review only - http://bmjopen.bmj.com/site/about/guidelines.xhtml
For peer review only

Table 1. Demographic characteristics and accessed mental health services

Variables		Number	Percentage (%)
Total		610	100
Gender	Male	464	76.1
	Female	146	23.9
Age (years)	18-25	92	15.1
	26-30	198	32.5
	31-40	187	30.7
	>40	133	21.8
Marital status	Unmarried/Other	194	31.8
	Married	416	68.2
Education level	High school or less	18	3.0
	Undergraduate	557	91.3
	Graduate or higher	35	5.7
Location	Wuhan	182	29.8
	Hubei except Wuhan	325	53.3
	Guangdong	50	8.2
	Heibei	25	4.1
	Yunan	28	4.6
Job type	Doctor	164	26.9
	Nurse	446	73.1
Job title	Junior	379	62.1

For peer review only - http://bmjopen.bmj.com/site/about/guidelines.xhtml

	Intermediate	158	25.9
	Senior	73	12.0
Hospital type	tertiary	325	53.3
	Secondary or other	285	46.7
Working position	Front line	137	22.5
	Secondary line	473	77.5
Assessed mental health services			
psychological support materials	Yes	287	47.0
	No	323	53.0
MHS available through media	Yes	358	58.7
	No	252	41.3
counseling or psychotherapy	Yes	82	13.4
	No	528	86.6

Table 2. Prevalence of acute psychological effects among psychiatric professionals

Variables	Total	Depression			Anxiety			Insomnia			Stress		
		No	Yes	p-value	No	Yes	p-value	No	Yes	p-value	No	Yes	p-value
Total	610												
Physical health				<0.001			<0.001			<0.001			<0.001
Strong	318	125(39.3)	193(60.7)		234(73.6)	84(26.4)		208(65.4)	110(34.6)		192(60.4)	126(39.6)	
Normal	283	24(8.5)	259(91.5)		93(32.9)	190(67.1)		66(23.3)	217(76.7)		76(26.9)	207(73.1)	
Weak	9	0(0)	9(100)		1(11.1)	8(88.9)		0(0)	9(100)		1(11.1)	8(88.9)	
Worry about infection				<0.001			<0.001			<0.001			<0.001

For peer review only - http://bmjopen.bmj.com/site/about/guidelines.xhtml

Yes	480	79(16.5)	401(83.5)		220(45.8)	260(54.2)		189(39.4)	291(60.6)		175(36.5)	305(63.5)	
No	130	70(53.8)	60(46.2)		108(83.1)	22(16.9)		85(65.4)	45(34.6)		94(72.3)	36(27.7)	
Work position				<0.001			<0.001			<0.001			<0.001
Frontline	137	14(10.2)	123(89.8)		46(33.6)	91(66.4)		44(32.1)	93(67.9)		42(30.7)	95(69.3)	
Secondary line	473	135(28.5)	338(71.5)		282(59.6)	191(40.4)		230(48.6)	243(51.4)		227(48)	246(52)	
Self-infection				0.45			0.049			0.12			0.12
No	605	149(24.6)	456(75.4)		328(54.2)	277(45.8)		274(45.3)	331(54.7)		269(44.5)	336(55.5)	
Yes	5	0(0)	5(100)		0(0)	5(100)		0(0)	5(100)		0(0)	5(100)	
Patient infection				<0.001			<0.001			<0.001			<0.001
No	530	145(27.4)	385(72.6)		308(58.1)	222(41.9)		254(47.9)	276(52.1)		255(48.1)	275(51.9)	
Yes	80	4(5)	76(95)		20(25)	60(75)		20(25)	60(75)		14(17.5)	66(82.5)	
Family member infection				0.28			0.84			0.21			0.65
No	603	149(24.7)	454(75.3)		325(53.9)	278(46.1)		273(45.3)	330(54.7)		267(44.3)	336(55.7)	
Yes	7	0(0)	7(100)		3(42.9)	4(57.1)		1(14.3)	6(85.7)		2(28.6)	5(71.4)	
Colleague infection				<0.001			<0.001			0.003			<0.001
No	470	132(28.1)	338(71.9)		283(60.2)	187(39.8)		227(48)	243(52)		230(48.9)	240(51.1)	
Yes	140	17(12.1)	123(87.9)		45(32.1)	95(67.9)		47(34)	93(66)		39(27.9)	101(72.1)	
Friend infection				0.44			0.14			0.18			0.22
No	571	142(24.9)	429(75.1)		312(54.6)	259(45.4)		261(46)	310(54)		256(44.8)	315(55.2)	
Yes	39	7(17.9)	32(82.1)		16(41)	23(59)		13(33)	26(67)		13(33)	26(67)	
Neighbour infection				0.13			0.002			0.61			0.27
No	533	136(25.5)	397(74.5)		300(56.3)	233(43.7)		242(45)	291(55)		240(45)	293(55)	
Yes	77	13(16.8)	64(83.1)		28(36.4)	49(63.6)		32(42)	45(58)		29(37.7)	48(62.3)	
Location				<0.001			<0.001			<0.001			<0.001

For peer review only - http://bmjopen.bmj.com/site/about/guidelines.xhtml

Wuhan	182	20(11)	162(89)		63(34.6)	119(65.4)		54(30)	128(70)		50(27.5)	132(72.5)	
Hubei except Wuhan	325	89(27.4)	236(72.6)		198(60.9)	127(39.1)		167(51)	158(49)		164(50.5)	161(49.5)	
Other provinces except Hubei	103	40(38.8)	63(61.2)		67(65)	36(35)		53(51.5)	50(48.5)		55(53.4)	48(46.6)	
MHS psychological materials				0.01			<0.001			0.004			0.05
No	323	65(20.1)	258(79.9)		152(47.1)	171(52.9)		127(39)	196(61)		130(40.2)	193(59.8)	
Yes	287	84(29.3)	203(70.7)		176(61.3)	111(38.7)		147(51)	140(49)		139(48.4)	148(51.6)	
MHS available through media				0.007			0.002			0.11			0.15
No	252	47(18.7)	205(81.3)		116(46)	136(54)		103(41)	149(59)		102(40.5)	150(59.5)	
Yes	358	102(28.5)	256(71.5)		212(59.2)	146(40.8)		171(48)	187(52)		167(46.6)	191(53.4)	
Information from relatives				0.02			0.02			0.03			<0.001
No	284	82(28.9)	202(71.1)		168(59.2)	116(40.8)		141(50)	143(50)		153(53.9)	131(46.1)	
Yes	326	67(20.6)	259(79.4)		160(49.1)	166(50.9)		133(41)	193(59)		116(35.6)	210(64.4)	
Information from TV				0.001			0.02			0.02			0.004
No	277	50(18.1)	227(81.9)		134(48.4)	143(51.6)		110(40)	167(60)		104(37.5)	173(62.5)	
Yes	333	99(29.7)	234(70.3)		194(58.3)	139(41.7)		164(49)	169(51)		165(49.5)	168(50.5)	

Table 3. Risk & protective factors of mental health of psychiatric professionals identified by stepwise logistic regression

Variables	crude OR (95%CI)	crude P value	adj. OR (95%CI)	P (Wald's test)	P(LR-test)
**PHQ-9**					
gender: male vs female	0.6 (0.39,0.92)	0.019	0.63 (0.38,1.03)	0.063	0.066

For peer review only - http://bmjopen.bmj.com/site/about/guidelines.xhtml

Physical health (cont. var.)	3.9 (2.51,6.06)	< 0.001	2.76 (1.73,4.41)	< 0.001	< 0.001
Patient infection: yes vs no	4.58 (1.81,11.58)	0.001	2.77 (1.05,7.27)	0.039	0.022
MHS media: yes vs no	0.65 (0.43,0.98)	0.039	0.69 (0.43,1.09)	0.112	0.11
MHS psychotherapy: no vs yes	3.84 (0.5,29.46)	0.196	4.05 (0.49,33.61)	0.195	0.127
Having enough personal protection (PP): no vs yes	3.15 (1.78,5.59)	< 0.001	2.43 (1.32,4.47)	0.005	0.003
Worry about infection: no vs yes	0.21 (0.13,0.32)	< 0.001	0.33 (0.21,0.52)	< 0.001	< 0.001
Information from relatives: yes vs no	1.81 (1.22,2.68)	0.003	1.64 (1.03,2.61)	0.036	0.035
Information from TV: yes vs no	0.63 (0.42,0.93)	0.022	0.57 (0.36,0.91)	0.02	0.018
**GAD-7**					
gender: male vs female	0.71 (0.48,1.03)	0.071	0.62 (0.37,1.03)	0.066	0.064
Physical health (cont. var.)	5.62 (3.99,7.92)	< 0.001	4.96 (3.37,7.29)	< 0.001	< 0.001
Job type: doctor vs nurse	0.84 (0.59,1.2)	0.343	0.55 (0.34,0.91)	0.019	0.018
Self-infection: yes vs no	2508169.79 (0,Inf)	0.97	2530278.72 (0,Inf)	0.979	0.045
Patient infection: yes vs no	4.16 (2.44,7.1)	< 0.001	2.36 (1.27,4.39)	0.007	0.006
Colleague infection: yes vs no	3.19 (2.14,4.77)	< 0.001	2.17 (1.31,3.61)	0.003	0.002
Friend infection: yes vs no	1.73 (0.9,3.35)	0.102	0.48 (0.2,1.11)	0.086	0.087
Neighbour infection: yes vs no	2.25 (1.37,3.7)	0.001	2.13 (1.14,3.96)	0.018	0.016
MHS psychological support material: yes vs no	0.56 (0.41,0.77)	< 0.001	0.66 (0.43,0.99)	0.045	0.045
Having enough personal protection (PP): no vs yes	2.22 (1.53,3.23)	< 0.001	1.7 (1.09,2.65)	0.02	0.02
Worry about infection: no vs yes	0.17 (0.11,0.28)	< 0.001	0.25 (0.14,0.44)	< 0.001	< 0.001
Information from TV: yes vs no	0.67 (0.49,0.93)	0.015	0.73 (0.49,1.1)	0.131	0.131
Information from other methods: yes vs no	1.46 (0.91,2.35)	0.119	2.36 (1.29,4.32)	0.005	0.005
Thinking Information helpful? (cont. var.)	1.33 (1.18,1.49)	< 0.001	1.17 (1.01,1.36)	0.035	0.035
**ISI**					

For peer review only - http://bmjopen.bmj.com/site/about/guidelines.xhtml

education (cont. var.)	1.44 (0.82,2.5)	0.201	1.76 (0.95,3.27)	0.073	0.07
Physical health (cont. var.)	6.35 (4.46,9.06)	< 0.001	5.69 (3.93,8.25)	< 0.001	< 0.001
Patient infection: yes vs no	2.76 (1.62,4.71)	< 0.001	1.91 (1.06,3.45)	0.032	0.028
MHS psychological support material: yes vs no	0.62 (0.45,0.85)	0.003	0.74 (0.51,1.07)	0.111	0.111
MHS psychotherapy: no vs yes	1.65 (0.56,4.89)	0.366	2.62 (0.8,8.59)	0.113	0.104
Having enough training: no vs yes	1.95 (1.37,2.79)	< 0.001	1.45 (0.95,2.21)	0.086	0.086
Having enough personal protection (PP): no vs yes	1.99 (1.36,2.91)	< 0.001	1.4 (0.9,2.2)	0.137	0.136
Worry about infection: no vs yes	0.34 (0.23,0.52)	< 0.001	0.57 (0.36,0.91)	0.017	0.017
Information from social media: yes vs no	2.08 (0.75,5.81)	0.16	2.77 (0.82,9.4)	0.102	0.091
Information from TV: yes vs no	0.68 (0.49,0.94)	0.019	0.7 (0.49,1.02)	0.066	0.065
**IES-R**					
education (cont. var.)	1.54 (0.88,2.7)	0.129	1.6 (0.85,3.01)	0.143	0.139
Physical health (cont. var.)	4.1 (2.93,5.74)	< 0.001	3.26 (2.27,4.68)	< 0.001	< 0.001
Patient infection: yes vs no	4.37 (2.4,7.98)	< 0.001	2.91 (1.51,5.58)	0.001	< 0.001
Having enough training: no vs yes	1.92 (1.35,2.75)	< 0.001	1.43 (0.95,2.15)	0.087	0.086
Worry about infection: no vs yes	0.22 (0.14,0.34)	< 0.001	0.34 (0.21,0.54)	< 0.001	< 0.001
Information from relatives: yes vs no	2.11 (1.53,2.93)	< 0.001	2.13 (1.44,3.15)	< 0.001	< 0.001
Information from TV: yes vs no	0.61 (0.44,0.85)	0.003	0.52 (0.35,0.77)	0.001	< 0.001
Think information helpful? (cont. var.)	1.24 (1.1,1.39)	< 0.001	1.15 (1,1.31)	0.043	0.043

For peer review only - http://bmjopen.bmj.com/site/about/guidelines.xhtml

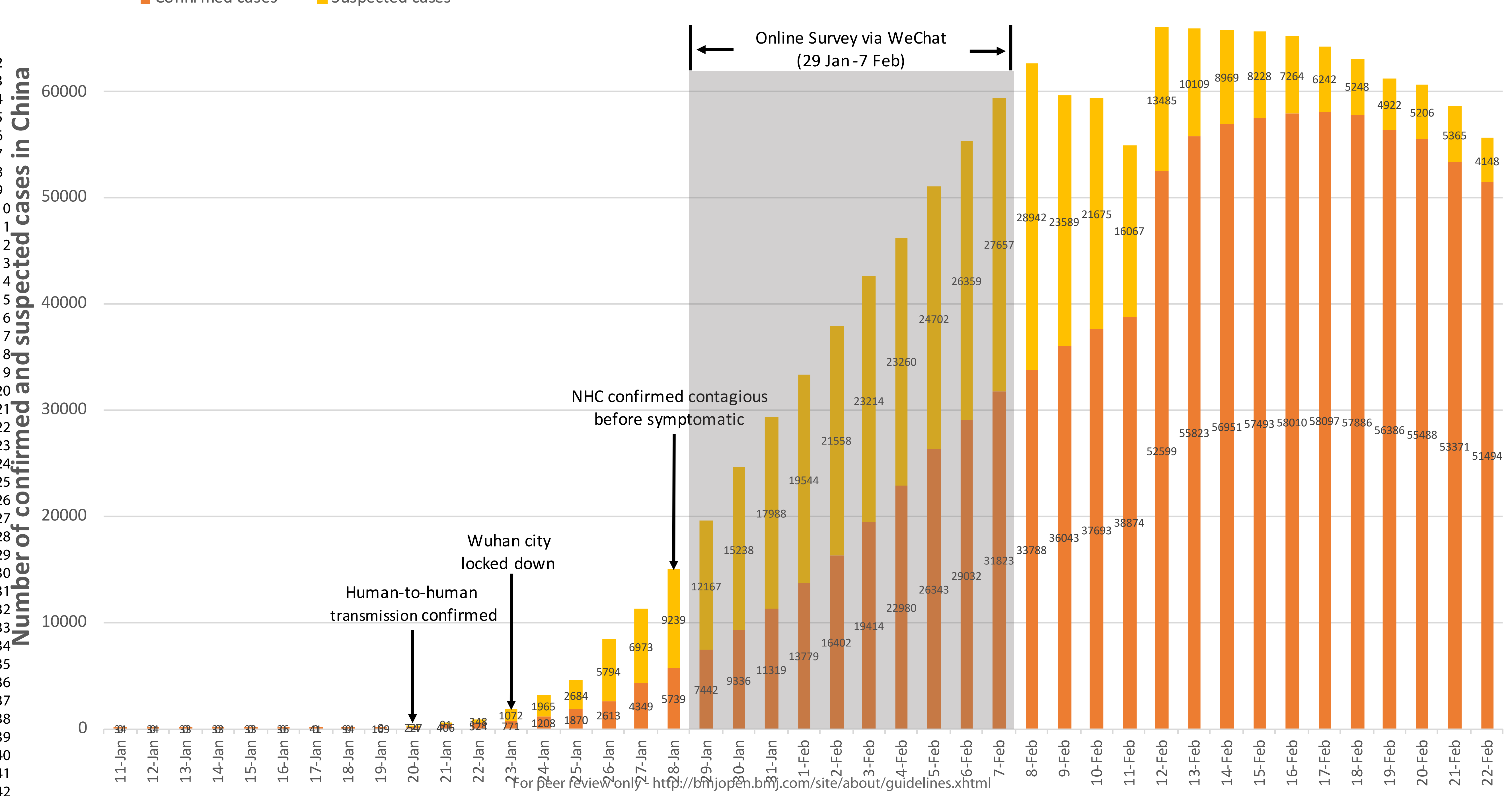
Confirmed cases
Suspected cases
Number of confirmed and suspected cases in China
70000
60000
50000
40000
30000
20000
10000
0
Online Survey via WeChat
(29 Jan -7 Feb)
NHC confirmed contagious
before symptomatic
Wuhan city
locked down
Human-to-human
transmission confirmed
1072
771
1965
2684
1870
5794
2613
6973
4349
9239
5739
12167
7442
15238
9336
17988
11319
19544
13779
21558
16402
23214
19414
23260
22980
24702
26343
26359
29032
27657
31823
28942
33788
23589
36043
21675
37693
16067
38874
13485
52599
10109
55823
8969
56951
8228
57493
7264
58010
6242
58097
5248
57886
4922
56386
5206
55488
5365
53371
4148
51494
11-Jan
12-Jan
13-Jan
14-Jan
15-Jan
16-Jan
17-Jan
18-Jan
19-Jan
20-Jan
21-Jan
22-Jan
23-Jan
24-Jan
25-Jan
26-Jan
27-Jan
28-Jan
29-Jan
30-Jan
31-Jan
1-Feb
2-Feb
3-Feb
4-Feb
5-Feb
6-Feb
7-Feb
8-Feb
9-Feb
10-Feb
11-Feb
12-Feb
13-Feb
14-Feb
15-Feb
16-Feb
17-Feb
18-Feb
19-Feb
20-Feb
21-Feb
22-Feb

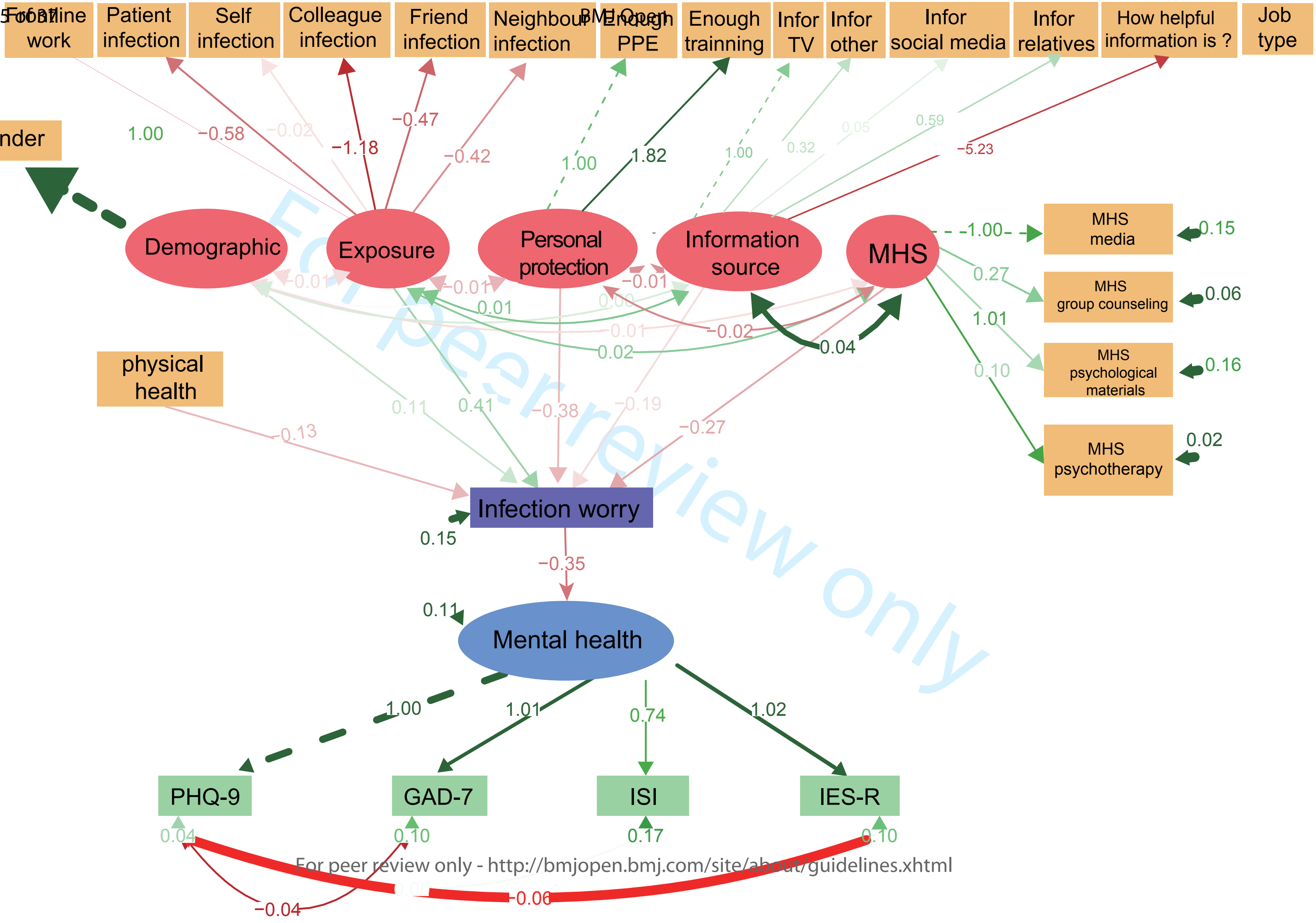

For peer review only

Reporting checklist for cross sectional study.

Based on the STROBE cross sectional guidelines.

Instructions to authors

Complete this checklist by entering the page numbers from your manuscript where readers will find each of the items listed below.

Your article may not currently address all the items on the checklist. Please modify your text to include the missing information. If you are certain that an item does not apply, please write "n/a" and provide a short explanation.

Upload your completed checklist as an extra file when you submit to a journal.

In your methods section, say that you used the STROBE cross sectionalreporting guidelines, and cite them as:

von Elm E, Altman DG, Egger M, Pocock SJ, Gotzsche PC, Vandenbroucke JP. The Strengthening the Reporting of Observational Studies in Epidemiology (STROBE) Statement: guidelines for reporting observational studies.

		Reporting Item	Page Number
**Title and abstract**			
Title	#1a	Indicate the study's design with a commonly used term in the title or the abstract	1
Abstract	#1b	Provide in the abstract an informative and balanced summary of what was done and what was found	3-5
**Introduction**			
Background / rationale	#2	Explain the scientific background and rationale for the investigation being reported	6
Objectives	#3	State specific objectives, including any prespecified hypotheses	6-7
**Methods**			
Study design	#4	Present key elements of study design early in the paper	7-8
Setting	#5	Describe the setting, locations, and relevant dates, including periods of	7-8

For peer review only

		recruitment, exposure, follow-up, and data collection	
Eligibility criteria	#6a	Give the eligibility criteria, and the sources and methods of selection of participants.	7-8
	#7	Clearly define all outcomes, exposures, predictors, potential confounders, and effect modifiers. Give diagnostic criteria, if applicable	8-11
Data sources / measurement	#8	For each variable of interest give sources of data and details of methods of assessment (measurement). Describe comparability of assessment methods if there is more than one group. Give information separately for for exposed and unexposed groups if applicable.	8-11
Bias	#9	Describe any efforts to address potential sources of bias	13
Study size	#10	Explain how the study size was arrived at	13
Quantitative variables	#11	Explain how quantitative variables were handled in the analyses. If applicable, describe which groupings were chosen, and why	8-11
Statistical methods	#12a	Describe all statistical methods, including those used to control for confounding	11-13
Statistical methods	#12b	Describe any methods used to examine subgroups and interactions	12-13
Statistical methods	#12c	Explain how missing data were addressed	
Statistical methods	#12d	If applicable, describe analytical methods taking account of sampling strategy	
Statistical methods	#12e	Describe any sensitivity analyses	
**Results**			
Participants	#13a	Report numbers of individuals at each stage of study—eg numbers potentially eligible, examined for eligibility, confirmed eligible, included in the study, completing follow-up, and analysed. Give information separately for for exposed and unexposed groups if applicable.	13
Participants	#13b	Give reasons for non-participation at each stage	13
Participants	#13c	Consider use of a flow diagram	

For peer review only

Descriptive data	#14a	Give characteristics of study participants (eg demographic, clinical, social) and information on exposures and potential confounders. Give information separately for exposed and unexposed groups if applicable.	
Descriptive data	#14b	Indicate number of participants with missing data for each variable of interest	14
Outcome data	#15	Report numbers of outcome events or summary measures. Give information separately for exposed and unexposed groups if applicable.	14-15
Main results	#16a	Give unadjusted estimates and, if applicable, confounder-adjusted estimates and their precision (eg, 95% confidence interval). Make clear which confounders were adjusted for and why they were included	15-18
Main results	#16b	Report category boundaries when continuous variables were categorized	15-18
Main results	#16c	If relevant, consider translating estimates of relative risk into absolute risk for a meaningful time period	15-18
Other analyses	#17	Report other analyses done—e.g., analyses of subgroups and interactions, and sensitivity analyses	15-18
**Discussion**			
Key results	#18	Summarise key results with reference to study objectives	19-21
Limitations	#19	Discuss limitations of the study, taking into account sources of potential bias or imprecision. Discuss both direction and magnitude of any potential bias.	21
Interpretation	#20	Give a cautious overall interpretation considering objectives, limitations, multiplicity of analyses, results from similar studies, and other relevant evidence.	19-21
Generalisability	#21	Discuss the generalisability (external validity) of the study results	22
**Other Information**			
Funding	#22	Give the source of funding and the role of the funders for the present study and, if applicable, for the original study on which the present article is based	23

None The STROBE checklist is distributed under the terms of the Creative Commons Attribution License CC-BY. This checklist can be completed online using https://www.goodreports.org/, a tool made by the EQUATOR Network in collaboration with Penelope.ai

For peer review only - http://bmjopen.bmj.com/site/about/guidelines.xhtml

BMJ Open

Acute psychological impact of Coronavirus Disease 2019 outbreak among psychiatric professionals in China：A multicenter, cross-sectional, web-based study

Journal:	*BMJ Open*
Manuscript ID	bmjopen-2020-047828.R1
Article Type:	Original research
Date Submitted by the Author:	30-Mar-2021
Complete List of Authors:	Guo, Xin; Renmin Hospital of Wuhan University,<br>McCutcheon, Robert; King's College London<br>Pillinger, Toby; King's College London<br>Arumuham, Atheeshaan; King's College London<br>Chen, Jianhua ; Shanghai Clinical Research Center for Mental Health, Shanghai Key Laboratory of Psychotic Disorders, Shanghai Mental Health Center, Shanghai Jiao Tong University School of Medicine<br>Ma, Simeng ; Renmin Hospital of Wuhan University<br>Yang, Jun ; Huazhong University of Science and Technology<br>Wang, Ying ; Renmin Hospital of Wuhan University<br>Hu, Shaohua ; Zhejiang University, Department of Psychiatry<br>Wang, Gaohua ; Renmin Hospital of Wuhan University<br>Liu, Zhong-Chun; Renmin Hospital of Wuhan University, Department of Psychiatry
**Primary Subject Heading**:	Mental health
Secondary Subject Heading:	Mental health
Keywords:	COVID-19, MENTAL HEALTH, PSYCHIATRY

SCHOLARONE™
Manuscripts

I, the Submitting Author has the right to grant and does grant on behalf of all authors of the Work (as defined in the below author licence), an exclusive licence and/or a non-exclusive licence for contributions from authors who are: i) UK Crown employees; ii) where BMJ has agreed a CC-BY licence shall apply, and/or iii) in accordance with the terms applicable for US Federal Government officers or employees acting as part of their official duties; on a worldwide, perpetual, irrevocable, royalty-free basis to BMJ Publishing Group Ltd ("BMJ") its licensees and where the relevant Journal is co-owned by BMJ to the co-owners of the Journal, to publish the Work in this journal and any other BMJ products and to exploit all rights, as set out in our licence.

The Submitting Author accepts and understands that any supply made under these terms is made by BMJ to the Submitting Author unless you are acting as an employee on behalf of your employer or a postgraduate student of an affiliated institution which is paying any applicable article publishing charge ("APC") for Open Access articles. Where the Submitting Author wishes to make the Work available on an Open Access basis (and intends to pay the relevant APC), the terms of reuse of such Open Access shall be governed by a Creative Commons licence – details of these licences and which Creative Commons licence will apply to this Work are set out in our licence referred to above.

Other than as permitted in any relevant BMJ Author's Self Archiving Policies, I confirm this Work has not been accepted for publication elsewhere, is not being considered for publication elsewhere and does not duplicate material already published. I confirm all authors consent to publication of this Work and authorise the granting of this licence.

For peer review only

Word count: 3641

Acute psychological impact of Coronavirus Disease 2019 outbreak among psychiatric professionals in China：A multicenter, cross-sectional, web-based study

Xin Guo[1,2], Robert McCutcheon[2,3], Toby Pillinger[2,3], Atheeshaan Arumuham[2,3], Jianhua Chen[4], Simeng Ma[1], Jun Yang[5], Ying Wang[1], Shaohua Hu[6], Gaohua Wang[1], Zhongchun Liu[1]

[1]Department of Psychiatry, RenMin Hospital of Wuhan University, Wuhan, China

[2]Department of Psychosis Studies, Institute of Psychiatry, Psychology & Neuroscience, King's college of London, London, UK.

[3]Psychiatric Imaging Group, MRC London Institute of Medical Sciences, Hammersmith Hospital, London, UK

[4]Shanghai Clinical Research Center for Mental Health, Shanghai Key Laboratory of Psychotic Disorders, Shanghai Mental Health Center, Shanghai Jiao Tong University School of Medicine, Shanghai, China.

[5]Computer Science and Technology, Huazhong University of Science and Technology, Wuhan, China.

[6]Department of Psychiatry, First Affiliated Hospital, Zhejiang University School of

For peer review only

Medicine, Hangzhou, China

Corresponding author:

Zhongchun Liu, PhD

Department of Psychiatry, RenMin Hospital of Wuhan University, Wuhan, China

238 Jiefang Road, Wuchang District, Wuhan, China

Email: zcliu6@whu.edu.cn

For peer review only

For peer review only - http://bmjopen.bmj.com/site/about/guidelines.xhtml

Abstract

Objectives: To assess the magnitude of mental health outcomes and associated factors among psychiatric professionals in mental health services during COVID-19 in China.

Design, Setting and Participants: This cross-sectional, survey-based, region-stratified study collected demographic data and mental health measurements from psychiatric professionals in 34 hospitals from 29 January and 7 February 2020, in China. Hospitals equipped with fever clinics or deployed on wards for patients with COVID-19 were eligible.

Primary Outcome and Measures: The degree of symptoms of depression, anxiety, insomnia, and distress was assessed by the Chinese versions of PHQ-9, GAD-7, ISI and IES-R, respectively. Multivariable logistic regression analysis and Structural Equation Model was performed to identify factors associated with mental health outcomes.

Results: A total of 610 psychiatric professionals were included. 29.8% were employed in Wuhan, and 22.5% were frontline workers. A considerable proportion of participants reported symptom of depression (461 [75.6%]), anxiety (282 [46.2%]), insomnia (336 [55.1%]) and mental stress (481 [78.9%]). Their mental health was primarily affected by worrying about infection (e.g. OR 2.36[95% CI 1.27-4.39] for

For peer review only - http://bmjopen.bmj.com/site/about/guidelines.xhtml

anxiety), risks of exposure to COVID-19 (e.g. having inadequate PPE, OR 2.43[1.32-4.47] for depression), self-perceived physical health (e.g. OR 3.22[2.24-4.64] for mental stress). Information sources of COVID-19 may exacerbate (e.g. Information from relatives, OR 2.16[1.46-3.21] for mental stress) or alleviate (e.g. Information from TV, OR 0.52[0.35-0.77] for mental stress) their mental health. Their mental health would benefit from greater availability of mental health care from MHS (e.g. MHS through psychological support materials OR 0.66[0.43-0.99] for anxiety). The SEM analysis indicated that worrying about infection could be the primary mediator that affects their mental health.

Conclusions: These findings emphasize the importance of being prepared to protect the mental health of psychiatric professionals themselves through mental health interventions at times of widespread crisis.

For peer review only

Strengths and limitations of this study

- Provide one of the first comprehensive nationwide evaluation of the acute psychological impact on psychiatric professionals at the early break of COVID-19 in China.
- Provide a set of potential risk and protective factors related to mental health of psychiatric professionals during the pandemic of COVID-19.
- Possible sampling bias exists because of the voluntary response sampling method.
- This cross-sectional study does not record the mental health status of psychiatric professionals before the outbreak or track the efficacy of psychological services among psychiatric professionals.
- A larger sample size is needed to verify the results.

For peer review only - http://bmjopen.bmj.com/site/about/guidelines.xhtml

Introduction

The 2019 epidemic of novel coronavirus disease (COVID-19) that initiated in Wuhan (Hubei Province, China),[1] is now recognized by the World Health Organization (WHO) as a global public health threat. [2]. In addition to the resulting increased burden on physical healthcare services, there is mounting recognition of the impact that the pandemic may have upon public mental health and therefore mental health services. An effectively functioning mental health service requires sufficient levels of staffing, and staff that are in good physical and mental health themselves.

The mental health of health-care workers in general has been affected during the immediate wake of the COVID-19 epidemic.[3] [4] Psychological distress among medical staff has been noted in the form of increased reported symptoms of anxiety, depression, insomnia and posttraumatic stress symptoms [5] [6]. In addition, workers may also experience isolation from their families or community because of infection transmission risk and stigmatization. [7]

The specific impact of the pandemic on the mental health of psychiatric professionals is less well characterised. In the current study, a multicenter survey was performed to assess the psychological distress in psychiatric professionals in China one week after

For peer review only - http://bmjopen.bmj.com/site/about/guidelines.xhtml

Wuhan city locked down. A brief questionnaire was designed and distributed to psychiatric professionals of several hospitals through WeChat group chat application, including the psychiatric professionals serving in psychiatric hospitals and psychiatric departments of general hospitals in Hubei province (including Wuhan) and other provinces, and also including psychiatric professionals working in fever clinics, infectious disease department and other departments. In addition, we investigated which factors are associated with an increased risk of mental health symptoms among psychiatric professionals, and whether there was any evidence for any potential protective factors.

2. Methods

2.1 Online survey

A WeChat-based online survey was carried out by psychiatric professionals between 29 January and 7 February 2020, the second week after Wuhan city locked down. During the survey period, the cumulative number of confirmed and suspected COVID-19 cases was climbing exponentially (see Fig. 1).

The online survey was conducted with the WeChat-based survey program 'Questionnaire Star' based on voluntary response sampling method. The target population was psychiatric professionals working in hospitals that established fever clinics or wards for patients with COVID-19. Each WeChat account of a participant was

For peer review only - http://bmjopen.bmj.com/site/about/guidelines.xhtml

authenticated by their ID card in China. The online advert with a Quick Response (QR) code directing to the online survey was distributed in psychiatric professionals' WeChat working groups of several provinces. Filling the questionnaires outside of working hours was advised. This study was reviewed and approved by the Clinical Research Ethics Committee of Renmin Hospital of Wuhan University (WDRY2020-K004). All subjects provided informed consent electronically prior to registration. Prior to providing informed consent potential participants were provided with information regarding the research project including its primary goals. After reading this information potential participants were provided with the option to either decline or consent to take part in the study. Only those who consented were taken to the questionnaire page. Respondents could terminate the survey at any time or during any question if they wished to.

2.2 Patient and Public Involvement

No patients were involved in this study.

2.3 Questionnaire

The questionnaire consists of seven sections: basic demographic data, mental health assessment, risks of exposure to COVID-19, source of information about COVID-19, mental healthcare services (MHS) accessed, worry about infection and self-perceived physical health before the COVID-19 outbreak. The questionnaire required 15 minutes to complete.

For peer review only - http://bmjopen.bmj.com/site/about/guidelines.xhtml

2.3.1 Demographic data

Basic demographic data include gender, age, marital status (unmarried/other, married), educational level (high school or lower, undergraduate, postgraduate or higher), location, job type (doctor or nurse), job title (junior, intermediate, or senior), and hospital type (tertiary, secondary or other).

2.3.2 Mental health assessment

We used four well validated scales to assess the mental health status of psychiatric professionals. The 9-item Patient Health Questionnaire (PHQ-9), the 7-item Generalized Anxiety Disorder (GAD-7), the 7-item Insomnia Severity Index (ISI) and the 22-item Impact of Event Scale-Revised (IESR) were used to evaluate depression, anxiety, insomnia and stress, respectively. The PHQ-9 is a self-report measure used to assess the severity of depression and has good reliability and validity in Chinese population (Cronbach's α=0.89), [8] with the total scores ranged from 0-27, and a s score of 10 or greater defined as a high probability of meeting diagnostic criteria for depression. [9]The GAD-7 is a self-rated scale to evaluate the severity of anxiety and has good reliability and validity in Chinese population (Cronbach's α=0.89), [10] the total score of GAD-7 ranged from 0 to 21, and a score of greater than 10 was identified as a high probability of meeting diagnostic criteria for anxiety. [11] The ISI is a measure of insomnia severity that has been shown to be valid and reliable, and a total score of greater than 10 was defined as a high probability of meeting diagnostic criteria for

For peer review only - http://bmjopen.bmj.com/site/about/guidelines.xhtml

insomnia. [12] Post traumatic symptoms response to a specific stressful life event were accessed using the IES-R, which also has extensive reliability and validity (Cronbach's α for 3 subscales were 0.89 (Intrusion), 0.85 (Avoidance), and 0.83 (Hyperarousal), respectively), [13] a score of greater than 34 was identified as mental stress. [14]

For peer review only

2.3.3 Risk of exposure to COVID-19

Exposure to COVID-19 was determined with the following questions asked to psychiatric professionals: Have you been diagnosed with COVID-19? Do you manage patients diagnosed with COVID-19? Has your family been diagnosed with COVID-19? Have your friends been diagnosed? Have your neighbors (people living in the same community who may or may not know each other) been diagnosed? Participants were then asked whether there was anyone living with them with suspected symptoms. In terms of personal protection, participants were asked whether they had enough personal protection equipment (PPE) and enough training for personal protection. The possible responses to each question were yes or no.

2.3.4 Accessed mental health services (MHS)

The following question was used to determine which psychological services the subject had accessed: "Have you ever received the following services: psychological support materials (leaflets, brochures and books provided by mental health workers and distributed to staff in the hospital), psychological resources available through

For peer review only - http://bmjopen.bmj.com/site/about/guidelines.xhtml

media (psychological assistance methods and techniques provided by psychologists through social media), or online counseling or psychotherapy (including individual or group counselling, or structural psychotherapy)?".

2.3.5 Source of information about COVID-19

The following question was used to determine from which source the subject had accessed for information regarding COVID-19: "Which way do you used to get information about COVID-19: relatives (information through direct communication with family, neighbors, colleagues, friends or strangers), social media (information from unofficial accounts in WeChat Moments, Weibo, TikTok, and other digital platform), TV (information from national and local official television channels), and other (other information sources of COVID-19) and daily time (hours) spending on information of COVID-19". Then, participants were asked how helpful the information they received were. Answer options included considerably helpful, medium helpful, slightly helpful or not helpful at all.

2.3.6 Self-perceived physical health

Physical health status was determined by asking participants to compare their health status to normal people before the outbreak of COVID-19: How do you perceive your health status compared to normal people before the outbreak? Answer option included strong, normal or weak.

For peer review only - http://bmjopen.bmj.com/site/about/guidelines.xhtml

2.3.7 Worrying about infection

Worry about infection was determined by asking participants if they worry about their vulnerability to infection of COVID-19 in workplace or at home. Possible responses were yes or no.

2.4 Statistical analysis

All statistical analyses were carried out in the statistical programming language R (version-3.6.0). [15]

A bivariate analysis was performed firstly by using Chi-square test to determine the effect of six components (demographic data, risks of exposure to COVID-19, source of information about COVID-19, MHS accessed, worry about infection and self-perceived physical health) on prevalence of four mental health symptoms (depression, anxiety, insomnia and mental stress, assessed by using PHQ-9, GAD-7, ISI, and IES-R separately) among psychiatric professionals.

Stepwise logistic regression analysis according to Akaike information criterion (AIC) was then performed to examine the effects of the six components on four mental health assessment (PHQ-9, GAD-7, ISI, and IES-R scores) separately. The significance of association between the potential risk factors and outcomes were checked by analysis of variance (ANOVA) and prediction accuracy of models were counted by AUC

For peer review only - http://bmjopen.bmj.com/site/about/guidelines.xhtml

(area under ROC curve).

Finally, a structural equation model (SEM) was constructed via R package lavaan 0.6-7 [16] to explore the relationship between mental health assessment (PHQ-9. GAD-7, ISI and IES-R) and the six components, namely demographic data, risks of exposure to COVID-19, source of information about COVID-19, MHS accessed, worry about infection and self-perceived physical health. The estimation method used weighted least squares with mean and variance adjustment test statistics.[17] We used a Monte Carlo method with 1000 guided resamplings to construct a confidence interval for the estimation effect.[18] In SEM, several criteria, such as ratio of value of chi-square test of model fit and degree of freedom (x^2 / df) <3, root mean square error of approximation (RMSEA) values<0.08 and goodness-to-fit index (GFI) values>0.90, indicate acceptable models (Hu and Bentler, 1998).

P values < 0.05 indicated that a difference was statistically significant.

3. Results

Initially, 834 completed questionnaires were received, with a response rate of 78.5%. The duration (seconds) to complete the questionnaire was recorded by Questionnaire star in WeChat. We excluded 119 questionnaires with a complete duration of fewer than 5 minutes (300 seconds) or more than 20 minutes (1,200 seconds), to ensure that

For peer review only - http://bmjopen.bmj.com/site/about/guidelines.xhtml

all questions had been well understood and completed consecutively by respondents. We further excluded 105 questionnaires from provinces that had recruited less than 20 participants. In total, 224 questionnaires were excluded, leaving 610 questionnaires for analysis. Respondents completing the included questionnaires and excluded questionnaires were comparable in age and sex.

3.1 Demographic characteristics

In total, 610 psychiatric professionals, including 164 (26.9%) doctors and 446 (73.1%) nurses were included into analysis. 29.8% of these participants were employed in Wuhan, and 53.3% were employed in Hubei provinces other than Wuhan. 22.5% were frontline mental health staff. 53.3% of participants were working in a tertiary hospital. The participants tended to be female (76.1%), aged 26-40 years (63.1%), married (68.2%), with an educational level of undergraduate (91.3%), and have a junior job title (62.1%), as shown in Table 1.

3.2 Mental health assessment

According to standard questionnaire cut-offs of the PHQ-9, GAD-7, ISI, IES-R scores, 461 (75.6%) of individuals met criteria for depression, 282 (46.2%) for anxiety, 336 (55.1%) for insomnia, and 481 (78.9%) for high levels of mental stress.

3.3 Assessed mental health services (MHS)

36.3% of participants had received psychological support materials, 50.4% had

For peer review only

obtained psychological resources available through media, and 17.5% had participated in online psychological counseling/psychotherapy, as shown in Table 1.

3.3 Risk factors & protective factors of psychological impact of COVID-19 on psychiatric professionals

Bivariate Analysis

We found that psychiatric professionals who took part in frontline work presented higher prevalence of all four mental health symptoms (89.9% vs 71.5%, P<0.001 for depression; 66.4% vs 40.4%, P<0.001 for anxiety; 67.9% vs 51.4%, P<0.001 for insomnia; 69.3% vs 52.0%, P<0.001 for mental stress) compared with those who worked at secondary line. Treating and diagnosing infected patient (95% vs 72.6%, P<0.001 for depression; 75% vs 41.9%, P<0.001 for anxiety; 75% vs 52.1%, P<0.001 for insomnia; 82.5% vs 51.9%, P<0.001 for mental stress), working with infected colleague (87.9% vs 71.9%, P<0.001 for depression; 67.9% vs 39.8%, P<0.001 for anxiety; 66% vs 52%, P<0.001 for insomnia; 72.1% vs 51.1%, P<0.001 for mental stress), employed in Wuhan (Wuhan vs Hubei except Wuhan vs Other provinces except Hubei: 89% vs 72.6% vs 61.2%, P<0.001 for depression; 65.4% vs 39.1% vs 35%, P<0.001 for anxiety; 70% vs 49% vs 48.5%, P<0.001 for insomnia; 72.5% vs 49.5% vs 46.6, P<0.001 for mental stress), worse self-perceived physical health (strong vs normal vs weak: 60.7% vs 91.5% vs 100%, P<0.001 for depression; 26.4% vs 67.1% vs 88.9%, P<0.001 for anxiety; 34.6% vs 76.7% vs 100%, P<0.001 for insomnia; 39.6% vs 73.1% vs 88.9, P<0.001 for mental

For peer review only - http://bmjopen.bmj.com/site/about/guidelines.xhtml

stress), worrying about infection (83.5% vs 46.2%, P<0.001 for depression; 54.2% vs 16.9%, P<0.001 for anxiety; 60.6% vs 34.6%, P<0.001 for insomnia; 63.5% vs 27.7%, P<0.001 for mental stress) and obtaining information about COVID-19 from relatives (79.4% vs 71.1%, P=0.02 for depression; 50.9% vs 40.8%, P=0.02 for anxiety; 59% vs 50%, P=0.03 for insomnia; 64.4% vs 46.1%, P<0.001 for mental stress) were also significantly associated with higher prevalence of all four mental health symptoms. Self-infection (100% vs 45.8%, p=0.049) and living with infected neighbor (65.4% vs 39.1, p=0.002) were significantly associated with higher prevalence of anxiety.

MHS accessed through psychological support materials (70.7% vs 79.9%, P=0.01 for depression; 38.7% vs 52.9%, P<0.001 for anxiety; 49% vs 61%, P=0.004 for insomnia; 51.6% vs 59.8%, P=0.05 for mental stress), MHS accessed through media (71.5% vs 81.3%, P=0.007 for depression; 40.8% vs 54%, P=0.002 for anxiety), and obtaining information about COVID-19 from TV (70.3% vs 81.9%, P=0.001 for depression; 41.7% vs 51.6%, P=0.02 for anxiety; 51% vs 60%, P=0.02 for insomnia; 50.5% vs 62.5%, P=0.004 for mental stress) were significantly associated with lower prevalence of mental health symptoms, as shown in Table 2.

Logistic Regression

To explore the potential risk and protective factors of mental health of psychiatric professionals, stepwise logistic regression analyses on PHQ-9, GAD-7, ISI and IES-R scores separately was performed. After controlling for potential confounding

For peer review only - http://bmjopen.bmj.com/site/about/guidelines.xhtml

variables, checking the significance of effect of each variable using ANOVA, we identified risk and protective factors as follows (Table 3):

PHQ-9

Treating and diagnosing infected patient (odds ratio [OR], 2.77; 95% CI, 1.05-7.27; P = .039), worrying about infection (OR, 0.33; 95% CI, 0.21-0.52; p<0.001), having inadequate PPE (OR, 2.43; 95% CI, 1.32-4.47; p=0.005), and worse self-perceived physical health (OR, 2.76; 95% CI, 1.73-4.41; p<0.001) were significantly associated with higher PHQ-9 score among psychiatric professionals. Obtaining information about COVID-19 from relatives (OR, 1.64; 95% CI, 1.03-2.61; p=0.036) was also significantly associated with increased PHQ-9 score, while obtaining information from TV (OR, 0.57; 95% CI, 0.36-0.91; p=0.02) was significantly associated with lower PHQ-9 score.

GAD-7

Doctors were found to have higher GAD-7 scores (OR, 0.55; 95% CI, 0.34-0.91; p=0.019) compared with nurses. Self-infection seems a primary risk factor to anxiety (OR, 2530278.72; 95% CI, 0-Inf; p=0.045), and all self-infection psychiatric professionals met criteria of severe anxiety. Treating and diagnosing infected patient (OR, 2.36; 95% CI, 1.27-4.39; p=0.007), working with infected colleague (OR, 2.17; 95% CI, 1.31-3.61; p=0.003), living with infected neighbor (OR, 2.13; 95% CI, 1.14-3.96; p=0.018), having inadequate PPE (OR, 1.7; 95% CI, 1.09-2.65; p=0.02), worrying

For peer review only - http://bmjopen.bmj.com/site/about/guidelines.xhtml

about infection (OR, 0.25; 95% CI, 0.14-0.44; p<0.001) and obtaining information from other methods (OR, 2.36; 95% CI, 1.29-4.32; p=0.005) were associated with increased GAD-7 score. MHS accessed through psychological support materials (OR, 0.66; 95% CI, 0.43-0.99; p<0.001) and those who thought information about COVID-19 was helpful (OR, 1.17; 95% CI, 1.01-1.36; p=0.35) showed lower GAD-7 score.

ISI

Treating and diagnosing infected patients (OR, 1.91; 95% CI, 1.06-3.46; p=0.032), worse physical health status (OR, 5.68; 95% CI, 3.92-8.25; p<0.001) and those worrying about infection (OR, 0.58; 95% CI, 0.36-0.92; p=0.02) were significantly associated with higher ISI score.

IES-R

Treating and diagnosing infected patient (OR, 2.97; 95% CI, 1.54-5.73; p=0.001), worse self-perceived physical health (OR, 3.22; 95% CI, 2.24-4.64; p<0.001) and worrying about infection (OR, 0.35; 95% CI, 0.22-0.56; p<0.001) were significantly associated with higher IES-R score among psychiatric professionals. Psychiatric professionals obtaining information from relatives showed significantly higher IES-R score (OR, 2.16; 95% CI, 1.46-3.21; p<0.001), while those who obtained information from TV (OR, 0.52; 95% CI, 0.35-0.77; p=0.001), and those who thought information about COVID-19 was helpful (OR, 0.52; 95% CI, 0.35-0.77; p=0.001) showed lower IES-R score.

For peer review only

For peer review only - http://bmjopen.bmj.com/site/about/guidelines.xhtml

Structural Equation Modelling

Finally, we established an SEM of the association between the seven areas described in methods. The chi-square test of model fit yielded a value of 729.6, with degrees of freedom = 253, P value= 0.000, x^2 / df = 2.88, RMSEA = 0.056, GFI = 0.909, and TLI = 0.973, indicating a good fit. The results showed that gender, the risk factors of exposure, source of information about COVID-19 and self-perceived physical health status affected mental health through worry about infection, indicated that worrying about infection could be the primary mediator that affects the mental health of psychiatric professionals during the COVID-19 outbreak. Mental health services alleviated mental health symptoms through decreasing the worry about infection. The results are shown in Figure 2.

4. Discussion

The present cross-section study firstly investigated the psychological impact of COVID-19 among psychiatric professionals in China during the early outbreak of the pandemic. The overall prevalence of depression, anxiety, insomnia and stress in psychiatric in the second week after Wuhan city locked down were 75.6%, 46.2%, 55.1%, and 78.9%, respectively. In comparison with recent studies of general health care workers [6, 19, 20], the psychological impact of COVID-19 outbreak seem to be more severe among psychiatric professionals compared with general health care workers (the overall

For peer review only - http://bmjopen.bmj.com/site/about/guidelines.xhtml

prevalence of depression, anxiety, insomnia and stress was estimated at 13.9%-46.9%, 16.1%-41.1%, 19.7% and 9.8%-69.1%, respectively). Firstly, most of the participants in the present study were from Wuhan and other cities in Hubei province, according to these recent studies, the prevalence of depression, anxiety and stress among health care workers working in Hubei province (including Wuhan) were estimated at 82.9%-88.7%, 66.9%-80.4%, and 87.6%-92.9%, respectively. Secondly, patients with mental health disorders were believed more susceptible to infections for several reasons, including cognitive impairment, little awareness of risk, and diminished efforts regarding personal protection in patients, also the confined conditions in psychiatric hospital and psychiatric department in general hospital. [21] Our research also determined that the risk of exposure to COVID-19, inadequate personal protection, worrying about being infected and self-perceived physical health were significantly associated with higher risk of mental health symptoms among psychiatric professionals. Thirdly, the psychological impact of COVID-19 in different pandemic stages were different. [19, 20] Our study was performed in the early wake of the outbreak and finished by the end of the second week after Wuhan city locked down, the rapid rising number of identified and suspected cases and unprecedented scale of the city locked down caused public panic and psychological stress. [3] This could exacerbate the mental health problems among health care workers including psychiatric professionals. Furthermore, our study found that females had a higher risk of developing depression symptoms but not anxiety, insomnia or mental stress symptoms during the COVID-19 pandemic among psychiatric professionals. A recent

For peer review only - http://bmjopen.bmj.com/site/about/guidelines.xhtml

meta-analysis study showed that females had a higher risk of developing psychiatric symptoms and reported lower psychological wellbeing during the COVID-19 pandemic, but was not consistent.[22] This may due to various influences such as preconception and fertility issues, pregnancy, postpartum, miscarriage and intimate partner violence.[23] Further studies will be needed to clarify this.

This is the first study to explore the psychological effect of different source of information about COVID-19 on mental health among psychiatric professionals. At the early stage of the outbreak, the unpredictable future of the pandemic was worsened by myths and misinformation, often driven by erroneous news reports from unofficial information sources and the public's misunderstanding of health messages, thus causing worry and increasing the risk of mental health problems in population. [3] The WHO uses the term "infodemic" to describe the impossible amounts of misinformation and disinformation in worldwide, and the doctors and scientists have long been called on to share their wisdom through the official media. [24] [25] Our study determined that information from social media, direct communication with relatives, and other unofficial information sources increased risk of mental health symptoms among psychiatric professionals. The information of COVID-19 from official source such as TV seems a protective factor for mental health among psychiatric professionals, thus indicating that trustable sources of reliable and accurate information are not just helpful for epidemic control, but also good to mental health.

For peer review only - http://bmjopen.bmj.com/site/about/guidelines.xhtml

To efficiently cope with psychological impact of the COVID-19 outbreak, the Chinese Government has implemented rapid and comprehensive psychological crisis intervention, including publishing psychological support materials, such as leaflets and books, establishing psychological intervention teams on site [4] , mental health service online through social media platform, and also telephone guidance to help address mental health problems.[26] Our study found that psychiatric professionals had benefited from availability of mental health care from public mental health services (psychological materials and psychological help from media) and personalized mental health services (online counseling and psychotherapy).

Our study also has some limitations. First, the study is cross-sectional and does not record the mental health status before the outbreak, or track the efficacy of psychological services among psychiatric professionals. Second, most respondents (76.1%) in this study were made up by female, this may due to the overall psychiatric workforce was predominantly nurses (73.1%). As by 2019, the ratio of psychiatric doctors to nurses in China was close to 1:2.1, [27] the ratio of doctors to nurses in this study is 1:2.7, while a response bias cannot be ruled out the available data regarding the demographics of the workforce suggests. Third, a larger sample size is needed to verify the results.

In summary, the results demonstrate that a strikingly large portion of psychiatric professionals in China suffered from mental health disturbances during the early break of COVID-19. Their mental health was primarily affected by worry about being infected,

For peer review only - http://bmjopen.bmj.com/site/about/guidelines.xhtml

risks of exposure to COVID-19, self-perceived physical health. Unreliable information sources of COVID-19 may exacerbate their mental health symtoms, and their mental health would benefit from greater availability of mental health care from public and personalized mental health services. Among the steps needed to better prepare for future infectious disease outbreaks would be a greater investment in the infectious diseases' prevention and control in psychiatric hospitals and psychiatric department in general hospitals, and in the mental health services among psychiatric professionals' themselves to protect and care for future psychiatric professionals who find themselves unexpectedly on the dangerous front lines of disease response.

Acknowledgments

Author Contribution: Dr Guo, Dr McCutcheon and Prof Liu had full access to all the data in the study and takes responsibility for the integrity of the data and the accuracy of the data analysis.

Design and conduct of the study: Dr Guo, Dr McCutcheon, Prof Liu.

Collection, management, analysis, and interpretation of the data: Dr Guo, Dr McCutcheon, Dr Pillinger, Dr Arumuham, Dr Chen, Dr Ma, Dr Yang, Dr Wang, Dr Hu.

Preparation, review, or approval of the manuscript: Dr Guo, Dr McCutcheon, Dr Arumuham, Dr Chen, Prof Liu and Prof Wang.

Decision to submit the manuscript for publication: Prof Liu and Prof Wang.

For peer review only - http://bmjopen.bmj.com/site/about/guidelines.xhtml

Funding/Support: XG's work is funded by the National Key R&D Program of China (no. 2018YFC1314600), and fellowship grants from Chinese Scholarship Council. ZCL's work is funded by the National Key R&D Program of China (no. 2018YFC1314600). RM's research is funded by the Wellcome Trust (no. 200102/Z/15/Z) and a NIHR clinical lectureship.

Ethical Approval Statement

This study was reviewed and approved by the Clinical Research Ethics Committee of Renmin Hospital of Wuhan University (WDRY2020-K004).

Data availability statement

No additional data available.

Declaration of interest:

The authors declare that they have no conflict of interest.

References

1 Chan JF, Yuan S, Kok KH, et al. A familial cluster of pneumonia associated with the 2019 novel coronavirus indicating person-to-person transmission: a study of a family cluster. *Lancet* 2020;395(1474-547X (Electronic)):514-23.

2 WHO. Statement on the second meeting of the International Health Regulations (2005) Emergency Committee regarding the outbreak of novel coronavirus (2019-nCoV). https://www.who.int/news-room/detail/30-01-2020-statement-on-thesecond-meeting-of-the-international-health-regulations-(2005)-emergency-committee-regarding-the-outbreak-of-novel-coronavirus-(2019-ncov) (assessed Feb 15, 2020). 2020

For peer review only - http://bmjopen.bmj.com/site/about/guidelines.xhtml

3 Bao Y, Sun Y, Meng S, et al. 2019-nCoV epidemic: address mental health care to empower society. *Lancet* 2020;395(1474-547X (Electronic)):e37-e38.

4 Kang L, Li Y, Hu S, et al. The mental health of medical workers in Wuhan, China dealing with the 2019 novel coronavirus. *Lancet Psychiatry* 2020(2215-0374 (Electronic)) doi: https://doi.org/10.1016/s2215-0366(20)30047-x

5 da Silva FCT, Neto MLR. Psychiatric symptomatology associated with depression, anxiety, distress, and insomnia in health professionals working in patients affected by COVID-19: A systematic review with meta-analysis. *Prog Neuropsychopharmacol Biol Psychiatry* 2020(1878-4216 (Electronic)) doi: 10.1016/j.pnpbp.2020.110057

6 Lin K, Yang BX, Luo D, et al. The Mental Health Effects of COVID-19 on Health Care Providers in China. *American Journal of Psyhciatry* 2020(1535-7228 (Electronic))

7 Van Bortel T, Basnayake A, Wurie F, et al. Psychosocial effects of an Ebola outbreak at individual, community and international levels. *Bull World Health Organ* 2016;94(1564-0604 (Electronic)):210-14.

8 Chen S, Fang Y, Chiu H, et al. Validation of the nine-item Patient Health Questionnaire to screen for major depression in a Chinese primary care population. *Asia Pac Psychiatry* 2013;5(1758-5872 (Electronic)):61-68.

9 Manea L, Gilbody S, McMillan D. Optimal cut-off score for diagnosing depression with the Patient Health Questionnaire (PHQ-9): a meta-analysis. *Canadian Medical Association Journal* 2012;184(1488-2329 (Electronic)):e191-e96.

10 Tong X, An D, McGonigal A, et al. Validation of the Generalized Anxiety Disorder-7 (GAD-7) among Chinese people with epilepsy. *Epilepsy Res* 2015(1872-6844 (Electronic))

11 Spitzer R, Kroenke K, Williams J, et al. A brief measure for assessing generalized anxiety disorder: the GAD-7. *Arch Intern Med* 2006;166(0003-9926 (Print)):1092-97.

12 Morin C, Belleville G, Bélanger L, et al. The Insomnia Severity Index: psychometric indicators to detect insomnia cases and evaluate treatment response. *Sleep* 2011;34(1550-9109 (Electronic)):601-08.

13 Wu K, Chan K. The development of the Chinese version of Impact of Event Scale--Revised (CIES-R). *Soc Psychiatry Psychiatr Epidemiol* 2003;38(0933-7954 (Print)):94-98.

14 Morina N, Ehring T, Priebe S. Diagnostic utility of the impact of event scale-revised in two samples of survivors of war. *PLoS One* 2013;31(1932-6203 (Electronic)):e83916.

15 Team RC. R: A Language and Environment for Statistical Computing. 2013

16 Rosseel Y. Lavaan: A R package for structural equation modeling. *Journal of Statistical Software* 2012;48(2):1-36.

17 DiStefano C, Morgan GB. A Comparison of Diagonal Weighted Least Squares Robust Estimation Techniques for Ordinal Data. *Structural Equation Modeling: A Multidisciplinary Journal* 2014;21(3):425-38. doi: 10.1080/10705511.2014.915373

18 Bauer DJ, Preacher KJ, Gil KM. Conceptualizing and testing random indirect effects and moderated mediation in multilevel models: new procedures and recommendations. *Psychol Methods* 2006;11(2):142-63. doi: 10.1037/1082-989x.11.2.142 [published Online First: 2006/06/21]

19 Wang Y, Ma S, Yang C, et al. Acute psychological effects of Coronavirus Disease 2019 outbreak among healthcare workers in China: a cross-sectional study. *Transl Psychiatry* 2020;10(2158-3188 (Electronic)):348.

20 Wang L, Zhang M, Liu G, et al. Psychological impact of coronavirus disease (2019) (COVID-19)

For peer review only - http://bmjopen.bmj.com/site/about/guidelines.xhtml

epidemic on medical staff in different posts in China: A multicenter study. *Journal of Psychiatric Research* 2020;129(1879-1379 (Electronic)):198-205.

21 Yao H, Chen JH, Xu YF. Patients with mental health disorders in the COVID-19 epidemic. *Lancet Psychiatry* 2020;7(2215-0374 (Electronic)):e21.

22 Vindegaard N, Benros ME. COVID-19 pandemic and mental health consequences: Systematic review of the current evidence. *Brain, Behavior, and Immunity* 2020;89(1090-2139 (Electronic)):531-42.

23 Almeida MA-O, Shrestha AD, Stojanac D, et al. The impact of the COVID-19 pandemic on women's mental health. *Archives of Women's Mental Health* 2020(1435-1102 (Electronic))

24 Cinelli M, Quattrociocchi W, Galeazzi A, et al. The COVID-19 social media infodemic. *Sci Rep* 2020(2045-2322 (Electronic)) doi: 10.1038/s41598-020-73510-5

25 Diseases TLI. The COVID-19 infodemic. *Lancet* 2020;20(8):875.

26 Liu S, Yang L, Zhang C, et al. Online mental health services in China during the COVID-19 outbreak. *Lancet Psychiatry* 2020(2215-0374 (Electronic)) doi: 10.1016/S2215-0366(20)30077-8

27 Lin L. The retrospect and prospect of psychiatry in China. *Journal of Peking University (Health Sciences)* 2019;51(3):379-83. doi: 10.19723/j.issn.1671-167X.2019.03.001

For peer review only - http://bmjopen.bmj.com/site/about/guidelines.xhtml

Figure & Table Legend

Figure 1. Timeline of COVID-19 and survey data. Wuhan city was locked down since 23 Jan 2020, and the online survey was conducted 1 week after that. The cumulative number seemed to have entered the rapid rise phase.

------------------------------ Figure 1----------------------------

Figure 2. Direct and indirect effects in SEM. The solid line represents a significant relationship between the two, while the dotted line represents the relationship is not significant.

------------------------------ Figure 2---------------------------

For peer review only - http://bmjopen.bmj.com/site/about/guidelines.xhtml

Table 1. Demographic characteristics and accessed mental health services

Variables		Number	Percentage (%)
Total		610	100
Gender	Female	464	76.1
	Male	146	23.9
Age (years)	18-25	92	15.1
	26-30	198	32.5
	31-40	187	30.7
	>40	133	21.8
Marital status	Unmarried/Other	194	31.8
	Married	416	68.2
Education level	High school or less	18	3.0
	Undergraduate	557	91.3
	Graduate or higher	35	5.7
Location	Wuhan	182	29.8
	Hubei except Wuhan	325	53.3
	Guangdong	50	8.2
	Heibei	25	4.1
	Yunan	28	4.6
Job type	Doctor	164	26.9
	Nurse	446	73.1
Job title	Junior	379	62.1

For peer review only - http://bmjopen.bmj.com/site/about/guidelines.xhtml

	Intermediate	158	25.9
	Senior	73	12.0
Hospital type	tertiary	325	53.3
	Secondary or other	285	46.7
Working position	Front line	137	22.5
	Secondary line	473	77.5
Assessed mental health services			
psychological support materials	Yes	287	47.0
	No	323	53.0
MHS available through media	Yes	358	58.7
	No	252	41.3
counseling or psychotherapy	Yes	82	13.4
	No	528	86.6

Table 2. Prevalence of acute psychological effects among psychiatric professionals

Variables	Total	PHQ-9			GAD-7			ISI			IES-R		
		No	Yes	p-value	No	Yes	p-value	No	Yes	p-value	No	Yes	p-value
Total	610												
Gender				0.002			0.09			0.26			0.08
Male	146	50(34.2)	96(65.8)		88(60.3)	58(39.7)		72(49.3)	74(50.7)		74(50.7)	72(49.3)	
Female	464	99(21.3)	365(78.7)		240(51.7)	224(48.3)		202(43.5)	262(56.5)		195(42.0)	269(58.0)	
Physical health				<0.001			<0.001			<0.001			<0.001
Strong	318	125(39.3)	193(60.7)		234(73.6)	84(26.4)		208(65.4)	110(34.6)		192(60.4)	126(39.6)	

For peer review only

Normal	283	24(8.5)	259(91.5)		93(32.9)	190(67.1)		66(23.3)	217(76.7)		76(26.9)	207(73.1)	
Weak	9	0(0)	9(100)		1(11.1)	8(88.9)		0(0)	9(100)		1(11.1)	8(88.9)	
Worrying about infection				<0.001			<0.001			<0.001			<0.001
Yes	480	79(16.5)	401(83.5)		220(45.8)	260(54.2)		189(39.4)	291(60.6)		175(36.5)	305(63.5)	
No	130	70(53.8)	60(46.2)		108(83.1)	22(16.9)		85(65.4)	45(34.6)		94(72.3)	36(27.7)	
Work position				<0.001			<0.001			<0.001			<0.001
Frontline	137	14(10.2)	123(89.8)		46(33.6)	91(66.4)		44(32.1)	93(67.9)		42(30.7)	95(69.3)	
Secondary line	473	135(28.5)	338(71.5)		282(59.6)	191(40.4)		230(48.6)	243(51.4)		227(48)	246(52)	
Self-infection				0.45			0.049			0.12			0.12
No	605	149(24.6)	456(75.4)		328(54.2)	277(45.8)		274(45.3)	331(54.7)		269(44.5)	336(55.5)	
Yes	5	0(0)	5(100)		0(0)	5(100)		0(0)	5(100)		0(0)	5(100)	
Patient infection				<0.001			<0.001			<0.001			<0.001
No	530	145(27.4)	385(72.6)		308(58.1)	222(41.9)		254(47.9)	276(52.1)		255(48.1)	275(51.9)	
Yes	80	4(5)	76(95)		20(25)	60(75)		20(25)	60(75)		14(17.5)	66(82.5)	
Family member infection				0.28			0.84			0.21			0.65
No	603	149(24.7)	454(75.3)		325(53.9)	278(46.1)		273(45.3)	330(54.7)		267(44.3)	336(55.7)	
Yes	7	0(0)	7(100)		3(42.9)	4(57.1)		1(14.3)	6(85.7)		2(28.6)	5(71.4)	
Colleague infection				<0.001			<0.001			0.003			<0.001
No	470	132(28.1)	338(71.9)		283(60.2)	187(39.8)		227(48)	243(52)		230(48.9)	240(51.1)	
Yes	140	17(12.1)	123(87.9)		45(32.1)	95(67.9)		47(34)	93(66)		39(27.9)	101(72.1)	
Friend infection				0.44			0.14			0.18			0.22
No	571	142(24.9)	429(75.1)		312(54.6)	259(45.4)		261(46)	310(54)		256(44.8)	315(55.2)	
Yes	39	7(17.9)	32(82.1)		16(41)	23(59)		13(33)	26(67)		13(33)	26(67)	

For peer review only - http://bmjopen.bmj.com/site/about/guidelines.xhtml

Neighbour infection				0.13			0.002			0.61			0.27
No	533	136(25.5)	397(74.5)		300(56.3)	233(43.7)		242(45)	291(55)		240(45)	293(55)	
Yes	77	13(16.8)	64(83.1)		28(36.4)	49(63.6)		32(42)	45(58)		29(37.7)	48(62.3)	
Location				<0.001			<0.001			<0.001			<0.001
Wuhan	182	20(11)	162(89)		63(34.6)	119(65.4)		54(30)	128(70)		50(27.5)	132(72.5)	
Hubei except Wuhan	325	89(27.4)	236(72.6)		198(60.9)	127(39.1)		167(51)	158(49)		164(50.5)	161(49.5)	
Other provinces except Hubei	103	40(38.8)	63(61.2)		67(65)	36(35)		53(51.5)	50(48.5)		55(53.4)	48(46.6)	
MHS psychological materials				0.01			<0.001			0.004			0.05
No	323	65(20.1)	258(79.9)		152(47.1)	171(52.9)		127(39)	196(61)		130(40.2)	193(59.8)	
Yes	287	84(29.3)	203(70.7)		176(61.3)	111(38.7)		147(51)	140(49)		139(48.4)	148(51.6)	
MHS available through media				0.007			0.002			0.11			0.15
No	252	47(18.7)	205(81.3)		116(46)	136(54)		103(41)	149(59)		102(40.5)	150(59.5)	
Yes	358	102(28.5)	256(71.5)		212(59.2)	146(40.8)		171(48)	187(52)		167(46.6)	191(53.4)	
Information from relatives				0.02			0.02			0.03			<0.001
No	284	82(28.9)	202(71.1)		168(59.2)	116(40.8)		141(50)	143(50)		153(53.9)	131(46.1)	
Yes	326	67(20.6)	259(79.4)		160(49.1)	166(50.9)		133(41)	193(59)		116(35.6)	210(64.4)	
Information from TV				0.001			0.02			0.02			0.004
No	277	50(18.1)	227(81.9)		134(48.4)	143(51.6)		110(40)	167(60)		104(37.5)	173(62.5)	
Yes	333	99(29.7)	234(70.3)		194(58.3)	139(41.7)		164(49)	169(51)		165(49.5)	168(50.5)	

For peer review only

For peer review only - http://bmjopen.bmj.com/site/about/guidelines.xhtml

Table 3. Risk & protective factors of mental health of psychiatric professionals identified by stepwise logistic regression

Variables	crude OR (95%CI)	crude P value	adj. OR (95%CI)	P (Wald's test)	P(LR-test)
**PHQ-9**					
gender: male vs female	0.6 (0.39,0.92)	0.019	0.63 (0.38,1.03)	0.063	0.066
Physical health (cont. var.)	3.9 (2.51,6.06)	< 0.001	2.76 (1.73,4.41)	< 0.001	< 0.001
Patient infection: yes vs no	4.58 (1.81,11.58)	0.001	2.77 (1.05,7.27)	0.039	0.022
MHS media: yes vs no	0.65 (0.43,0.98)	0.039	0.69 (0.43,1.09)	0.112	0.11
MHS psychotherapy: no vs yes	3.84 (0.5,29.46)	0.196	4.05 (0.49,33.61)	0.195	0.127
Having enough personal protection (PP): no vs yes	3.15 (1.78,5.59)	< 0.001	2.43 (1.32,4.47)	0.005	0.003
Worrying about infection: no vs yes	0.21 (0.13,0.32)	< 0.001	0.33 (0.21,0.52)	< 0.001	< 0.001
Information from relatives: yes vs no	1.81 (1.22,2.68)	0.003	1.64 (1.03,2.61)	0.036	0.035
Information from TV: yes vs no	0.63 (0.42,0.93)	0.022	0.57 (0.36,0.91)	0.02	0.018
**GAD-7**					
gender: male vs female	0.71 (0.48,1.03)	0.071	0.62 (0.37,1.03)	0.066	0.064
Physical health (cont. var.)	5.62 (3.99,7.92)	< 0.001	4.96 (3.37,7.29)	< 0.001	< 0.001
Job type: doctor vs nurse	0.84 (0.59,1.2)	0.343	0.55 (0.34,0.91)	0.019	0.018
Self-infection: yes vs no	2508169.79 (0,Inf)	0.97	2530278.72 (0,Inf)	0.979	0.045
Patient infection: yes vs no	4.16 (2.44,7.1)	< 0.001	2.36 (1.27,4.39)	0.007	0.006
Colleague infection: yes vs no	3.19 (2.14,4.77)	< 0.001	2.17 (1.31,3.61)	0.003	0.002
Friend infection: yes vs no	1.73 (0.9,3.35)	0.102	0.48 (0.2,1.11)	0.086	0.087
Neighbour infection: yes vs no	2.25 (1.37,3.7)	0.001	2.13 (1.14,3.96)	0.018	0.016
MHS psychological support material: yes vs no	0.56 (0.41,0.77)	< 0.001	0.66 (0.43,0.99)	0.045	0.045
Having enough personal protection (PP): no vs yes	2.22 (1.53,3.23)	< 0.001	1.7 (1.09,2.65)	0.02	0.02
Worryinng about infection: no vs yes	0.17 (0.11,0.28)	< 0.001	0.25 (0.14,0.44)	< 0.001	< 0.001

For peer review only

Information from TV: yes vs no	0.67 (0.49,0.93)	0.015	0.73 (0.49,1.1)	0.131	0.131
Information from other methods: yes vs no	1.46 (0.91,2.35)	0.119	2.36 (1.29,4.32)	0.005	0.005
Thinking Information helpful? (cont. var.)	1.33 (1.18,1.49)	< 0.001	1.17 (1.01,1.36)	0.035	0.035
**ISI**					
gender: male vs female	0.79 (0.55,1.15)	0.221	0.97 (0.62,1.5)	0.88	0.88
education (cont. var.)	1.44 (0.82,2.5)	0.201	1.76 (0.95,3.27)	0.073	0.07
Physical health (cont. var.)	6.35 (4.46,9.06)	< 0.001	5.68 (3.92,8.25)	< 0.001	< 0.001
Patient infection: yes vs no	2.76 (1.62,4.71)	< 0.001	1.91 (1.06,3.46)	0.032	0.027
MHS psychological support material: yes vs no	0.62 (0.45,0.85)	0.003	0.74 (0.51,1.07)	0.111	0.111
MHS psychotherapy: no vs yes	1.65 (0.56,4.89)	0.366	2.63 (0.8,8.63)	0.112	0.103
Having enough training: no vs yes	1.95 (1.37,2.79)	< 0.001	1.45 (0.95,2.21)	0.088	0.087
Having enough personal protection (PP): no vs yes	1.99 (1.36,2.91)	< 0.001	1.4 (0.9,2.2)	0.135	0.134
Worrying about infection: no vs yes	0.34 (0.23,0.52)	< 0.001	0.58 (0.36,0.92)	0.020	0.019
Information from social media: yes vs no	2.08 (0.75,5.81)	0.16	2.76 (0.81,9.4)	0.103	0.093
Information from TV: yes vs no	0.68 (0.49,0.94)	0.019	0.7 (0.49,1.02)	0.067	0.066
**IES-R**					
education (cont. var.)	1.54 (0.88,2.7)	0.129	1.58 (0.84,2.97)	0.154	0.15
gender: male vs female	0.71 (0.49,1.02)	0.067	0.81 (0.52,1.25)	0.34	0.341
Physical health (cont. var.)	4.1 (2.93,5.74)	< 0.001	3.22 (2.24,4.64)	< 0.001	< 0.001
Patient infection: yes vs no	4.37 (2.4,7.98)	< 0.001	2.97 (1.54,5.73)	0.001	< 0.001
Having enough training: no vs yes	1.92 (1.35,2.75)	< 0.001	1.42 (0.95,2.14)	0.089	0.089
Worrying about infection: no vs yes	0.22 (0.14,0.34)	< 0.001	0.35 (0.22,0.56)	< 0.001	< 0.001
Information from relatives: yes vs no	2.11 (1.53,2.93)	< 0.001	2.16 (1.46,3.21)	< 0.001	< 0.001
Information from TV: yes vs no	0.61 (0.44,0.85)	0.003	0.52 (0.35,0.77)	0.001	< 0.001

For peer review only

Think information helpful? (cont. var.)	1.24 (1.1,1.39)	< 0.001	1.15 (1,1.31)	0.045	0.044

1

For peer review only
For peer review only - http://bmjopen.bmj.com/site/about/guidelines.xhtml

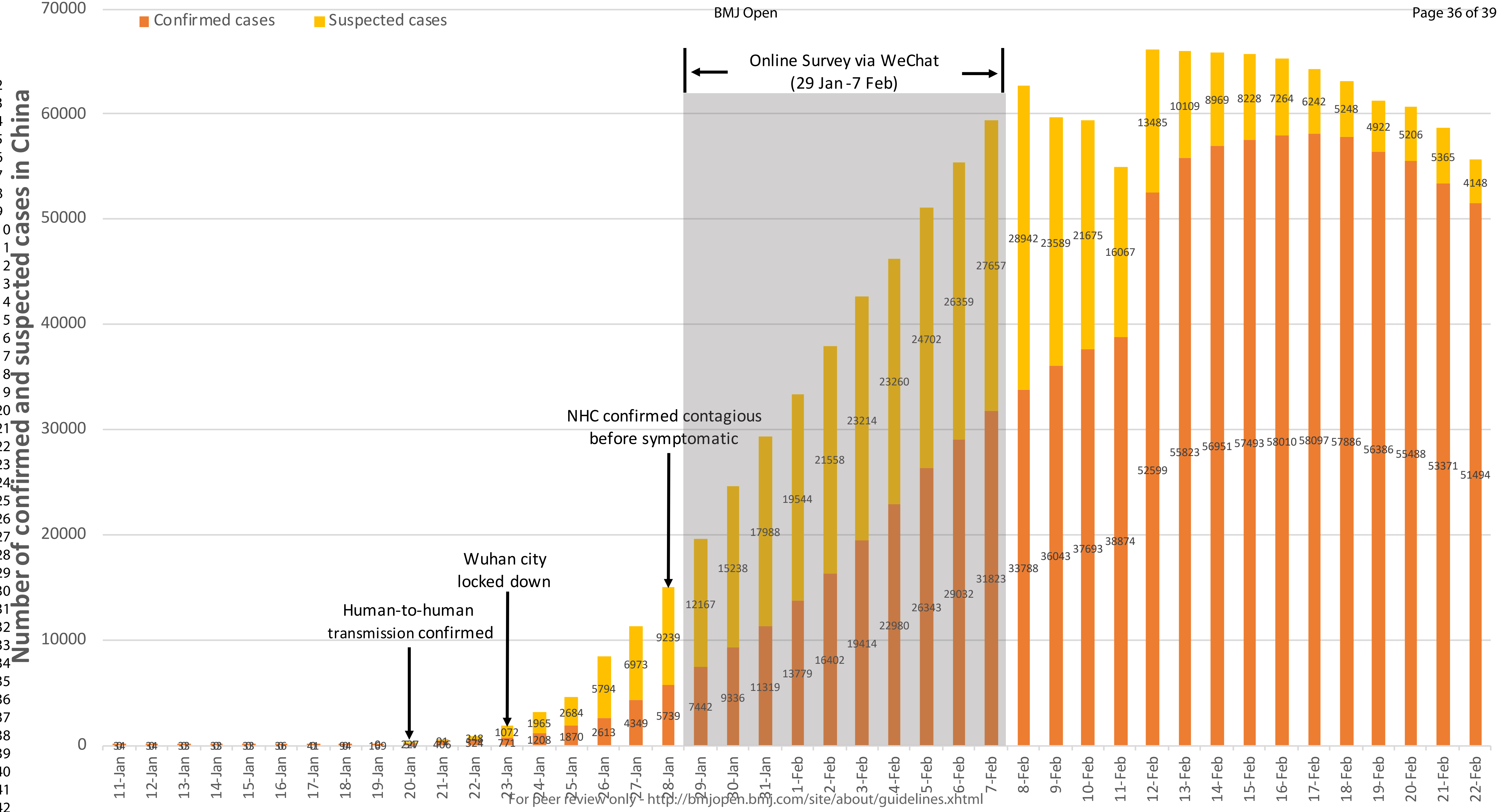
Confirmed cases
Suspected cases
Number of confirmed and suspected cases in China
Online Survey via WeChat
(29 Jan -7 Feb)
NHC confirmed contagious
before symptomatic
Wuhan city
locked down
Human-to-human
transmission confirmed

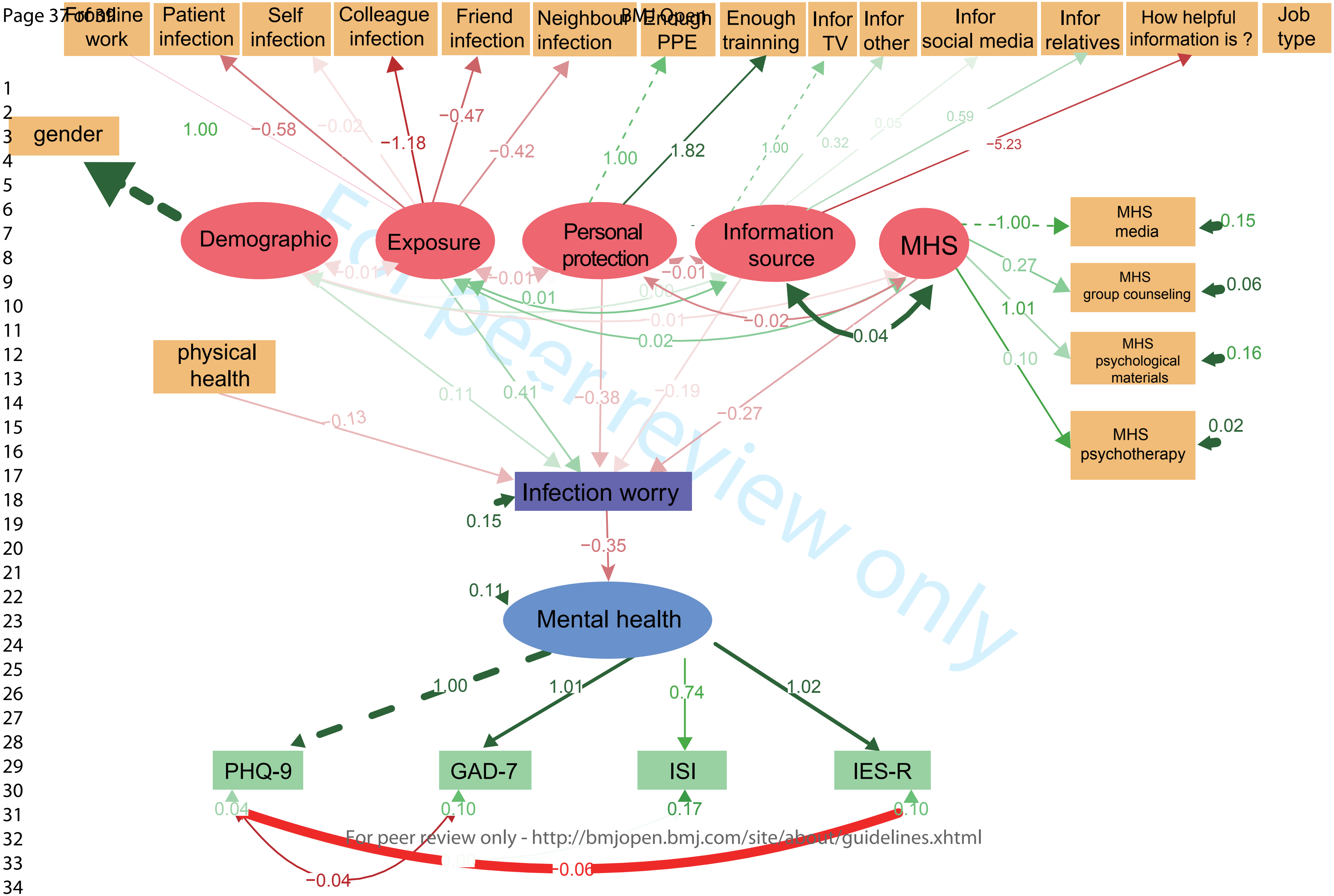
Frontline work
Patient infection
Self infection
Colleague infection
Friend infection
Neighbour infection
Enough PPE
Enough trainning
Infor TV
Infor other
Infor social media
Infor relatives
How helpful information is ?
Job type
gender
Demographic
Exposure
Personal protection
Information source
MHS
MHS media
MHS group counseling
MHS psychological materials
MHS psychotherapy
physical health
Infection worry
Mental health
PHQ-9
GAD-7
ISI
IES-R
1.00
−0.58
−0.02
−1.18
−0.47
−0.42
1.00
1.82
1.00
0.32
0.05
0.59
−5.23
−0.01
−0.01
−0.01
0.01
0.00
−0.01
−0.02
0.02
0.04
1.00
0.15
0.27
0.06
1.01
0.16
0.10
0.02
0.11
0.41
−0.38
−0.19
−0.27
−0.13
0.15
−0.35
0.11
1.00
1.01
0.74
1.02
0.04
0.10
0.17
0.10
−0.04
−0.06
For peer review only

Reporting checklist for cross sectional study.

Based on the STROBE cross sectional guidelines.

Instructions to authors

Complete this checklist by entering the page numbers from your manuscript where readers will find each of the items listed below.

Your article may not currently address all the items on the checklist. Please modify your text to include the missing information. If you are certain that an item does not apply, please write "n/a" and provide a short explanation.

Upload your completed checklist as an extra file when you submit to a journal.

In your methods section, say that you used the STROBE cross sectionalreporting guidelines, and cite them as:

von Elm E, Altman DG, Egger M, Pocock SJ, Gotzsche PC, Vandenbroucke JP. The Strengthening the Reporting of Observational Studies in Epidemiology (STROBE) Statement: guidelines for reporting observational studies.

For peer review only

		Reporting Item	Page Number
**Title and abstract**			
Title	#1a	Indicate the study's design with a commonly used term in the title or the abstract	1
Abstract	#1b	Provide in the abstract an informative and balanced summary of what was done and what was found	3-5
**Introduction**			
Background / rationale	#2	Explain the scientific background and rationale for the investigation being reported	6
Objectives	#3	State specific objectives, including any prespecified hypotheses	6-7
**Methods**			
Study design	#4	Present key elements of study design early in the paper	7-8
Setting	#5	Describe the setting, locations, and relevant dates, including periods of	7-8

		recruitment, exposure, follow-up, and data collection	
Eligibility criteria	#6a	Give the eligibility criteria, and the sources and methods of selection of participants.	7-8
	#7	Clearly define all outcomes, exposures, predictors, potential confounders, and effect modifiers. Give diagnostic criteria, if applicable	8-11
Data sources / measurement	#8	For each variable of interest give sources of data and details of methods of assessment (measurement). Describe comparability of assessment methods if there is more than one group. Give information separately for for exposed and unexposed groups if applicable.	8-11
Bias	#9	Describe any efforts to address potential sources of bias	13
Study size	#10	Explain how the study size was arrived at	13
Quantitative variables	#11	Explain how quantitative variables were handled in the analyses. If applicable, describe which groupings were chosen, and why	8-11
Statistical methods	#12a	Describe all statistical methods, including those used to control for confounding	11-13
Statistical methods	#12b	Describe any methods used to examine subgroups and interactions	12-13
Statistical methods	#12c	Explain how missing data were addressed	
Statistical methods	#12d	If applicable, describe analytical methods taking account of sampling strategy	
Statistical methods	#12e	Describe any sensitivity analyses	
**Results**			
Participants	#13a	Report numbers of individuals at each stage of study—eg numbers potentially eligible, examined for eligibility, confirmed eligible, included in the study, completing follow-up, and analysed. Give information separately for for exposed and unexposed groups if applicable.	13
Participants	#13b	Give reasons for non-participation at each stage	13
Participants	#13c	Consider use of a flow diagram	

For peer review only

Descriptive data	#14a	Give characteristics of study participants (eg demographic, clinical, social) and information on exposures and potential confounders. Give information separately for exposed and unexposed groups if applicable.	
Descriptive data	#14b	Indicate number of participants with missing data for each variable of interest	14
Outcome data	#15	Report numbers of outcome events or summary measures. Give information separately for exposed and unexposed groups if applicable.	14-15
Main results	#16a	Give unadjusted estimates and, if applicable, confounder-adjusted estimates and their precision (eg, 95% confidence interval). Make clear which confounders were adjusted for and why they were included	15-18
Main results	#16b	Report category boundaries when continuous variables were categorized	15-18
Main results	#16c	If relevant, consider translating estimates of relative risk into absolute risk for a meaningful time period	15-18
Other analyses	#17	Report other analyses done—e.g., analyses of subgroups and interactions, and sensitivity analyses	15-18
**Discussion**			
Key results	#18	Summarise key results with reference to study objectives	19-21
Limitations	#19	Discuss limitations of the study, taking into account sources of potential bias or imprecision. Discuss both direction and magnitude of any potential bias.	21
Interpretation	#20	Give a cautious overall interpretation considering objectives, limitations, multiplicity of analyses, results from similar studies, and other relevant evidence.	19-21
Generalisability	#21	Discuss the generalisability (external validity) of the study results	22
**Other Information**			
Funding	#22	Give the source of funding and the role of the funders for the present study and, if applicable, for the original study on which the present article is based	23

None The STROBE checklist is distributed under the terms of the Creative Commons Attribution License CC-BY. This checklist can be completed online using https://www.goodreports.org/, a tool made by the EQUATOR Network in collaboration with Penelope.ai

For peer review only

BMJ Open

Acute psychological impact of Coronavirus Disease 2019 outbreak among psychiatric professionals in China：A multicenter, cross-sectional, web-based study

Journal:	*BMJ Open*
Manuscript ID	bmjopen-2020-047828.R2
Article Type:	Original research
Date Submitted by the Author:	11-Apr-2021
Complete List of Authors:	Guo, Xin; Renmin Hospital of Wuhan University,<br>McCutcheon, Robert; King's College London<br>Pillinger, Toby; King's College London<br>Arumuham, Atheeshaan; King's College London<br>Chen, Jianhua ; Shanghai Clinical Research Center for Mental Health, Shanghai Key Laboratory of Psychotic Disorders, Shanghai Mental Health Center, Shanghai Jiao Tong University School of Medicine<br>Ma, Simeng ; Renmin Hospital of Wuhan University<br>Yang, Jun ; Huazhong University of Science and Technology<br>Wang, Ying ; Renmin Hospital of Wuhan University<br>Hu, Shaohua ; Zhejiang University, Department of Psychiatry<br>Wang, Gaohua ; Renmin Hospital of Wuhan University<br>Liu, Zhong-Chun; Renmin Hospital of Wuhan University, Department of Psychiatry
**Primary Subject Heading**:	Mental health
Secondary Subject Heading:	Mental health
Keywords:	COVID-19, MENTAL HEALTH, PSYCHIATRY

SCHOLARONE™
Manuscripts

I, the Submitting Author has the right to grant and does grant on behalf of all authors of the Work (as defined in the below author licence), an exclusive licence and/or a non-exclusive licence for contributions from authors who are: i) UK Crown employees; ii) where BMJ has agreed a CC-BY licence shall apply, and/or iii) in accordance with the terms applicable for US Federal Government officers or employees acting as part of their official duties; on a worldwide, perpetual, irrevocable, royalty-free basis to BMJ Publishing Group Ltd ("BMJ") its licensees and where the relevant Journal is co-owned by BMJ to the co-owners of the Journal, to publish the Work in this journal and any other BMJ products and to exploit all rights, as set out in our licence.

The Submitting Author accepts and understands that any supply made under these terms is made by BMJ to the Submitting Author unless you are acting as an employee on behalf of your employer or a postgraduate student of an affiliated institution which is paying any applicable article publishing charge ("APC") for Open Access articles. Where the Submitting Author wishes to make the Work available on an Open Access basis (and intends to pay the relevant APC), the terms of reuse of such Open Access shall be governed by a Creative Commons licence – details of these licences and which Creative Commons licence will apply to this Work are set out in our licence referred to above.

Other than as permitted in any relevant BMJ Author's Self Archiving Policies, I confirm this Work has not been accepted for publication elsewhere, is not being considered for publication elsewhere and does not duplicate material already published. I confirm all authors consent to publication of this Work and authorise the granting of this licence.

For peer review only

Word count: 3669

Acute psychological impact of Coronavirus Disease 2019 outbreak among psychiatric professionals in China: A multicenter, cross-sectional, web-based study

Xin Guo[1,2], Robert McCutcheon[2,3], Toby Pillinger[2,3], Atheeshaan Arumuham[2,3], Jianhua Chen[4], Simeng Ma[1], Jun Yang[5], Ying Wang[1], Shaohua Hu[6], Gaohua Wang[1], Zhong-Chun Liu[1]

[1]Department of Psychiatry, RenMin Hospital of Wuhan University, Wuhan, China

[2]Department of Psychosis Studies, Institute of Psychiatry, Psychology & Neuroscience, King's college of London, London, UK.

[3]Psychiatric Imaging Group, MRC London Institute of Medical Sciences, Hammersmith Hospital, London, UK

[4]Shanghai Clinical Research Center for Mental Health, Shanghai Key Laboratory of Psychotic Disorders, Shanghai Mental Health Center, Shanghai Jiao Tong University School of Medicine, Shanghai, China.

[5]Computer Science and Technology, Huazhong University of Science and Technology, Wuhan, China.

[6]Department of Psychiatry, First Affiliated Hospital, Zhejiang University School of

For peer review only - http://bmjopen.bmj.com/site/about/guidelines.xhtml

Medicine, Hangzhou, China

Corresponding author:

Zhong-Chun Liu, PhD

Department of Psychiatry, RenMin Hospital of Wuhan University, Wuhan, China

238 Jiefang Road, Wuchang District, Wuhan, China

Email: zcliu6@whu.edu.cn

For peer review only

For peer review only - http://bmjopen.bmj.com/site/about/guidelines.xhtml

Abstract

Objectives: To assess the magnitude of mental health outcomes and associated factors among psychiatric professionals in mental health services during COVID-19 in China.

Design, Setting and Participants: This cross-sectional, survey-based, region-stratified study collected demographic data and mental health measurements from psychiatric professionals in 34 hospitals from 29 January and 7 February 2020, in China. Hospitals equipped with fever clinics or deployed on wards for patients with COVID-19 were eligible.

Primary Outcome and Measures: The degree of symptoms of depression, anxiety, insomnia, and distress was assessed by the Chinese versions of PHQ-9, GAD-7, ISI and IES-R, respectively. Multivariable logistic regression analysis and Structural Equation Model was performed to identify factors associated with mental health outcomes.

Results: A total of 610 psychiatric professionals were included. 29.8% were employed in Wuhan, and 22.5% were frontline workers. A considerable proportion of participants reported symptom of depression (461 [75.6%]), anxiety (282 [46.2%]), insomnia (336 [55.1%]) and mental stress (481 [78.9%]). Their mental health was primarily affected by worrying about infection (e.g. OR 2.36[95% CI 1.27-4.39] for

For peer review only - http://bmjopen.bmj.com/site/about/guidelines.xhtml

anxiety), risks of exposure to COVID-19 (e.g. having inadequate PPE, OR 2.43[1.32-4.47] for depression), self-perceived physical health (e.g. OR 3.22[2.24-4.64] for mental stress). Information sources of COVID-19 may exacerbate (e.g. Information from relatives, OR 2.16[1.46-3.21] for mental stress) or alleviate (e.g. Information from TV, OR 0.52[0.35-0.77] for mental stress) their mental health. Their mental health would benefit from greater availability of mental health care from MHS (e.g. MHS through psychological support materials OR 0.66[0.43-0.99] for anxiety). The SEM analysis indicated that worrying about infection could be the primary mediator that affects their mental health.

Conclusions: These findings emphasize the importance of being prepared to protect the mental health of psychiatric professionals themselves through mental health interventions at times of widespread crisis.

For peer review only

Strengths and limitations of this study

- Provide a timely comprehensive nationwide evaluation of the acute psychological impact on psychiatric professionals at the early break of COVID-19 in China.
- Possible sampling bias exists because of the voluntary response sampling method.
- This cross-sectional study does not record the mental health status of psychiatric professionals before the outbreak or track the efficacy of psychological services among psychiatric professionals.
- A larger sample size is needed to verify the results.

For peer review only

For peer review only - http://bmjopen.bmj.com/site/about/guidelines.xhtml

Introduction

The 2019 epidemic of novel coronavirus disease (COVID-19) that initiated in Wuhan (Hubei Province, China),[1] is now recognized by the World Health Organization (WHO) as a global public health threat. [2]. In addition to the resulting increased burden on physical healthcare services, there is mounting recognition of the impact that the pandemic may have upon public mental health and therefore mental health services. An effectively functioning mental health service requires sufficient levels of staffing, and staff that are in good physical and mental health themselves.

The mental health of health-care workers in general has been affected during the immediate wake of the COVID-19 epidemic.[3] [4] Psychological distress among medical staff has been noted in the form of increased reported symptoms of anxiety, depression, insomnia and posttraumatic stress symptoms [5] [6]. In addition, workers may also experience isolation from their families or community because of infection transmission risk and stigmatization. [7]

The specific impact of the pandemic on the mental health of psychiatric professionals is less well characterised. In the current study, a multicenter survey was performed to assess the psychological distress in psychiatric professionals in China one week after

For peer review only - http://bmjopen.bmj.com/site/about/guidelines.xhtml

Wuhan city locked down. A brief questionnaire was designed and distributed to psychiatric professionals of several hospitals through WeChat group chat application, including the psychiatric professionals serving in psychiatric hospitals and psychiatric departments of general hospitals in Hubei province (including Wuhan) and other provinces, and also including psychiatric professionals working in fever clinics, infectious disease department and other departments. In addition, we investigated which factors are associated with an increased risk of mental health symptoms among psychiatric professionals, and whether there was any evidence for any potential protective factors.

2. Methods

2.1 Online survey

A WeChat-based online survey was carried out by psychiatric professionals between 29 January and 7 February 2020, the second week after Wuhan city locked down. During the survey period, the cumulative number of confirmed and suspected COVID-19 cases was climbing exponentially (see Fig. 1).

The online survey was conducted with the WeChat-based survey program 'Questionnaire Star' based on voluntary response sampling method. The target population was psychiatric professionals working in hospitals that established fever clinics or wards for patients with COVID-19. Each WeChat account of a participant was

For peer review only - http://bmjopen.bmj.com/site/about/guidelines.xhtml

authenticated by their ID card in China. The online advert with a Quick Response (QR) code directing to the online survey was distributed in psychiatric professionals' WeChat working groups of several provinces. Filling the questionnaires outside of working hours was advised. This study was reviewed and approved by the Clinical Research Ethics Committee of Renmin Hospital of Wuhan University (WDRY2020-K004). All subjects provided informed consent electronically prior to registration. Prior to providing informed consent potential participants were provided with information regarding the research project including its primary goals. After reading this information potential participants were provided with the option to either decline or consent to take part in the study. Only those who consented were taken to the questionnaire page. Respondents could terminate the survey at any time or during any question if they wished to.

2.2 Patient and Public Involvement

No patients were involved in this study.

2.3 Questionnaire

The questionnaire consists of seven sections: basic demographic data, mental health assessment, risks of exposure to COVID-19, source of information about COVID-19, mental healthcare services (MHS) accessed, worry about infection and self-perceived physical health before the COVID-19 outbreak. The questionnaire required 15 minutes to complete.

For peer review only - http://bmjopen.bmj.com/site/about/guidelines.xhtml

2.3.1 Demographic data

Basic demographic data include gender, age, marital status (unmarried/other, married), educational level (high school or lower, undergraduate, postgraduate or higher), location, job type (doctor or nurse), job title (junior, intermediate, or senior), and hospital type (tertiary, secondary or other).

2.3.2 Mental health assessment

We used four well validated scales to assess the mental health status of psychiatric professionals. The 9-item Patient Health Questionnaire (PHQ-9), the 7-item Generalized Anxiety Disorder (GAD-7), the 7-item Insomnia Severity Index (ISI) and the 22-item Impact of Event Scale-Revised (IESR) were used to evaluate depression, anxiety, insomnia and stress, respectively. The PHQ-9 is a self-report measure used to assess the severity of depression and has good reliability and validity in Chinese population (Cronbach's α=0.89), [8] with the total scores ranged from 0-27, and a s score of 10 or greater defined as a high probability of meeting diagnostic criteria for depression. [9]The GAD-7 is a self-rated scale to evaluate the severity of anxiety and has good reliability and validity in Chinese population (Cronbach's α=0.89), [10] the total score of GAD-7 ranged from 0 to 21, and a score of greater than 10 was identified as a high probability of meeting diagnostic criteria for anxiety. [11] The ISI is a measure of insomnia severity that has been shown to be valid and reliable, and a total score of greater than 10 was defined as a high probability of meeting diagnostic criteria for

For peer review only - http://bmjopen.bmj.com/site/about/guidelines.xhtml

insomnia. [12] Post traumatic symptoms response to a specific stressful life event were accessed using the IES-R, which also has extensive reliability and validity (Cronbach's α for 3 subscales were 0.89 (Intrusion), 0.85 (Avoidance), and 0.83 (Hyperarousal), respectively), [13] a score of greater than 34 was identified as mental stress. [14]

2.3.3 Risk of exposure to COVID-19

Exposure to COVID-19 was determined with the following questions asked to psychiatric professionals: Have you been diagnosed with COVID-19? Do you manage patients diagnosed with COVID-19? Has your family been diagnosed with COVID-19? Have your friends been diagnosed? Have your neighbors (people living in the same community who may or may not know each other) been diagnosed? Participants were then asked whether there was anyone living with them with suspected symptoms. In terms of personal protection, participants were asked whether they had enough personal protection equipment (PPE) and enough training for personal protection. The possible responses to each question were yes or no.

2.3.4 Accessed mental health services (MHS)

The following question was used to determine which psychological services the subject had accessed: "Have you ever received the following services: psychological support materials (leaflets, brochures and books provided by mental health workers and distributed to staff in the hospital), psychological resources available through

For peer review only

media (psychological assistance methods and techniques provided by psychologists through social media), or online counseling or psychotherapy (including individual or group counselling, or structural psychotherapy)?".

2.3.5 Source of information about COVID-19

The following question was used to determine from which source the subject had accessed for information regarding COVID-19: "Which way do you used to get information about COVID-19: relatives (information through direct communication with family, neighbors, colleagues, friends or strangers), social media (information from unofficial accounts in WeChat Moments, Weibo, TikTok, and other digital platform), TV (information from national and local official television channels), and other (other information sources of COVID-19) and daily time (hours) spending on information of COVID-19". Then, participants were asked how helpful the information they received were. Answer options included considerably helpful, medium helpful, slightly helpful or not helpful at all.

2.3.6 Self-perceived physical health

Physical health status was determined by asking participants to compare their health status to normal people before the outbreak of COVID-19: How do you perceive your health status compared to normal people before the outbreak? Answer option included strong, normal or weak.

For peer review only - http://bmjopen.bmj.com/site/about/guidelines.xhtml

2.3.7 Worrying about infection

Worry about infection was determined by asking participants if they worry about their vulnerability to infection of COVID-19 in workplace or at home. Possible responses were yes or no.

2.4 Statistical analysis

All statistical analyses were carried out in the statistical programming language R (version-3.6.0). [15]

A bivariate analysis was performed firstly by using Chi-square test to determine the effect of six components (demographic data, risks of exposure to COVID-19, source of information about COVID-19, MHS accessed, worry about infection and self-perceived physical health) on prevalence of four mental health symptoms (depression, anxiety, insomnia and mental stress, assessed by using PHQ-9, GAD-7, ISI, and IES-R separately) among psychiatric professionals.

Stepwise logistic regression analysis according to Akaike information criterion (AIC) was then performed to examine the effects of the six components on four mental health assessment (PHQ-9, GAD-7, ISI, and IES-R scores) separately. The significance of association between the potential risk factors and outcomes were checked by analysis of variance (ANOVA) and prediction accuracy of models were counted by AUC

For peer review only - http://bmjopen.bmj.com/site/about/guidelines.xhtml

(area under ROC curve).

Finally, a structural equation model (SEM) was constructed via R package lavaan 0.6-7 [16] to explore the relationship between mental health assessment (PHQ-9. GAD-7, ISI and IES-R) and the six components, namely demographic data, risks of exposure to COVID-19, source of information about COVID-19, MHS accessed, worry about infection and self-perceived physical health. The estimation method used weighted least squares with mean and variance adjustment test statistics.[17] We used a Monte Carlo method with 1000 guided resamplings to construct a confidence interval for the estimation effect.[18] In SEM, several criteria, such as ratio of value of chi-square test of model fit and degree of freedom (x^2 / df) <3, root mean square error of approximation (RMSEA) values<0.08 and goodness-to-fit index (GFI) values>0.90, indicate acceptable models (Hu and Bentler, 1998).

P values < 0.05 indicated that a difference was statistically significant.

3. Results

Initially, 834 completed questionnaires were received, with a response rate of 78.5%. The duration (seconds) to complete the questionnaire was recorded by Questionnaire star in WeChat. We excluded 119 questionnaires with a complete duration of fewer than 5 minutes (300 seconds) or more than 20 minutes (1,200 seconds), to ensure that

For peer review only - http://bmjopen.bmj.com/site/about/guidelines.xhtml

all questions had been well understood and completed consecutively by respondents. We further excluded 105 questionnaires from provinces that had recruited less than 20 participants. In total, 224 questionnaires were excluded, leaving 610 questionnaires for analysis. Respondents completing the included questionnaires and excluded questionnaires were comparable in age and sex.

3.1 Demographic characteristics

In total, 610 psychiatric professionals, including 164 (26.9%) doctors and 446 (73.1%) nurses were included into analysis. 29.8% of these participants were employed in Wuhan, and 53.3% were employed in Hubei provinces other than Wuhan. 22.5% were frontline mental health staff. 53.3% of participants were working in a tertiary hospital. The participants tended to be female (76.1%), aged 26-40 years (63.1%), married (68.2%), with an educational level of undergraduate (91.3%), and have a junior job title (62.1%), as shown in Table 1.

3.2 Mental health assessment

According to standard questionnaire cut-offs of the PHQ-9, GAD-7, ISI, IES-R scores, 461 (75.6%) of individuals met criteria for depression, 282 (46.2%) for anxiety, 336 (55.1%) for insomnia, and 481 (78.9%) for high levels of mental stress.

3.3 Assessed mental health services (MHS)

36.3% of participants had received psychological support materials, 50.4% had

For peer review only

obtained psychological resources available through media, and 17.5% had participated in online psychological counseling/psychotherapy, as shown in Table 1.

3.3 Risk factors & protective factors of psychological impact of COVID-19 on psychiatric professionals

Bivariate Analysis

We found that psychiatric professionals who took part in frontline work presented higher prevalence of all four mental health symptoms (89.9% vs 71.5%, P<0.001 for depression; 66.4% vs 40.4%, P<0.001 for anxiety; 67.9% vs 51.4%, P<0.001 for insomnia; 69.3% vs 52.0%, P<0.001 for mental stress) compared with those who worked at secondary line. Treating and diagnosing infected patient (95% vs 72.6%, P<0.001 for depression; 75% vs 41.9%, P<0.001 for anxiety; 75% vs 52.1%, P<0.001 for insomnia; 82.5% vs 51.9%, P<0.001 for mental stress), working with infected colleague (87.9% vs 71.9%, P<0.001 for depression; 67.9% vs 39.8%, P<0.001 for anxiety; 66% vs 52%, P<0.001 for insomnia; 72.1% vs 51.1%, P<0.001 for mental stress), employed in Wuhan (Wuhan vs Hubei except Wuhan vs Other provinces except Hubei: 89% vs 72.6% vs 61.2%, P<0.001 for depression; 65.4% vs 39.1% vs 35%, P<0.001 for anxiety; 70% vs 49% vs 48.5%, P<0.001 for insomnia; 72.5% vs 49.5% vs 46.6, P<0.001 for mental stress), worse self-perceived physical health (strong vs normal vs weak: 60.7% vs 91.5% vs 100%, P<0.001 for depression; 26.4% vs 67.1% vs 88.9%, P<0.001 for anxiety; 34.6% vs 76.7% vs 100%, P<0.001 for insomnia; 39.6% vs 73.1% vs 88.9, P<0.001 for mental

For peer review only - http://bmjopen.bmj.com/site/about/guidelines.xhtml

stress), worrying about infection (83.5% vs 46.2%, P<0.001 for depression; 54.2% vs 16.9%, P<0.001 for anxiety; 60.6% vs 34.6%, P<0.001 for insomnia; 63.5% vs 27.7%, P<0.001 for mental stress) and obtaining information about COVID-19 from relatives (79.4% vs 71.1%, P=0.02 for depression; 50.9% vs 40.8%, P=0.02 for anxiety; 59% vs 50%, P=0.03 for insomnia; 64.4% vs 46.1%, P<0.001 for mental stress) were also significantly associated with higher prevalence of all four mental health symptoms. Self-infection (100% vs 45.8%, p=0.049) and living with infected neighbor (65.4% vs 39.1, p=0.002) were significantly associated with higher prevalence of anxiety. MHS accessed through psychological support materials (70.7% vs 79.9%, P=0.01 for depression; 38.7% vs 52.9%, P<0.001 for anxiety; 49% vs 61%, P=0.004 for insomnia; 51.6% vs 59.8%, P=0.05 for mental stress), MHS accessed through media (71.5% vs 81.3%, P=0.007 for depression; 40.8% vs 54%, P=0.002 for anxiety), and obtaining information about COVID-19 from TV (70.3% vs 81.9%, P=0.001 for depression; 41.7% vs 51.6%, P=0.02 for anxiety; 51% vs 60%, P=0.02 for insomnia; 50.5% vs 62.5%, P=0.004 for mental stress) were significantly associated with lower prevalence of mental health symptoms, as shown in Table 2.

Logistic Regression

To explore the potential risk and protective factors of mental health of psychiatric professionals, stepwise logistic regression analyses on PHQ-9, GAD-7, ISI and IES-R scores separately was performed. After controlling for potential confounding

For peer review only

variables, checking the significance of effect of each variable using ANOVA, we identified risk and protective factors as follows (Table 3):

PHQ-9

Treating and diagnosing infected patient (odds ratio [OR], 2.77; 95% CI, 1.05-7.27; P = .039), worrying about infection (OR, 0.33; 95% CI, 0.21-0.52; p<0.001), having inadequate PPE (OR, 2.43; 95% CI, 1.32-4.47; p=0.005), and worse self-perceived physical health (OR, 2.76; 95% CI, 1.73-4.41; p<0.001) were significantly associated with higher PHQ-9 score among psychiatric professionals. Obtaining information about COVID-19 from relatives (OR, 1.64; 95% CI, 1.03-2.61; p=0.036) was also significantly associated with increased PHQ-9 score, while obtaining information from TV (OR, 0.57; 95% CI, 0.36-0.91; p=0.02) was significantly associated with lower PHQ-9 score.

GAD-7

Doctors were found to have higher GAD-7 scores (OR, 0.55; 95% CI, 0.34-0.91; p=0.019) compared with nurses. Self-infection seems a primary risk factor to anxiety (OR, 2530278.72; 95% CI, 0-Inf; p=0.045), and all self-infection psychiatric professionals met criteria of severe anxiety. Treating and diagnosing infected patient (OR, 2.36; 95% CI, 1.27-4.39; p=0.007), working with infected colleague (OR, 2.17; 95% CI, 1.31-3.61; p=0.003), living with infected neighbor (OR, 2.13; 95% CI, 1.14-3.96; p=0.018), having inadequate PPE (OR, 1.7; 95% CI, 1.09-2.65; p=0.02), worrying

For peer review only - http://bmjopen.bmj.com/site/about/guidelines.xhtml

about infection (OR, 0.25; 95% CI, 0.14-0.44; p<0.001) and obtaining information from other methods (OR, 2.36; 95% CI, 1.29-4.32; p=0.005) were associated with increased GAD-7 score. MHS accessed through psychological support materials (OR, 0.66; 95% CI, 0.43-0.99; p<0.001) and those who thought information about COVID-19 was helpful (OR, 1.17; 95% CI, 1.01-1.36; p=0.35) showed lower GAD-7 score.

ISI

Treating and diagnosing infected patients (OR, 1.91; 95% CI, 1.06-3.46; p=0.032), worse physical health status (OR, 5.68; 95% CI, 3.92-8.25; p<0.001) and those worrying about infection (OR, 0.58; 95% CI, 0.36-0.92; p=0.02) were significantly associated with higher ISI score.

IES-R

Treating and diagnosing infected patient (OR, 2.97; 95% CI, 1.54-5.73; p=0.001), worse self-perceived physical health (OR, 3.22; 95% CI, 2.24-4.64; p<0.001) and worrying about infection (OR, 0.35; 95% CI, 0.22-0.56; p<0.001) were significantly associated with higher IES-R score among psychiatric professionals. Psychiatric professionals obtaining information from relatives showed significantly higher IES-R score (OR, 2.16; 95% CI, 1.46-3.21; p<0.001), while those who obtained information from TV (OR, 0.52; 95% CI, 0.35-0.77; p=0.001), and those who thought information about COVID-19 was helpful (OR, 0.52; 95% CI, 0.35-0.77; p=0.001) showed lower IES-R score.

For peer review only - http://bmjopen.bmj.com/site/about/guidelines.xhtml

For peer review only

Structural Equation Modelling

Finally, we established an SEM of the association between the seven areas described in methods. The chi-square test of model fit yielded a value of 729.6, with degrees of freedom = 253, P value= 0.000, x^2 / df = 2.88, RMSEA = 0.056, GFI = 0.909, and TLI = 0.973, indicating a good fit. The results showed that gender, the risk factors of exposure, source of information about COVID-19 and self-perceived physical health status affected mental health through worry about infection, indicated that worrying about infection could be the primary mediator that affects the mental health of psychiatric professionals during the COVID-19 outbreak. Mental health services alleviated mental health symptoms through decreasing the worry about infection. The results are shown in Figure 2.

4. Discussion

The present cross-section study firstly investigated the psychological impact of COVID-19 among psychiatric professionals in China during the early outbreak of the pandemic. The overall prevalence of depression, anxiety, insomnia and stress in psychiatric in the second week after Wuhan city locked down were 75.6%, 46.2%, 55.1%, and 78.9%, respectively. In comparison with recent studies of general health care workers [6, 19, 20], the psychological impact of COVID-19 outbreak seem to be more severe among psychiatric professionals compared with general health care workers (the overall

For peer review only - http://bmjopen.bmj.com/site/about/guidelines.xhtml

prevalence of depression, anxiety, insomnia and stress was estimated at 13.9%-46.9%, 16.1%-41.1%, 19.7% and 9.8%-69.1%, respectively). Firstly, most of the participants in the present study were from Wuhan and other cities in Hubei province, according to these recent studies, the prevalence of depression, anxiety and stress among health care workers working in Hubei province (including Wuhan) were estimated at 82.9%-88.7%, 66.9%-80.4%, and 87.6%-92.9%, respectively. Secondly, patients with mental health disorders were believed more susceptible to infections for several reasons, including cognitive impairment, little awareness of risk, and diminished efforts regarding personal protection in patients, also the confined conditions in psychiatric hospital and psychiatric department in general hospital. [21] Our research also determined that the risk of exposure to COVID-19, treating and diagnosing infected patient were significantly associated with higher risk of mental health symptoms among psychiatric professionals. Thirdly, the psychological impact of COVID-19 in different pandemic stages were different. [19, 20] Our study was performed in the early wake of the outbreak and finished by the end of the second week after Wuhan city locked down, the rapid rising number of identified and suspected cases and unprecedented scale of the city locked down caused public panic and psychological stress. [3] This could exacerbate the mental health problems among health care workers including psychiatric professionals. Furthermore, our study found that females had a higher risk of developing depression symptoms but not anxiety, insomnia or mental stress symptoms during the COVID-19 pandemic among psychiatric professionals. A recent meta-analysis study showed that females had a

For peer review only - http://bmjopen.bmj.com/site/about/guidelines.xhtml

higher risk of developing psychiatric symptoms and reported lower psychological wellbeing during the COVID-19 pandemic, but the findings of the studies included in the published meta-analysis were not consistent.[22] This may due to various influences such as preconception and fertility issues, pregnancy, postpartum, miscarriage and intimate partner violence.[23] Further studies will be needed to clarify this.

This is the first study to explore the psychological effect of different source of information about COVID-19 on mental health among psychiatric professionals. At the early stage of the outbreak, the unpredictable future of the pandemic was worsened by myths and misinformation, often driven by erroneous news reports from unofficial information sources and the public's misunderstanding of health messages, thus causing worry and increasing the risk of mental health problems in population. [3] The WHO uses the term "infodemic" to describe the impossible amounts of misinformation and disinformation in worldwide, and the doctors and scientists have long been called on to share their wisdom through the official media. [24] [25] Our study determined that information from social media, direct communication with relatives, and other unofficial information sources increased risk of mental health symptoms among psychiatric professionals. The information of COVID-19 from official source such as TV seems a protective factor for mental health among psychiatric professionals, thus indicating that trustable sources of reliable and accurate information are not just helpful for epidemic control, but also good to mental health.

For peer review only - http://bmjopen.bmj.com/site/about/guidelines.xhtml

To efficiently cope with psychological impact of the COVID-19 outbreak, the Chinese Government has implemented rapid and comprehensive psychological crisis intervention, including publishing psychological support materials, such as leaflets and books, establishing psychological intervention teams on site [4] , mental health service online through social media platform, and also telephone guidance to help address mental health problems.[26] Our study found that psychiatric professionals had benefited from availability of mental health care from public mental health services (psychological materials and psychological help from media) and personalized mental health services (online counseling and psychotherapy).

Our study also has some limitations. First, the study is cross-sectional and does not record the mental health status before the outbreak, or track the efficacy of psychological services among psychiatric professionals. Second, most respondents (76.1%) in this study were made up by female, this may due to the overall psychiatric workforce was predominantly nurses (73.1%), and most nurses were female in China. As by 2019, the ratio of Chinese psychiatric doctors to nurses was close to 1:2.1, [27] the ratio of doctors to nurses in this study is 1:2.7. While a response bias cannot be ruled out the available data regarding the demographics of the workforce suggests, that at least as regards occupation and gender our sample is not substantially different from the overall workforce. Third, a larger sample size is needed to verify the results.

For peer review only - http://bmjopen.bmj.com/site/about/guidelines.xhtml

In summary, the results demonstrate that a strikingly large portion of psychiatric professionals in China suffered from mental health disturbances during the early break of COVID-19. Their mental health was primarily affected by worry about being infected, risks of exposure to COVID-19, self-perceived physical health. Unreliable information sources of COVID-19 may exacerbate their mental health symtoms, and their mental health would benefit from greater availability of mental health care from public and personalized mental health services. Among the steps needed to better prepare for future infectious disease outbreaks would be a greater investment in the infectious diseases' prevention and control in psychiatric hospitals and psychiatric department in general hospitals, and in the mental health services among psychiatric professionals' themselves to protect and care for future psychiatric professionals who find themselves unexpectedly on the dangerous front lines of disease response.

Acknowledgments

Author Contribution: Dr Guo, Dr McCutcheon and Prof Liu had full access to all the data in the study and takes responsibility for the integrity of the data and the accuracy of the data analysis.

Design and conduct of the study: Dr Guo, Dr McCutcheon, Prof Liu.

Collection, management, analysis, and interpretation of the data: Dr Guo, Dr McCutcheon, Dr Pillinger, Dr Arumuham, Dr Chen, Dr Ma, Dr Yang, Dr Wang, Dr Hu.

Preparation, review, or approval of the manuscript: Dr Guo, Dr McCutcheon, Dr

For peer review only - http://bmjopen.bmj.com/site/about/guidelines.xhtml

Arumuham, Dr Chen, Prof Liu and Prof Wang.

Decision to submit the manuscript for publication: Prof Liu and Prof Wang.

Funding/Support: XG's work is funded by the National Key R&D Program of China (no. 2018YFC1314600), and fellowship grants from Chinese Scholarship Council. ZCL's work is funded by the National Key R&D Program of China (no. 2018YFC1314600). RM's research is funded by the Wellcome Trust (no. 200102/Z/15/Z) and a NIHR clinical lectureship.

Ethical Approval Statement

This study was reviewed and approved by the Clinical Research Ethics Committee of Renmin Hospital of Wuhan University (WDRY2020-K004).

Data availability statement

All data relevant to the study are included in the article.

Declaration of interest:

The authors declare that they have no conflict of interest.

References

1 Chan JF, Yuan S, Kok KH, et al. A familial cluster of pneumonia associated with the 2019 novel coronavirus indicating person-to-person transmission: a study of a family cluster. *Lancet*

For peer review only - http://bmjopen.bmj.com/site/about/guidelines.xhtml

2020;395(1474-547X (Electronic)):514-23.

2 WHO. Statement on the second meeting of the International Health Regulations (2005) Emergency Committee regarding the outbreak of novel coronavirus (2019-nCoV). https://www.who.int/news-room/detail/30-01-2020-statement-on-thesecond-meeting-of-the-international-health-regulations-(2005)-emergency-committee-regarding-the-outbreak-of-novel-coronavirus-(2019-ncov) (assessed Feb 15, 2020). 2020

3 Bao Y, Sun Y, Meng S, et al. 2019-nCoV epidemic: address mental health care to empower society. *Lancet* 2020;395(1474-547X (Electronic)):e37-e38.

4 Kang L, Li Y, Hu S, et al. The mental health of medical workers in Wuhan, China dealing with the 2019 novel coronavirus. *Lancet Psychiatry* 2020(2215-0374 (Electronic)) doi: https://doi.org/10.1016/s2215-0366(20)30047-x

5 da Silva FCT, Neto MLR. Psychiatric symptomatology associated with depression, anxiety, distress, and insomnia in health professionals working in patients affected by COVID-19: A systematic review with meta-analysis. *Prog Neuropsychopharmacol Biol Psychiatry* 2020(1878-4216 (Electronic)) doi: 10.1016/j.pnpbp.2020.110057

6 Lin K, Yang BX, Luo D, et al. The Mental Health Effects of COVID-19 on Health Care Providers in China. *American Journal of Psyhciatry* 2020(1535-7228 (Electronic))

7 Van Bortel T, Basnayake A, Wurie F, et al. Psychosocial effects of an Ebola outbreak at individual, community and international levels. *Bull World Health Organ* 2016;94(1564-0604 (Electronic)):210-14.

8 Chen S, Fang Y, Chiu H, et al. Validation of the nine-item Patient Health Questionnaire to screen for major depression in a Chinese primary care population. *Asia Pac Psychiatry* 2013;5(1758-5872 (Electronic)):61-68.

9 Manea L, Gilbody S, McMillan D. Optimal cut-off score for diagnosing depression with the Patient Health Questionnaire (PHQ-9): a meta-analysis. *Canadian Medical Association Journal* 2012;184(1488-2329 (Electronic)):e191-e96.

10 Tong X, An D, McGonigal A, et al. Validation of the Generalized Anxiety Disorder-7 (GAD-7) among Chinese people with epilepsy. *Epilepsy Res* 2015(1872-6844 (Electronic))

11 Spitzer R, Kroenke K, Williams J, et al. A brief measure for assessing generalized anxiety disorder: the GAD-7. *Arch Intern Med* 2006;166(0003-9926 (Print)):1092-97.

12 Morin C, Belleville G, Bélanger L, et al. The Insomnia Severity Index: psychometric indicators to detect insomnia cases and evaluate treatment response. *Sleep* 2011;34(1550-9109 (Electronic)):601-08.

13 Wu K, Chan K. The development of the Chinese version of Impact of Event Scale--Revised (CIES-R). *Soc Psychiatry Psychiatr Epidemiol* 2003;38(0933-7954 (Print)):94-98.

14 Morina N, Ehring T, Priebe S. Diagnostic utility of the impact of event scale-revised in two samples of survivors of war. *PLoS One* 2013;31(1932-6203 (Electronic)):e83916.

15 Team RC. R: A Language and Environment for Statistical Computing. 2013

16 Rosseel Y. Lavaan: A R package for structural equation modeling. *Journal of Statistical Software* 2012;48(2):1-36.

17 DiStefano C, Morgan GB. A Comparison of Diagonal Weighted Least Squares Robust Estimation Techniques for Ordinal Data. *Structural Equation Modeling: A Multidisciplinary Journal* 2014;21(3):425-38. doi: 10.1080/10705511.2014.915373

18 Bauer DJ, Preacher KJ, Gil KM. Conceptualizing and testing random indirect effects and moderated

For peer review only

mediation in multilevel models: new procedures and recommendations. *Psychol Methods* 2006;11(2):142-63. doi: 10.1037/1082-989x.11.2.142 [published Online First: 2006/06/21]

19 Wang Y, Ma S, Yang C, et al. Acute psychological effects of Coronavirus Disease 2019 outbreak among healthcare workers in China: a cross-sectional study. *Transl Psychiatry* 2020;10(2158-3188 (Electronic)):348.

20 Wang L, Zhang M, Liu G, et al. Psychological impact of coronavirus disease (2019) (COVID-19) epidemic on medical staff in different posts in China: A multicenter study. *Journal of Psychiatric Research* 2020;129(1879-1379 (Electronic)):198-205.

21 Yao H, Chen JH, Xu YF. Patients with mental health disorders in the COVID-19 epidemic. *Lancet Psychiatry* 2020;7(2215-0374 (Electronic)):e21.

22 Vindegaard N, Benros ME. COVID-19 pandemic and mental health consequences: Systematic review of the current evidence. *Brain, Behavior, and Immunity* 2020;89(1090-2139 (Electronic)):531-42.

23 Almeida MA-O, Shrestha AD, Stojanac D, et al. The impact of the COVID-19 pandemic on women's mental health. *Archives of Women's Mental Health* 2020(1435-1102 (Electronic))

24 Cinelli M, Quattrociocchi W, Galeazzi A, et al. The COVID-19 social media infodemic. *Sci Rep* 2020(2045-2322 (Electronic)) doi: 10.1038/s41598-020-73510-5

25 Diseases TLI. The COVID-19 infodemic. *Lancet* 2020;20(8):875.

26 Liu S, Yang L, Zhang C, et al. Online mental health services in China during the COVID-19 outbreak. *Lancet Psychiatry* 2020(2215-0374 (Electronic)) doi: 10.1016/S2215-0366(20)30077-8

27 Lin L. The retrospect and prospect of psychiatry in China. *Journal of Peking University (Health Sciences)* 2019;51(3):379-83. doi: 10.19723/j.issn.1671-167X.2019.03.001

For peer review only

For peer review only - http://bmjopen.bmj.com/site/about/guidelines.xhtml

Figure & Table Legend

Figure 1. Timeline of COVID-19 and survey data. Wuhan city was locked down since 23 Jan 2020, and the online survey was conducted 1 week after that. The cumulative number seemed to have entered the rapid rise phase.

------------------------------ Figure 1----------------------------

Figure 2. Direct and indirect effects in SEM. The solid line represents a significant relationship between the two, while the dotted line represents the relationship is not significant.

------------------------------ Figure 2---------------------------

For peer review only - http://bmjopen.bmj.com/site/about/guidelines.xhtml

Table 1. Demographic characteristics and accessed mental health services

Variables		Number	Percentage (%)
Total		610	100
Gender	Female	464	76.1
	Male	146	23.9
Age (years)	18-25	92	15.1
	26-30	198	32.5
	31-40	187	30.7
	>40	133	21.8
Marital status	Unmarried/Other	194	31.8
	Married	416	68.2
Education level	High school or less	18	3.0
	Undergraduate	557	91.3
	Graduate or higher	35	5.7
Location	Wuhan	182	29.8
	Hubei except Wuhan	325	53.3
	Guangdong	50	8.2
	Heibei	25	4.1
	Yunan	28	4.6
Job type	Doctor	164	26.9
	Nurse	446	73.1
Job title	Junior	379	62.1

For peer review only - http://bmjopen.bmj.com/site/about/guidelines.xhtml

	Intermediate	158	25.9
	Senior	73	12.0
Hospital type	tertiary	325	53.3
	Secondary or other	285	46.7
Working position	Front line	137	22.5
	Secondary line	473	77.5
Assessed mental health services			
psychological support materials	Yes	287	47.0
	No	323	53.0
MHS available through media	Yes	358	58.7
	No	252	41.3
counseling or psychotherapy	Yes	82	13.4
	No	528	86.6

1

Table 2. Prevalence of acute psychological effects among psychiatric professionals

Variables	Total	PHQ-9			GAD-7			ISI			IES-R		
		No	Yes	p-value	No	Yes	p-value	No	Yes	p-value	No	Yes	p-value
Total	610												
Gender				0.002			0.09			0.26			0.08
Male	146	50(34.2)	96(65.8)		88(60.3)	58(39.7)		72(49.3)	74(50.7)		74(50.7)	72(49.3)	
Female	464	99(21.3)	365(78.7)		240(51.7)	224(48.3)		202(43.5)	262(56.5)		195(42.0)	269(58.0)	
Physical health				<0.001			<0.001			<0.001			<0.001
Strong	318	125(39.3)	193(60.7)		234(73.6)	84(26.4)		208(65.4)	110(34.6)		192(60.4)	126(39.6)	

For peer review only - http://bmjopen.bmj.com/site/about/guidelines.xhtml

For peer review only

Normal	283	24(8.5)	259(91.5)		93(32.9)	190(67.1)		66(23.3)	217(76.7)		76(26.9)	207(73.1)	
Weak	9	0(0)	9(100)		1(11.1)	8(88.9)		0(0)	9(100)		1(11.1)	8(88.9)	
Worrying about infection				<0.001			<0.001			<0.001			<0.001
Yes	480	79(16.5)	401(83.5)		220(45.8)	260(54.2)		189(39.4)	291(60.6)		175(36.5)	305(63.5)	
No	130	70(53.8)	60(46.2)		108(83.1)	22(16.9)		85(65.4)	45(34.6)		94(72.3)	36(27.7)	
Work position				<0.001			<0.001			<0.001			<0.001
Frontline	137	14(10.2)	123(89.8)		46(33.6)	91(66.4)		44(32.1)	93(67.9)		42(30.7)	95(69.3)	
Secondary line	473	135(28.5)	338(71.5)		282(59.6)	191(40.4)		230(48.6)	243(51.4)		227(48)	246(52)	
Self-infection				0.45			0.049			0.12			0.12
No	605	149(24.6)	456(75.4)		328(54.2)	277(45.8)		274(45.3)	331(54.7)		269(44.5)	336(55.5)	
Yes	5	0(0)	5(100)		0(0)	5(100)		0(0)	5(100)		0(0)	5(100)	
Patient infection				<0.001			<0.001			<0.001			<0.001
No	530	145(27.4)	385(72.6)		308(58.1)	222(41.9)		254(47.9)	276(52.1)		255(48.1)	275(51.9)	
Yes	80	4(5)	76(95)		20(25)	60(75)		20(25)	60(75)		14(17.5)	66(82.5)	
Family member infection				0.28			0.84			0.21			0.65
No	603	149(24.7)	454(75.3)		325(53.9)	278(46.1)		273(45.3)	330(54.7)		267(44.3)	336(55.7)	
Yes	7	0(0)	7(100)		3(42.9)	4(57.1)		1(14.3)	6(85.7)		2(28.6)	5(71.4)	
Colleague infection				<0.001			<0.001			0.003			<0.001
No	470	132(28.1)	338(71.9)		283(60.2)	187(39.8)		227(48)	243(52)		230(48.9)	240(51.1)	
Yes	140	17(12.1)	123(87.9)		45(32.1)	95(67.9)		47(34)	93(66)		39(27.9)	101(72.1)	
Friend infection				0.44			0.14			0.18			0.22
No	571	142(24.9)	429(75.1)		312(54.6)	259(45.4)		261(46)	310(54)		256(44.8)	315(55.2)	
Yes	39	7(17.9)	32(82.1)		16(41)	23(59)		13(33)	26(67)		13(33)	26(67)	

For peer review only

For peer review only - http://bmjopen.bmj.com/site/about/guidelines.xhtml

Neighbour infection				0.13			0.002			0.61			0.27
No	533	136(25.5)	397(74.5)		300(56.3)	233(43.7)		242(45)	291(55)		240(45)	293(55)	
Yes	77	13(16.8)	64(83.1)		28(36.4)	49(63.6)		32(42)	45(58)		29(37.7)	48(62.3)	
Location				<0.001			<0.001			<0.001			<0.001
Wuhan	182	20(11)	162(89)		63(34.6)	119(65.4)		54(30)	128(70)		50(27.5)	132(72.5)	
Hubei except Wuhan	325	89(27.4)	236(72.6)		198(60.9)	127(39.1)		167(51)	158(49)		164(50.5)	161(49.5)	
Other provinces except Hubei	103	40(38.8)	63(61.2)		67(65)	36(35)		53(51.5)	50(48.5)		55(53.4)	48(46.6)	
MHS psychological materials				0.01			<0.001			0.004			0.05
No	323	65(20.1)	258(79.9)		152(47.1)	171(52.9)		127(39)	196(61)		130(40.2)	193(59.8)	
Yes	287	84(29.3)	203(70.7)		176(61.3)	111(38.7)		147(51)	140(49)		139(48.4)	148(51.6)	
MHS available through media				0.007			0.002			0.11			0.15
No	252	47(18.7)	205(81.3)		116(46)	136(54)		103(41)	149(59)		102(40.5)	150(59.5)	
Yes	358	102(28.5)	256(71.5)		212(59.2)	146(40.8)		171(48)	187(52)		167(46.6)	191(53.4)	
Information from relatives				0.02			0.02			0.03			<0.001
No	284	82(28.9)	202(71.1)		168(59.2)	116(40.8)		141(50)	143(50)		153(53.9)	131(46.1)	
Yes	326	67(20.6)	259(79.4)		160(49.1)	166(50.9)		133(41)	193(59)		116(35.6)	210(64.4)	
Information from TV				0.001			0.02			0.02			0.004
No	277	50(18.1)	227(81.9)		134(48.4)	143(51.6)		110(40)	167(60)		104(37.5)	173(62.5)	
Yes	333	99(29.7)	234(70.3)		194(58.3)	139(41.7)		164(49)	169(51)		165(49.5)	168(50.5)	

For peer review only - http://bmjopen.bmj.com/site/about/guidelines.xhtml

Table 3. Risk & protective factors of mental health of psychiatric professionals identified by stepwise logistic regression

Variables	crude OR (95%CI)	crude P value	adj. OR (95%CI)	P (Wald's test)	P(LR-test)
**PHQ-9**					
gender: male vs female	0.6 (0.39,0.92)	0.019	0.63 (0.38,1.03)	0.063	0.066
Physical health (cont. var.)	3.9 (2.51,6.06)	< 0.001	2.76 (1.73,4.41)	< 0.001	< 0.001
Patient infection: yes vs no	4.58 (1.81,11.58)	0.001	2.77 (1.05,7.27)	0.039	0.022
MHS media: yes vs no	0.65 (0.43,0.98)	0.039	0.69 (0.43,1.09)	0.112	0.11
MHS psychotherapy: no vs yes	3.84 (0.5,29.46)	0.196	4.05 (0.49,33.61)	0.195	0.127
Having enough personal protection (PP): no vs yes	3.15 (1.78,5.59)	< 0.001	2.43 (1.32,4.47)	0.005	0.003
Worrying about infection: no vs yes	0.21 (0.13,0.32)	< 0.001	0.33 (0.21,0.52)	< 0.001	< 0.001
Information from relatives: yes vs no	1.81 (1.22,2.68)	0.003	1.64 (1.03,2.61)	0.036	0.035
Information from TV: yes vs no	0.63 (0.42,0.93)	0.022	0.57 (0.36,0.91)	0.02	0.018
**GAD-7**					
gender: male vs female	0.71 (0.48,1.03)	0.071	0.62 (0.37,1.03)	0.066	0.064
Physical health (cont. var.)	5.62 (3.99,7.92)	< 0.001	4.96 (3.37,7.29)	< 0.001	< 0.001
Job type: doctor vs nurse	0.84 (0.59,1.2)	0.343	0.55 (0.34,0.91)	0.019	0.018
Self-infection: yes vs no	2508169.79 (0,Inf)	0.97	2530278.72 (0,Inf)	0.979	0.045
Patient infection: yes vs no	4.16 (2.44,7.1)	< 0.001	2.36 (1.27,4.39)	0.007	0.006
Colleague infection: yes vs no	3.19 (2.14,4.77)	< 0.001	2.17 (1.31,3.61)	0.003	0.002
Friend infection: yes vs no	1.73 (0.9,3.35)	0.102	0.48 (0.2,1.11)	0.086	0.087
Neighbour infection: yes vs no	2.25 (1.37,3.7)	0.001	2.13 (1.14,3.96)	0.018	0.016
MHS psychological support material: yes vs no	0.56 (0.41,0.77)	< 0.001	0.66 (0.43,0.99)	0.045	0.045
Having enough personal protection (PP): no vs yes	2.22 (1.53,3.23)	< 0.001	1.7 (1.09,2.65)	0.02	0.02
Worryinng about infection: no vs yes	0.17 (0.11,0.28)	< 0.001	0.25 (0.14,0.44)	< 0.001	< 0.001

For peer review only - http://bmjopen.bmj.com/site/about/guidelines.xhtml

Information from TV: yes vs no	0.67 (0.49,0.93)	0.015	0.73 (0.49,1.1)	0.131	0.131
Information from other methods: yes vs no	1.46 (0.91,2.35)	0.119	2.36 (1.29,4.32)	0.005	0.005
Thinking Information helpful? (cont. var.)	1.33 (1.18,1.49)	< 0.001	1.17 (1.01,1.36)	0.035	0.035
**ISI**					
gender: male vs female	0.79 (0.55,1.15)	0.221	0.97 (0.62,1.5)	0.88	0.88
education (cont. var.)	1.44 (0.82,2.5)	0.201	1.76 (0.95,3.27)	0.073	0.07
Physical health (cont. var.)	6.35 (4.46,9.06)	< 0.001	5.68 (3.92,8.25)	< 0.001	< 0.001
Patient infection: yes vs no	2.76 (1.62,4.71)	< 0.001	1.91 (1.06,3.46)	0.032	0.027
MHS psychological support material: yes vs no	0.62 (0.45,0.85)	0.003	0.74 (0.51,1.07)	0.111	0.111
MHS psychotherapy: no vs yes	1.65 (0.56,4.89)	0.366	2.63 (0.8,8.63)	0.112	0.103
Having enough training: no vs yes	1.95 (1.37,2.79)	< 0.001	1.45 (0.95,2.21)	0.088	0.087
Having enough personal protection (PP): no vs yes	1.99 (1.36,2.91)	< 0.001	1.4 (0.9,2.2)	0.135	0.134
Worrying about infection: no vs yes	0.34 (0.23,0.52)	< 0.001	0.58 (0.36,0.92)	0.020	0.019
Information from social media: yes vs no	2.08 (0.75,5.81)	0.16	2.76 (0.81,9.4)	0.103	0.093
Information from TV: yes vs no	0.68 (0.49,0.94)	0.019	0.7 (0.49,1.02)	0.067	0.066
**IES-R**					
education (cont. var.)	1.54 (0.88,2.7)	0.129	1.58 (0.84,2.97)	0.154	0.15
gender: male vs female	0.71 (0.49,1.02)	0.067	0.81 (0.52,1.25)	0.34	0.341
Physical health (cont. var.)	4.1 (2.93,5.74)	< 0.001	3.22 (2.24,4.64)	< 0.001	< 0.001
Patient infection: yes vs no	4.37 (2.4,7.98)	< 0.001	2.97 (1.54,5.73)	0.001	< 0.001
Having enough training: no vs yes	1.92 (1.35,2.75)	< 0.001	1.42 (0.95,2.14)	0.089	0.089
Worrying about infection: no vs yes	0.22 (0.14,0.34)	< 0.001	0.35 (0.22,0.56)	< 0.001	< 0.001
Information from relatives: yes vs no	2.11 (1.53,2.93)	< 0.001	2.16 (1.46,3.21)	< 0.001	< 0.001
Information from TV: yes vs no	0.61 (0.44,0.85)	0.003	0.52 (0.35,0.77)	0.001	< 0.001

For peer review only

Think information helpful? (cont. var.)	1.24 (1.1,1.39)	< 0.001	1.15 (1,1.31)	0.045	0.044

1

For peer review only

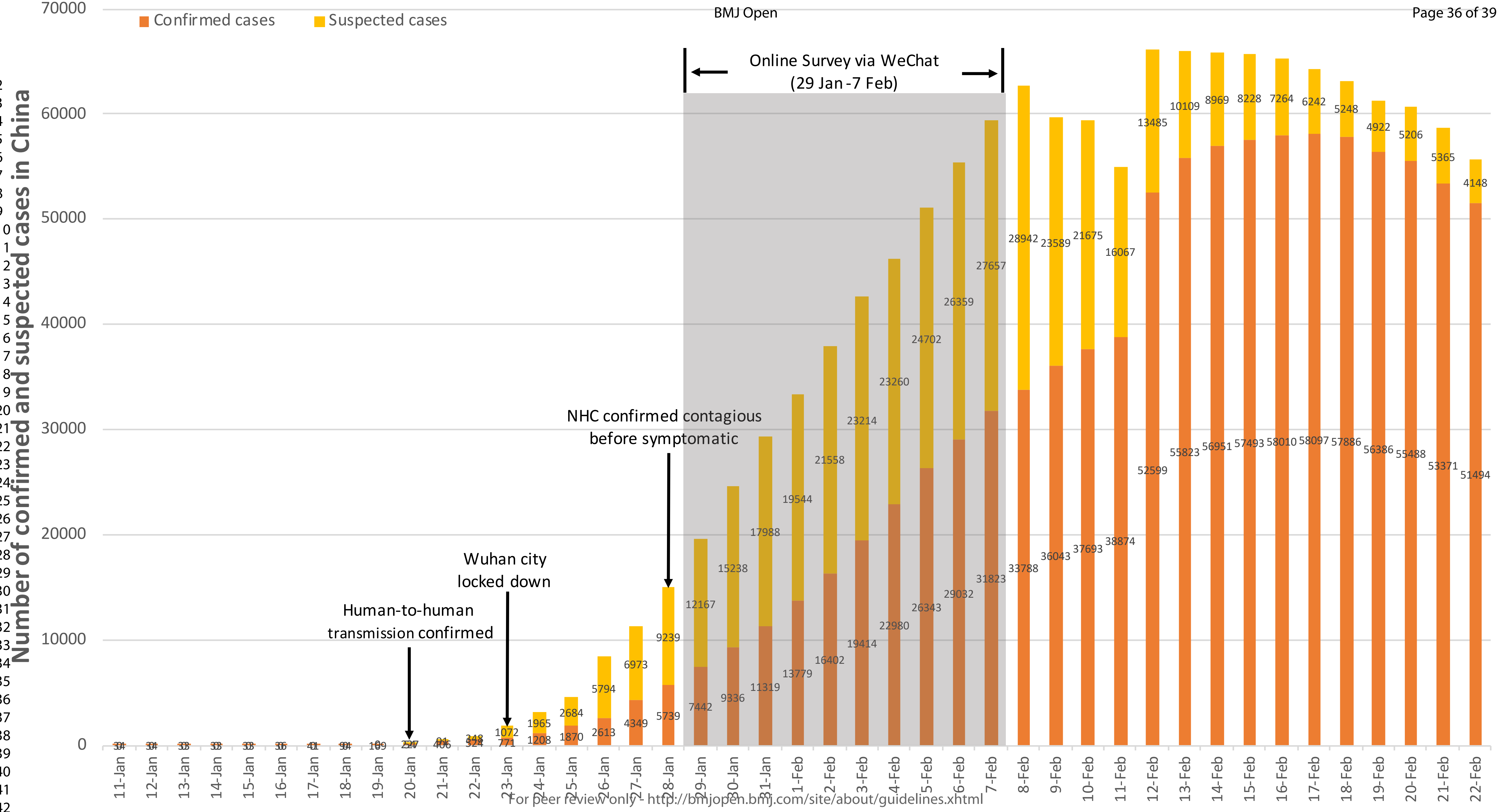
Confirmed cases
Suspected cases
Number of confirmed and suspected cases in China
Online Survey via WeChat
(29 Jan -7 Feb)
NHC confirmed contagious
before symptomatic
Wuhan city
locked down
Human-to-human
transmission confirmed

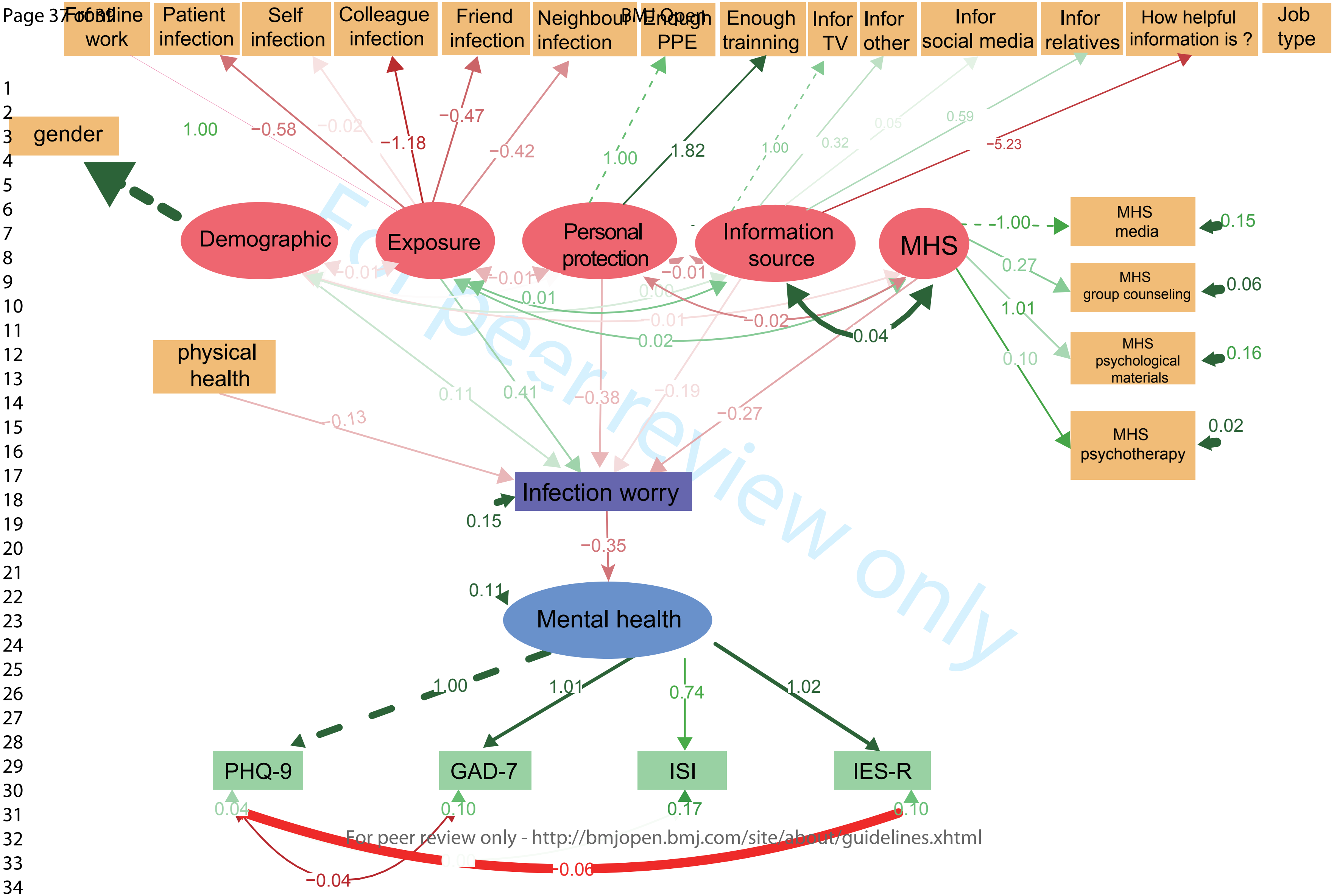

For peer review only

Reporting checklist for cross sectional study.

Based on the STROBE cross sectional guidelines.

Instructions to authors

Complete this checklist by entering the page numbers from your manuscript where readers will find each of the items listed below.

Your article may not currently address all the items on the checklist. Please modify your text to include the missing information. If you are certain that an item does not apply, please write "n/a" and provide a short explanation.

Upload your completed checklist as an extra file when you submit to a journal.

In your methods section, say that you used the STROBE cross sectionalreporting guidelines, and cite them as:

von Elm E, Altman DG, Egger M, Pocock SJ, Gotzsche PC, Vandenbroucke JP. The Strengthening the Reporting of Observational Studies in Epidemiology (STROBE) Statement: guidelines for reporting observational studies.

For peer review only

		Reporting Item	Page Number
**Title and abstract**			
Title	#1a	Indicate the study’s design with a commonly used term in the title or the abstract	1
Abstract	#1b	Provide in the abstract an informative and balanced summary of what was done and what was found	3-5
**Introduction**			
Background / rationale	#2	Explain the scientific background and rationale for the investigation being reported	6
Objectives	#3	State specific objectives, including any prespecified hypotheses	6-7
**Methods**			
Study design	#4	Present key elements of study design early in the paper	7-8
Setting	#5	Describe the setting, locations, and relevant dates, including periods of	7-8

		recruitment, exposure, follow-up, and data collection	
Eligibility criteria	#6a	Give the eligibility criteria, and the sources and methods of selection of participants.	7-8
	#7	Clearly define all outcomes, exposures, predictors, potential confounders, and effect modifiers. Give diagnostic criteria, if applicable	8-11
Data sources / measurement	#8	For each variable of interest give sources of data and details of methods of assessment (measurement). Describe comparability of assessment methods if there is more than one group. Give information separately for for exposed and unexposed groups if applicable.	8-11
Bias	#9	Describe any efforts to address potential sources of bias	13
Study size	#10	Explain how the study size was arrived at	13
Quantitative variables	#11	Explain how quantitative variables were handled in the analyses. If applicable, describe which groupings were chosen, and why	8-11
Statistical methods	#12a	Describe all statistical methods, including those used to control for confounding	11-13
Statistical methods	#12b	Describe any methods used to examine subgroups and interactions	12-13
Statistical methods	#12c	Explain how missing data were addressed	
Statistical methods	#12d	If applicable, describe analytical methods taking account of sampling strategy	
Statistical methods	#12e	Describe any sensitivity analyses	
**Results**			
Participants	#13a	Report numbers of individuals at each stage of study—eg numbers potentially eligible, examined for eligibility, confirmed eligible, included in the study, completing follow-up, and analysed. Give information separately for for exposed and unexposed groups if applicable.	13
Participants	#13b	Give reasons for non-participation at each stage	13
Participants	#13c	Consider use of a flow diagram	

For peer review only

Descriptive data	#14a	Give characteristics of study participants (eg demographic, clinical, social) and information on exposures and potential confounders. Give information separately for exposed and unexposed groups if applicable.	
Descriptive data	#14b	Indicate number of participants with missing data for each variable of interest	14
Outcome data	#15	Report numbers of outcome events or summary measures. Give information separately for exposed and unexposed groups if applicable.	14-15
Main results	#16a	Give unadjusted estimates and, if applicable, confounder-adjusted estimates and their precision (eg, 95% confidence interval). Make clear which confounders were adjusted for and why they were included	15-18
Main results	#16b	Report category boundaries when continuous variables were categorized	15-18
Main results	#16c	If relevant, consider translating estimates of relative risk into absolute risk for a meaningful time period	15-18
Other analyses	#17	Report other analyses done—e.g., analyses of subgroups and interactions, and sensitivity analyses	15-18
**Discussion**			
Key results	#18	Summarise key results with reference to study objectives	19-21
Limitations	#19	Discuss limitations of the study, taking into account sources of potential bias or imprecision. Discuss both direction and magnitude of any potential bias.	21
Interpretation	#20	Give a cautious overall interpretation considering objectives, limitations, multiplicity of analyses, results from similar studies, and other relevant evidence.	19-21
Generalisability	#21	Discuss the generalisability (external validity) of the study results	22
**Other Information**			
Funding	#22	Give the source of funding and the role of the funders for the present study and, if applicable, for the original study on which the present article is based	23

None The STROBE checklist is distributed under the terms of the Creative Commons Attribution License CC-BY. This checklist can be completed online using https://www.goodreports.org/, a tool made by the EQUATOR Network in collaboration with Penelope.ai

For peer review only - http://bmjopen.bmj.com/site/about/guidelines.xhtml

BMJ Open

Acute psychological impact of Coronavirus Disease 2019 outbreak among psychiatric professionals in China：A multicenter, cross-sectional, web-based study

Journal:	*BMJ Open*
Manuscript ID	bmjopen-2020-047828.R3
Article Type:	Original research
Date Submitted by the Author:	28-Apr-2021
Complete List of Authors:	Guo, Xin; Renmin Hospital of Wuhan University,<br>McCutcheon, Robert; King's College London<br>Pillinger, Toby; King's College London<br>Arumuham, Atheeshaan; King's College London<br>Chen, Jianhua ; Shanghai Clinical Research Center for Mental Health, Shanghai Key Laboratory of Psychotic Disorders, Shanghai Mental Health Center, Shanghai Jiao Tong University School of Medicine<br>Ma, Simeng ; Renmin Hospital of Wuhan University<br>Yang, Jun ; Huazhong University of Science and Technology<br>Wang, Ying ; Renmin Hospital of Wuhan University<br>Hu, Shaohua ; Zhejiang University, Department of Psychiatry<br>Wang, Gaohua ; Renmin Hospital of Wuhan University<br>Liu, Zhong-Chun; Renmin Hospital of Wuhan University, Department of Psychiatry
**Primary Subject Heading**:	Mental health
Secondary Subject Heading:	Mental health
Keywords:	COVID-19, MENTAL HEALTH, PSYCHIATRY

SCHOLARONE™
Manuscripts

I, the Submitting Author has the right to grant and does grant on behalf of all authors of the Work (as defined in the below author licence), an exclusive licence and/or a non-exclusive licence for contributions from authors who are: i) UK Crown employees; ii) where BMJ has agreed a CC-BY licence shall apply, and/or iii) in accordance with the terms applicable for US Federal Government officers or employees acting as part of their official duties; on a worldwide, perpetual, irrevocable, royalty-free basis to BMJ Publishing Group Ltd ("BMJ") its licensees and where the relevant Journal is co-owned by BMJ to the co-owners of the Journal, to publish the Work in this journal and any other BMJ products and to exploit all rights, as set out in our licence.

The Submitting Author accepts and understands that any supply made under these terms is made by BMJ to the Submitting Author unless you are acting as an employee on behalf of your employer or a postgraduate student of an affiliated institution which is paying any applicable article publishing charge ("APC") for Open Access articles. Where the Submitting Author wishes to make the Work available on an Open Access basis (and intends to pay the relevant APC), the terms of reuse of such Open Access shall be governed by a Creative Commons licence – details of these licences and which Creative Commons licence will apply to this Work are set out in our licence referred to above.

Other than as permitted in any relevant BMJ Author's Self Archiving Policies, I confirm this Work has not been accepted for publication elsewhere, is not being considered for publication elsewhere and does not duplicate material already published. I confirm all authors consent to publication of this Work and authorise the granting of this licence.

For peer review only

Word count: 3376

Acute psychological impact of Coronavirus Disease 2019 outbreak among psychiatric professionals in China： A multicenter, cross-sectional, web-based study

Xin Guo[1,2], Robert McCutcheon[2,3], Toby Pillinger[2,3], Atheeshaan Arumuham[2,3], Jianhua Chen[4], Simeng Ma[1], Jun Yang[5], Ying Wang[1], Shaohua Hu[6], Gaohua Wang[1], Zhong-Chun Liu[1]

[1]Department of Psychiatry, RenMin Hospital of Wuhan University, Wuhan, China

[2]Department of Psychosis Studies, Institute of Psychiatry, Psychology & Neuroscience, King's college of London, London, UK.

[3]Psychiatric Imaging Group, MRC London Institute of Medical Sciences, Hammersmith Hospital, London, UK

[4]Shanghai Clinical Research Center for Mental Health, Shanghai Key Laboratory of Psychotic Disorders, Shanghai Mental Health Center, Shanghai Jiao Tong University School of Medicine, Shanghai, China.

[5]Computer Science and Technology, Huazhong University of Science and Technology, Wuhan, China.

[6]Department of Psychiatry, First Affiliated Hospital, Zhejiang University School of

For peer review only - http://bmjopen.bmj.com/site/about/guidelines.xhtml

Medicine, Hangzhou, China

Corresponding author:

Zhongchun Liu, PhD

Department of Psychiatry, RenMin Hospital of Wuhan University, Wuhan, China

238 Jiefang Road, Wuchang District, Wuhan, China

Email: zcliu6@whu.edu.cn

For peer review only

For peer review only - http://bmjopen.bmj.com/site/about/guidelines.xhtml

Abstract

Objectives: To assess the magnitude of mental health outcomes and associated factors among psychiatric professionals in mental health services during COVID-19 in China.

Design, Setting and Participants: This cross-sectional, survey-based, region-stratified study collected demographic data and mental health measurements from psychiatric professionals in 34 hospitals from 29 January and 7 February 2020, in China. Hospitals equipped with fever clinics or deployed on wards for patients with COVID-19 were eligible.

Primary Outcome and Measures: The severity of symptoms of depression, anxiety, insomnia, and distress were assessed by the Chinese versions of PHQ-9, GAD-7, ISI and IES-R, respectively. Multivariable logistic regression and Structural Equation Modelling was performed to identify factors associated with mental health outcomes.

Results: A total of 610 psychiatric professionals were included. 29.8% were employed in Wuhan, and 22.5% were frontline workers. A considerable proportion of participants reported symptom of depression (461 [75.6%]), anxiety (282 [46.2%]), insomnia (336 [55.1%]) and mental stress (481 [78.9%]). Psychiatric symptoms were associated with worrying about infection (e.g. OR 2.36[95% CI 1.27-4.39] for anxiety),

For peer review only - http://bmjopen.bmj.com/site/about/guidelines.xhtml

risks of exposure to COVID-19 (e.g. having inadequate PPE, OR 2.43[1.32-4.47] for depression), and self-perceived physical health (e.g. OR 3.22[2.24-4.64] for mental stress). Information sources of COVID-19 were also found to be both positively (e.g. Information from relatives, OR 2.16[1.46-3.21] for mental stress) and negatively (e.g. Information from TV, OR 0.52[0.35-0.77] for mental stress) associated with mental stress. There is preliminary evidence that mental health might benefit from greater availability of mental health care services. The SEM analysis indicated that worrying about infection may be the primary mediator via which risk of exposure to COVID-19 pandemic affects the mental health of psychiatric professionals.

Conclusions: The current findings demonstrate several pathways via which the COVID-19 pandemic may have negatively affected the mental health of psychiatric professionals in China.

For peer review only - http://bmjopen.bmj.com/site/about/guidelines.xhtml

Strengths and limitations of this study

- *Provides a timely evaluation of the acute psychological impact of the COVID-19 pandemic upon the mental health of psychiatric professionals in China*
- *The large scale nature of the study means it is well powered to detect meaningful effects and allow for the control of potentially confounding variables.*
- *A sampling bias exists because of the voluntary response sampling method.*
- *The cross-sectional, observational nature of the study means it is not possible to conclusively determine whether observed associations are causal in nature.*

For peer review only

For peer review only - http://bmjopen.bmj.com/site/about/guidelines.xhtml

Introduction

The 2019 epidemic of novel coronavirus disease (COVID-19) that initiated in Wuhan (Hubei Province, China),[1] is now recognized by the World Health Organization (WHO) as a global public health threat [2]. In addition to the resulting increased burden on physical healthcare services, there is mounting recognition of the impact that the pandemic may have upon public mental health and therefore mental health services. An effectively functioning mental health service requires sufficient levels of staffing, and staff that are in good physical and mental health themselves.

The mental health of health-care workers in general has been affected during the immediate wake of the COVID-19 epidemic.[3] [4] Psychological distress among medical staff has been noted in the form of increased symptoms of anxiety, depression, insomnia and posttraumatic stress [5] [6]. In addition, workers may also experience isolation from their families or community because of infection transmission risk and stigmatization. [7]

The specific impact of the pandemic on the mental health of psychiatric professionals is less well characterised. In the current study, a multicenter survey was performed to assess psychological distress in psychiatric professionals in China one week after Wuhan city entered lock down. A brief questionnaire was designed and distributed to psychiatric professionals of several hospitals through the 'WeChat' group chat

For peer review only - http://bmjopen.bmj.com/site/about/guidelines.xhtml

application, including staff of both psychiatric hospitals and psychiatric staff working in general hospitals and fever clinics. We used the responses from this questionnaire to investigate which factors were associated with an increased risk of mental health symptoms among psychiatric professionals, and whether there was any evidence of potentially protective factors.

2. Methods

2.1 Online survey

A WeChat-based online survey was completed by psychiatric professionals between 29 January and 7 February 2020, the second week after Wuhan city locked down. During the survey period, the cumulative number of confirmed and suspected COVID-19 cases was climbing exponentially (see Fig. 1).

The online survey was conducted with the WeChat-based survey program 'Questionnaire Star' based on a voluntary response sampling method. The target population was psychiatric professionals working in hospitals that established fever clinics or wards for patients with COVID-19. Each WeChat account of a participant was authenticated by their ID card in China. An online advert with a Quick Response (QR) code directing to the online survey was distributed in psychiatric professionals' WeChat working groups of several provinces. Completing the questionnaire outside of working hours was advised. This study was reviewed and approved by the Clinical

For peer review only

Research Ethics Committee of Renmin Hospital of Wuhan University (WDRY2020-K004). All subjects provided informed consent electronically prior to registration. Prior to providing informed consent, potential participants were provided with information regarding the research project including its primary goals. After reading this information potential participants were provided with the option to either decline or consent to take part in the study. Only those who consented were taken to the questionnaire page. Respondents could terminate the survey at any time or during any question if they wished to.

2.2 Patient and Public Involvement

No patients were involved in this study.

2.3 Questionnaire

The questionnaire consists of seven sections: basic demographic data, mental health assessment, risks of exposure to COVID-19, source of information about COVID-19, mental healthcare services (MHS) accessed, worry about infection and self-perceived physical health before the COVID-19 outbreak. The questionnaire required 15 minutes to complete.

2.3.1 Demographic data

Basic demographic data include gender, age, marital status (unmarried/other, married), educational level (high school or lower, undergraduate, postgraduate or

For peer review only - http://bmjopen.bmj.com/site/about/guidelines.xhtml

higher), location, job type (doctor or nurse), job title (junior, intermediate, or senior), and hospital type (tertiary, secondary or other).

2.3.2 Mental health assessment

We used four well validated scales to assess the mental health status of psychiatric professionals. The 9-item Patient Health Questionnaire (PHQ-9), the 7-item Generalized Anxiety Disorder (GAD-7), the 7-item Insomnia Severity Index (ISI) and the 22-item Impact of Event Scale-Revised (IESR) were used to evaluate depression, anxiety, insomnia and stress, respectively. The PHQ-9 is a self-report measure used to assess the severity of depression and has good reliability and validity in Chinese populations (Cronbach's α=0.89). [8] PHQ-9 total scores range from 0-27, and a score of 10 or greater is defined as a high probability of meeting diagnostic criteria for depression. [9]The GAD-7 is a self-rated scale to evaluate the severity of anxiety and has good reliability and validity in Chinese population (Cronbach's α=0.89), [10] the total score of GAD-7 ranges from 0 to 21, and a score of greater than 10 indicates a high probability of meeting diagnostic criteria for anxiety. [11] The ISI is a measure of insomnia severity that has been shown to be valid and reliable, and a total score of greater than 10 is defined as a high probability of meeting diagnostic criteria for insomnia. [12] Post traumatic symptoms were accessed using the IES-R, which also has extensive reliability and validity (Cronbach's α for 3 subscales were 0.89 (Intrusion), 0.85 (Avoidance), and 0.83 (Hyperarousal), respectively), [13] and a score of greater than 34 is identified as significant mental stress. [14]

For peer review only - http://bmjopen.bmj.com/site/about/guidelines.xhtml

2.3.3 Risk of exposure to COVID-19

Exposure to COVID-19 was determined with the following questions asked to psychiatric professionals:

1.Have you been diagnosed with COVID-19?

2. Do you manage patients diagnosed with COVID-19?

3.Has your family been diagnosed with COVID-19?

4.Have your friends been diagnosed?

5.Have your neighbors (people living in the same community who may or may not know each other) been diagnosed?

6. Does anyone living with you have suspected symptoms?

In terms of personal protection, participants were asked whether they had enough personal protection equipment (PPE) and enough training for personal protection. The possible responses to each question were yes or no.

2.3.4 Accessed mental health services (MHS)

The following question was used to determine which psychological services the subject had accessed: “Have you ever received the following services: psychological support materials (leaflets, brochures and books provided by mental health workers and distributed to staff in the hospital), psychological resources available through media (psychological assistance methods and techniques provided by psychologists

For peer review only - http://bmjopen.bmj.com/site/about/guidelines.xhtml

through social media), or online counseling or psychotherapy (including individual or group counselling, or structural psychotherapy)?".

2.3.5 Source of information about COVID-19

The following question was used to determine from which source subjects had accessed information regarding COVID-19: "Which way do you used to get information about COVID-19: relatives (information through direct communication with family, neighbors, colleagues, friends or strangers), social media (information from unofficial accounts in WeChat Moments, Weibo, TikTok, and other digital platform), TV (information from national and local official television channels), and other (other information sources of COVID-19) and daily time (hours) spending on information of COVID-19". Then, participants were asked how helpful the information they received were. Answer options included considerably helpful, medium helpful, slightly helpful or not helpful at all.

2.3.6 Self-perceived physical health

Physical health status was determined by asking participants to compare their health status to normal people before the outbreak of COVID-19: How do you perceive your health status compared to normal people before the outbreak? Answer options included strong, normal or weak.

2.3.7 Worrying about infection

For peer review only

For peer review only - http://bmjopen.bmj.com/site/about/guidelines.xhtml

Worry about infection was determined by asking participants if they worry about their vulnerability to infection of COVID-19 in workplace or at home. Possible responses were yes or no.

2.4 Statistical analysis

All statistical analyses were carried out in the statistical programming language R (version-3.6.0). [15]

A bivariate analysis was performed firstly by using a Chi-square test to determine the effect of six components (demographic data, risks of exposure to COVID-19, source of information about COVID-19, MHS accessed, worry about infection and self-perceived physical health) on prevalence of four mental health symptoms (depression, anxiety, insomnia and mental stress, assessed by using PHQ-9, GAD-7, ISI, and IES-R separately) among psychiatric professionals.

Stepwise logistic regression analysis according to Akaike information criterion (AIC) was then performed to examine the effects of the six components on the four mental health assessment (PHQ-9, GAD-7, ISI, and IES-R scores) separately. The significance of association between the potential risk factors and outcomes were checked by analysis of variance (ANOVA) and prediction accuracy of models were counted by AUC (area under ROC curve).

For peer review only - http://bmjopen.bmj.com/site/about/guidelines.xhtml

Finally, a structural equation model (SEM) was constructed via R package lavaan 0.6-7 [16] to explore the relationship between mental health assessment (PHQ-9. GAD-7, ISI and IES-R) and the six components, namely demographic data, risks of exposure to COVID-19, source of information about COVID-19, MHS accessed, worry about infection and self-perceived physical health. The estimation method used weighted least squares with mean and variance adjustment test statistics.[17] We used a Monte Carlo method with 1000 guided resamplings to construct a confidence interval for the estimation effect.[18] In SEM, several criteria, such as ratio of value of chi-square test of model fit and degree of freedom (x^2 / df) <3, root mean square error of approximation (RMSEA) values<0.08 and goodness-to-fit index (GFI) values>0.90, indicate acceptable models (Hu and Bentler, 1998).

P values < 0.05 indicated that a difference was statistically significant.

3. Results

Initially, 834 completed questionnaires were received, with a response rate of 78.5%. The duration (seconds) to complete the questionnaire was recorded by Questionnaire star in WeChat. We excluded 119 questionnaires with a complete duration of fewer than 5 minutes (300 seconds) or more than 20 minutes (1,200 seconds), to ensure that all questions had been well understood and completed consecutively by respondents.

For peer review only - http://bmjopen.bmj.com/site/about/guidelines.xhtml

We further excluded 105 questionnaires from provinces that had recruited less than 20 participants. In total, 224 questionnaires were excluded, leaving 610 questionnaires for analysis. Respondents completing the included questionnaires and excluded questionnaires were comparable in age and sex.

3.1 Demographic characteristics

In total, 610 psychiatric professionals, including 164 (26.9%) doctors and 446 (73.1%) nurses were included in the analysis. 29.8% of these participants were employed in Wuhan, and 53.3% were employed in Hubei provinces other than Wuhan. 22.5% were frontline mental health staff. 53.3% of participants were working in a tertiary hospital. The participants tended to be female (76.1%), aged 26-40 years (63.1%), married (68.2%), with an educational level of undergraduate (91.3%), and have a junior job title (62.1%), as shown in Table 1.

3.2 Mental health assessment

According to standard questionnaire cut-offs of the PHQ-9, GAD-7, ISI, IES-R scores, 461 (75.6%) of individuals met criteria for depression, 282 (46.2%) for anxiety, 336 (55.1%) for insomnia, and 481 (78.9%) for high levels of mental stress.

3.3 Assessed mental health services (MHS)

36.3% of participants had received psychological support materials, 50.4% had obtained psychological resources available through media, and 17.5% had

For peer review only

For peer review only - http://bmjopen.bmj.com/site/about/guidelines.xhtml

participated in online psychological counseling/psychotherapy, as shown in Table 1.

3.3 Risk factors & protective factors of psychological impact of COVID-19 on psychiatric professionals

Bivariate Analysis

We found that psychiatric professionals who took part in frontline work possessed higher prevalence of all four mental health symptoms (89.9% vs 71.5%, P<0.001 for depression; 66.4% vs 40.4%, P<0.001 for anxiety; 67.9% vs 51.4%, P<0.001 for insomnia; 69.3% vs 52.0%, P<0.001 for mental stress) compared with those who worked in second line services. Treating and diagnosing infected patient (95% vs 72.6%, P<0.001 for depression; 75% vs 41.9%, P<0.001 for anxiety; 75% vs 52.1%, P<0.001 for insomnia; 82.5% vs 51.9%, P<0.001 for mental stress), working with infected colleagues (87.9% vs 71.9%, P<0.001 for depression; 67.9% vs 39.8%, P<0.001 for anxiety; 66% vs 52%, P<0.001 for insomnia; 72.1% vs 51.1%, P<0.001 for mental stress), employed in Wuhan (Wuhan vs Hubei except Wuhan vs Other provinces except Hubei: 89% vs 72.6% vs 61.2%, P<0.001 for depression; 65.4% vs 39.1% vs 35%, P<0.001 for anxiety; 70% vs 49% vs 48.5%, P<0.001 for insomnia; 72.5% vs 49.5% vs 46.6, P<0.001 for mental stress), worse self-perceived physical health (strong vs normal vs weak: 60.7% vs 91.5% vs 100%, P<0.001 for depression; 26.4% vs 67.1% vs 88.9%, P<0.001 for anxiety; 34.6% vs 76.7% vs 100%, P<0.001 for insomnia; 39.6% vs 73.1% vs 88.9, P<0.001 for mental stress), worrying about infection (83.5% vs 46.2%,

For peer review only - http://bmjopen.bmj.com/site/about/guidelines.xhtml

P<0.001 for depression; 54.2% vs 16.9%, P<0.001 for anxiety; 60.6% vs 34.6%, P<0.001 for insomnia; 63.5% vs 27.7%, P<0.001 for mental stress) and obtaining information about COVID-19 from relatives (79.4% vs 71.1%, P=0.02 for depression; 50.9% vs 40.8%, P=0.02 for anxiety; 59% vs 50%, P=0.03 for insomnia; 64.4% vs 46.1%, P<0.001 for mental stress) were also significantly associated with higher prevalence of all four mental health symptoms. Self-infection (100% vs 45.8%, p=0.049) and living with infected neighbor (65.4% vs 39.1, p=0.002) were significantly associated with higher prevalence of anxiety.

Access to mental health services was associated with a lower prevalence of mental health symptoms, both access to psychological support materials (70.7% vs 79.9%, P=0.01 for depression; 38.7% vs 52.9%, P<0.001 for anxiety; 49% vs 61%, P=0.004 for insomnia; 51.6% vs 59.8%, P=0.05 for mental stress), and mental health services accessed through media (71.5% vs 81.3%, P=0.007 for depression; 40.8% vs 54%, P=0.002 for anxiety). Obtaining information about COVID-19 from TV (70.3% vs 81.9%, P=0.001 for depression; 41.7% vs 51.6%, P=0.02 for anxiety; 51% vs 60%, P=0.02 for insomnia; 50.5% vs 62.5%, P=0.004 for mental stress) was also significantly associated with lower prevalence of mental health symptoms, as shown in Table 2.

Logistic Regression

To explore potential risk and protective factors, stepwise logistic regression analyses on PHQ-9, GAD-7, ISI and IES-R scores separately was performed. After controlling

For peer review only - http://bmjopen.bmj.com/site/about/guidelines.xhtml

for potential confounding variables, checking the significance of effect of each variable using ANOVA, we identified risk and protective factors as follows (Table 3):

PHQ-9

Treating and diagnosing infected patient (odds ratio [OR], 2.77; 95% CI, 1.05-7.27; P = .039), worrying about infection (OR, 0.33; 95% CI, 0.21-0.52; p<0.001), having inadequate PPE (OR, 2.43; 95% CI, 1.32-4.47; p=0.005), and worse self-perceived physical health (OR, 2.76; 95% CI, 1.73-4.41; p<0.001) were significantly associated with higher PHQ-9 score among psychiatric professionals. Obtaining information about COVID-19 from relatives (OR, 1.64; 95% CI, 1.03-2.61; p=0.036) was also significantly associated with increased PHQ-9 score, while obtaining information from TV (OR, 0.57; 95% CI, 0.36-0.91; p=0.02) was significantly associated with lower PHQ-9 score.

GAD-7

Doctors were found to have higher GAD-7 scores (OR, 0.55; 95% CI, 0.34-0.91; p=0.019) compared with nurses. Self-infection seems a primary risk factor to anxiety (OR, 2530278.72; 95% CI, 0-Inf; p=0.045), and all self-infection psychiatric professionals met criteria of severe anxiety. Treating and diagnosing infected patient (OR, 2.36; 95% CI, 1.27-4.39; p=0.007), working with infected colleague (OR, 2.17; 95% CI, 1.31-3.61; p=0.003), living with infected neighbor (OR, 2.13; 95% CI, 1.14-3.96; p=0.018), having inadequate PPE (OR, 1.7; 95% CI, 1.09-2.65; p=0.02), worrying

For peer review only - http://bmjopen.bmj.com/site/about/guidelines.xhtml

about infection (OR, 0.25; 95% CI, 0.14-0.44; p<0.001) and obtaining information from other methods (OR, 2.36; 95% CI, 1.29-4.32; p=0.005) were associated with increased GAD-7 score. Mental health services accessed through psychological support materials (OR, 0.66; 95% CI, 0.43-0.99; p<0.001) and those who thought information about COVID-19 was helpful (OR, 1.17; 95% CI, 1.01-1.36; p=0.35) showed lower GAD-7 score.

ISI

Treating and diagnosing infected patients (OR, 1.91; 95% CI, 1.06-3.46; p=0.032), worse physical health status (OR, 5.68; 95% CI, 3.92-8.25; p<0.001) and those worrying about infection (OR, 0.58; 95% CI, 0.36-0.92; p=0.02) were significantly associated with higher ISI score.

IES-R

Treating and diagnosing infected patient (OR, 2.97; 95% CI, 1.54-5.73; p=0.001), worse self-perceived physical health (OR, 3.22; 95% CI, 2.24-4.64; p<0.001) and worrying about infection (OR, 0.35; 95% CI, 0.22-0.56; p<0.001) were significantly associated with higher IES-R score among psychiatric professionals. Psychiatric professionals obtaining information from relatives showed significantly higher IES-R score (OR, 2.16; 95% CI, 1.46-3.21; p<0.001), while those who obtained information from TV (OR, 0.52; 95% CI, 0.35-0.77; p=0.001), and those who thought information

For peer review only - http://bmjopen.bmj.com/site/about/guidelines.xhtml

about COVID-19 was helpful (OR, 0.52; 95% CI, 0.35-0.77; p=0.001) showed lower IES-R score.

Structural Equation Modelling

Finally, we established an SEM of the association between the seven areas described in methods. The chi-square test of model fit yielded a value of 729.6, with degrees of freedom = 253, P value= 0.000, x^2 / df = 2.88, RMSEA = 0.056, GFI = 0.909, and TLI = 0.973, indicating a good fit. The results showed that gender, the risk factors of exposure, source of information about COVID-19 and self-perceived physical health status affected mental health through worry about infection, indicated that worrying about infection could be the primary mediator that affects the mental health of psychiatric professionals during the COVID-19 outbreak. Mental health services alleviated mental health symptoms through decreasing the worry about infection. The results are shown in Figure 2.

4. Discussion

The current study investigated the psychological impact of COVID-19 among psychiatric professionals in China during the first months of the pandemic. The overall prevalence of depression, anxiety, insomnia and stress in psychiatric in the second week after Wuhan city locked down were 75.6%, 46.2%, 55.1%, and 78.9%, respectively.

For peer review only - http://bmjopen.bmj.com/site/about/guidelines.xhtml

In comparison with recent studies of general health care workers, [6, 19, 20] the psychological impact of COVID-19 outbreak seems to be potentially more severe among psychiatric professionals (the overall prevalence of depression, anxiety, insomnia and stress was estimated at 13.9%-46.9%, 16.1%-41.1%, 19.7% and 9.8%-69.1%, respectively). When compared, however, to studies that have focused on similar regions (i.e. Hubei province), the rates appear comparable to other healthcare workers (82.9%-88.7%, 66.9%-80.4%, and 87.6%-92.9%, for depression, anxiety and stress respectively. [19]

We identified several factors that were associated with higher psychiatric symptom burden. An increased risk of exposure to COVID-19, including treating and diagnosing infected patients was significantly associated with higher risk of mental health symptoms among psychiatric professionals. We also found that female healthcare workers had a higher risk of developing depression symptoms but not anxiety, insomnia or mental stress symptoms during the COVID-19 pandemic among psychiatric professionals. A recent meta-analysis study showed that females had a higher risk of developing psychiatric symptoms and reported lower psychological wellbeing during the COVID-19 pandemic, but the findings of the studies included in the published meta-analysis were not consistent.[21] How much of the association is specific to COVID-19, as opposed to a generally increased risk remains to be established.

For peer review only - http://bmjopen.bmj.com/site/about/guidelines.xhtml

This is the first study to explore the psychological effect of different sources of information about COVID-19 on mental health among psychiatric professionals. At the early stage of the outbreak, the unpredictable nature of the pandemic was accompanied by misinformation, often driven by erroneous news reports from unofficial information sources and the public's misunderstanding of health messages, thus causing worry and increasing the risk of mental health problems in population.[3] Our study sugggests that information from social media, direct communication with relatives, and other unofficial information sources is associated with an increased risk of mental health symptoms among psychiatric professionals. Conversely, information obtained from official sources such as TV was associated with reduced symptom severity in psychiatric professionals.

To efficiently cope with psychological impact of the COVID-19 outbreak, the Chinese Government has implemented rapid and comprehensive psychological crisis intervention, including publishing psychological support materials, such as leaflets and books, establishing psychological intervention teams on site, [4] an online mental health service through social media platforms, and also telephone guidance to help address mental health problems.[22] Our study found that the use of some of these interventions (psychological materials and psychological help from media, and online counseling and psychotherapy) was associated with reduced symptom severity.

Our study also has some limitations. First, the study is cross-sectional and does not

For peer review only - http://bmjopen.bmj.com/site/about/guidelines.xhtml

record the mental health status before the outbreak, and can not establish causal associations. There is a potential for a response bias given the voluntary nature of the survey, although at least as regards occupation and gender our sample is not substantially different from the overall workforce. Certain factors may limit the generalizability of our findings. Our study was performed in the early days of the outbreak and was concluded by the end of the second week after Wuhan city locked down. During this period the rapid rise in number of identified and suspected cases and unprecedented scale of the lock down caused public panic and psychological stress, that may have diminished over time. [3]

In summary, the results demonstrate that a strikingly large portion of psychiatric professionals in China suffered from mental health disturbances during the early outbreak of COVID-19. Their mental health was primarily affected by worry about being infected, risks of exposure to COVID-19, self-perceived physical health. Unreliable information sources of COVID-19 may exacerbate mental health symtoms, and healthcare worker mental health may benefit from greater availability of mental health care from public and personalized mental health services.

Acknowledgments

Author Contribution: Author Contribution: Dr Guo, Dr McCutcheon and Prof Liu had full access to all the data in the study and takes responsibility for the integrity of the

For peer review only - http://bmjopen.bmj.com/site/about/guidelines.xhtml

data and the accuracy of the data analysis.

Design and conduct of the study: Dr Guo, Dr McCutcheon, Prof Liu.

Collection, management, analysis, and interpretation of the data: Dr Guo, Dr McCutcheon, Dr Pillinger, Dr Arumuham, Dr Chen, Dr Ma, Dr Yang, Dr Wang, Dr Hu.

Preparation, review, or approval of the manuscript: Dr Guo, Dr McCutcheon, Dr Arumuham, Dr Chen, Prof Liu and Prof Wang.

Decision to submit the manuscript for publication: Prof Liu and Prof Wang.

Funding/Support: XG's work is funded by the National Key R&D Program of China (no. 2018YFC1314600), and fellowship grants from Chinese Scholarship Council. ZCL's work is funded by the National Key R&D Program of China (no. 2018YFC1314600). RM's research is funded by the Wellcome Trust (no. 200102/Z/15/Z) and a NIHR clinical lectureship.

Ethical Approval Statement

This study was reviewed and approved by the Clinical Research Ethics Committee of Renmin Hospital of Wuhan University (WDRY2020-K004).

Data availability statement

For peer review only - http://bmjopen.bmj.com/site/about/guidelines.xhtml

All data relevant to the study are included in the article.

Declaration of interest:

The authors declare that they have no conflict of interest.

References

1 Chan JF, Yuan S, Kok KH, et al. A familial cluster of pneumonia associated with the 2019 novel coronavirus indicating person-to-person transmission: a study of a family cluster. *Lancet* 2020;395(1474-547X (Electronic)):514-23.

2 WHO. Statement on the second meeting of the International Health Regulations (2005) Emergency Committee regarding the outbreak of novel coronavirus (2019-nCoV). https://www.who.int/news-room/detail/30-01-2020-statement-on-thesecond-meeting-of-the-international-health-regulations-(2005)-emergency-committee-regarding-the-outbreak-of-novel-coronavirus-(2019-ncov) (assessed Feb 15, 2020). 2020

3 Bao Y, Sun Y, Meng S, et al. 2019-nCoV epidemic: address mental health care to empower society. *Lancet* 2020;395(1474-547X (Electronic)):e37-e38.

4 Kang L, Li Y, Hu S, et al. The mental health of medical workers in Wuhan, China dealing with the 2019 novel coronavirus. *Lancet Psychiatry* 2020(2215-0374 (Electronic)) doi: https://doi.org/10.1016/s2215-0366(20)30047-x

5 da Silva FCT, Neto MLR. Psychiatric symptomatology associated with depression, anxiety, distress, and insomnia in health professionals working in patients affected by COVID-19: A systematic review with meta-analysis. *Prog Neuropsychopharmacol Biol Psychiatry* 2020(1878-4216 (Electronic)) doi: 10.1016/j.pnpbp.2020.110057

6 Lin K, Yang BX, Luo D, et al. The Mental Health Effects of COVID-19 on Health Care Providers in China. *American Journal of Psyhciatry* 2020(1535-7228 (Electronic))

7 Van Bortel T, Basnayake A, Wurie F, et al. Psychosocial effects of an Ebola outbreak at individual, community and international levels. *Bull World Health Organ* 2016;94(1564-0604 (Electronic)):210-14.

8 Chen S, Fang Y, Chiu H, et al. Validation of the nine-item Patient Health Questionnaire to screen for major depression in a Chinese primary care population. *Asia Pac Psychiatry* 2013;5(1758-5872 (Electronic)):61-68.

9 Manea L, Gilbody S, McMillan D. Optimal cut-off score for diagnosing depression with the Patient Health Questionnaire (PHQ-9): a meta-analysis. *Canadian Medical Association Journal* 2012;184(1488-2329 (Electronic)):e191-e96.

10 Tong X, An D, McGonigal A, et al. Validation of the Generalized Anxiety Disorder-7 (GAD-7) among Chinese people with epilepsy. *Epilepsy Res* 2015(1872-6844 (Electronic))

For peer review only

For peer review only - http://bmjopen.bmj.com/site/about/guidelines.xhtml

11 Spitzer R, Kroenke K, Williams J, et al. A brief measure for assessing generalized anxiety disorder: the GAD-7. *Arch Intern Med* 2006;166(0003-9926 (Print)):1092-97.
12 Morin C, Belleville G, Bélanger L, et al. The Insomnia Severity Index: psychometric indicators to detect insomnia cases and evaluate treatment response. *Sleep* 2011;34(1550-9109 (Electronic)):601-08.
13 Wu K, Chan K. The development of the Chinese version of Impact of Event Scale--Revised (CIES-R). *Soc Psychiatry Psychiatr Epidemiol* 2003;38(0933-7954 (Print)):94-98.
14 Morina N, Ehring T, Priebe S. Diagnostic utility of the impact of event scale-revised in two samples of survivors of war. *PLoS One* 2013;31(1932-6203 (Electronic)):e83916.
15 Team RC. R: A Language and Environment for Statistical Computing. 2013
16 Rosseel Y. Lavaan: A R package for structural equation modeling. *Journal of Statistical Software* 2012;48(2):1-36.
17 DiStefano C, Morgan GB. A Comparison of Diagonal Weighted Least Squares Robust Estimation Techniques for Ordinal Data. *Structural Equation Modeling: A Multidisciplinary Journal* 2014;21(3):425-38. doi: 10.1080/10705511.2014.915373
18 Bauer DJ, Preacher KJ, Gil KM. Conceptualizing and testing random indirect effects and moderated mediation in multilevel models: new procedures and recommendations. *Psychol Methods* 2006;11(2):142-63. doi: 10.1037/1082-989x.11.2.142 [published Online First: 2006/06/21]
19 Wang Y, Ma S, Yang C, et al. Acute psychological effects of Coronavirus Disease 2019 outbreak among healthcare workers in China: a cross-sectional study. *Transl Psychiatry* 2020;10(2158-3188 (Electronic)):348.
20 Wang L, Zhang M, Liu G, et al. Psychological impact of coronavirus disease (2019) (COVID-19) epidemic on medical staff in different posts in China: A multicenter study. *Journal of Psychiatric Research* 2020;129(1879-1379 (Electronic)):198-205.
21 Cinelli M, Quattrociocchi W, Galeazzi A, et al. The COVID-19 social media infodemic. *Sci Rep* 2020(2045-2322 (Electronic)) doi: 10.1038/s41598-020-73510-5
22 Liu S, Yang L, Zhang C, et al. Online mental health services in China during the COVID-19 outbreak. *Lancet Psychiatry* 2020(2215-0374 (Electronic)) doi: 10.1016/S2215-0366(20)30077-8

For peer review only - http://bmjopen.bmj.com/site/about/guidelines.xhtml

Figure & Table Legend

Figure 1. Timeline of COVID-19 and survey data. Wuhan city was locked down since 23 Jan 2020, and the online survey was conducted 1 week after that. The cumulative number seemed to have entered the rapid rise phase.

------------------------------- Figure 1-----------------------------

Figure 2. Direct and indirect effects in SEM. The solid line represents a significant relationship between the two, while the dotted line represents the relationship is not significant.

------------------------------- Figure 2----------------------------

For peer review only - http://bmjopen.bmj.com/site/about/guidelines.xhtml

For peer review only

Table 1. Demographic characteristics and accessed mental health services

Variables		Number	Percentage (%)
Total		610	100
Gender	Female	464	76.1
	Male	146	23.9
Age (years)	18-25	92	15.1
	26-30	198	32.5
	31-40	187	30.7
	>40	133	21.8
Marital status	Unmarried/Other	194	31.8
	Married	416	68.2
Education level	High school or less	18	3.0
	Undergraduate	557	91.3
	Graduate or higher	35	5.7
Location	Wuhan	182	29.8
	Hubei except Wuhan	325	53.3
	Guangdong	50	8.2
	Heibei	25	4.1
	Yunan	28	4.6
Job type	Doctor	164	26.9
	Nurse	446	73.1
Job title	Junior	379	62.1
	Intermediate	158	25.9

For peer review only

	Senior	73	12.0
Hospital type	tertiary	325	53.3
	Secondary or other	285	46.7
Working position	Front line	137	22.5
	Secondary line	473	77.5
Assessed mental health services			
psychological support materials	Yes	287	47.0
	No	323	53.0
MHS available through media	Yes	358	58.7
	No	252	41.3
counseling or psychotherapy	Yes	82	13.4
	No	528	86.6

Table 2. Prevalence of acute psychological effects among psychiatric professionals

Variables	Total	PHQ-9			GAD-7			ISI			IES-R		
		No	Yes	p-value	No	Yes	p-value	No	Yes	p-value	No	Yes	p-value
Total	610												
Gender				0.002			0.09			0.26			0.08
Male	146	50(34.2)	96(65.8)		88(60.3)	58(39.7)		72(49.3)	74(50.7)		74(50.7)	72(49.3)	
Female	464	99(21.3)	365(78.7)		240(51.7)	224(48.3)		202(43.5)	262(56.5)		195(42.0)	269(58.0)	
Physical health				<0.001			<0.001			<0.001			<0.001
Strong	318	125(39.3)	193(60.7)		234(73.6)	84(26.4)		208(65.4)	110(34.6)		192(60.4)	126(39.6)	
Normal	283	24(8.5)	259(91.5)		93(32.9)	190(67.1)		66(23.3)	217(76.7)		76(26.9)	207(73.1)	

For peer review only - http://bmjopen.bmj.com/site/about/guidelines.xhtml

Weak	9	0(0)	9(100)		1(11.1)	8(88.9)		0(0)	9(100)		1(11.1)	8(88.9)	
Worrying about infection				<0.001			<0.001			<0.001			<0.001
Yes	480	79(16.5)	401(83.5)		220(45.8)	260(54.2)		189(39.4)	291(60.6)		175(36.5)	305(63.5)	
No	130	70(53.8)	60(46.2)		108(83.1)	22(16.9)		85(65.4)	45(34.6)		94(72.3)	36(27.7)	
Work position				<0.001			<0.001			<0.001			<0.001
Frontline	137	14(10.2)	123(89.8)		46(33.6)	91(66.4)		44(32.1)	93(67.9)		42(30.7)	95(69.3)	
Secondary line	473	135(28.5)	338(71.5)		282(59.6)	191(40.4)		230(48.6)	243(51.4)		227(48)	246(52)	
Self-infection				0.45			0.049			0.12			0.12
No	605	149(24.6)	456(75.4)		328(54.2)	277(45.8)		274(45.3)	331(54.7)		269(44.5)	336(55.5)	
Yes	5	0(0)	5(100)		0(0)	5(100)		0(0)	5(100)		0(0)	5(100)	
Patient infection				<0.001			<0.001			<0.001			<0.001
No	530	145(27.4)	385(72.6)		308(58.1)	222(41.9)		254(47.9)	276(52.1)		255(48.1)	275(51.9)	
Yes	80	4(5)	76(95)		20(25)	60(75)		20(25)	60(75)		14(17.5)	66(82.5)	
Family member infection				0.28			0.84			0.21			0.65
No	603	149(24.7)	454(75.3)		325(53.9)	278(46.1)		273(45.3)	330(54.7)		267(44.3)	336(55.7)	
Yes	7	0(0)	7(100)		3(42.9)	4(57.1)		1(14.3)	6(85.7)		2(28.6)	5(71.4)	
Colleague infection				<0.001			<0.001			0.003			<0.001
No	470	132(28.1)	338(71.9)		283(60.2)	187(39.8)		227(48)	243(52)		230(48.9)	240(51.1)	
Yes	140	17(12.1)	123(87.9)		45(32.1)	95(67.9)		47(34)	93(66)		39(27.9)	101(72.1)	
Friend infection				0.44			0.14			0.18			0.22
No	571	142(24.9)	429(75.1)		312(54.6)	259(45.4)		261(46)	310(54)		256(44.8)	315(55.2)	
Yes	39	7(17.9)	32(82.1)		16(41)	23(59)		13(33)	26(67)		13(33)	26(67)	
Neighbour infection				0.13			0.002			0.61			0.27
No	533	136(25.5)	397(74.5)		300(56.3)	233(43.7)		242(45)	291(55)		240(45)	293(55)	

For peer review only - http://bmjopen.bmj.com/site/about/guidelines.xhtml

Yes	77	13(16.8)	64(83.1)		28(36.4)	49(63.6)		32(42)	45(58)		29(37.7)	48(62.3)	
Location				<0.001			<0.001			<0.001			<0.001
Wuhan	182	20(11)	162(89)		63(34.6)	119(65.4)		54(30)	128(70)		50(27.5)	132(72.5)	
Hubei except Wuhan	325	89(27.4)	236(72.6)		198(60.9)	127(39.1)		167(51)	158(49)		164(50.5)	161(49.5)	
Other provinces except Hubei	103	40(38.8)	63(61.2)		67(65)	36(35)		53(51.5)	50(48.5)		55(53.4)	48(46.6)	
MHS psychological materials				0.01			<0.001			0.004			0.05
No	323	65(20.1)	258(79.9)		152(47.1)	171(52.9)		127(39)	196(61)		130(40.2)	193(59.8)	
Yes	287	84(29.3)	203(70.7)		176(61.3)	111(38.7)		147(51)	140(49)		139(48.4)	148(51.6)	
MHS available through media				0.007			0.002			0.11			0.15
No	252	47(18.7)	205(81.3)		116(46)	136(54)		103(41)	149(59)		102(40.5)	150(59.5)	
Yes	358	102(28.5)	256(71.5)		212(59.2)	146(40.8)		171(48)	187(52)		167(46.6)	191(53.4)	
Information from relatives				0.02			0.02			0.03			<0.001
No	284	82(28.9)	202(71.1)		168(59.2)	116(40.8)		141(50)	143(50)		153(53.9)	131(46.1)	
Yes	326	67(20.6)	259(79.4)		160(49.1)	166(50.9)		133(41)	193(59)		116(35.6)	210(64.4)	
Information from TV				0.001			0.02			0.02			0.004
No	277	50(18.1)	227(81.9)		134(48.4)	143(51.6)		110(40)	167(60)		104(37.5)	173(62.5)	
Yes	333	99(29.7)	234(70.3)		194(58.3)	139(41.7)		164(49)	169(51)		165(49.5)	168(50.5)	

Table 3. Risk & protective factors of mental health of psychiatric professionals identified by stepwise logistic regression

Variables	crude OR (95%CI)	crude P value	adj. OR (95%CI)	P (Wald's test)	P(LR-test)
**PHQ-9**					
gender: male vs female	0.6 (0.39,0.92)	0.019	0.63 (0.38,1.03)	0.063	0.066

For peer review only - http://bmjopen.bmj.com/site/about/guidelines.xhtml

Physical health (cont. var.)	3.9 (2.51,6.06)	< 0.001	2.76 (1.73,4.41)	< 0.001	< 0.001
Patient infection: yes vs no	4.58 (1.81,11.58)	0.001	2.77 (1.05,7.27)	0.039	0.022
MHS media: yes vs no	0.65 (0.43,0.98)	0.039	0.69 (0.43,1.09)	0.112	0.11
MHS psychotherapy: no vs yes	3.84 (0.5,29.46)	0.196	4.05 (0.49,33.61)	0.195	0.127
Having enough personal protection (PP): no vs yes	3.15 (1.78,5.59)	< 0.001	2.43 (1.32,4.47)	0.005	0.003
Worrying about infection: no vs yes	0.21 (0.13,0.32)	< 0.001	0.33 (0.21,0.52)	< 0.001	< 0.001
Information from relatives: yes vs no	1.81 (1.22,2.68)	0.003	1.64 (1.03,2.61)	0.036	0.035
Information from TV: yes vs no	0.63 (0.42,0.93)	0.022	0.57 (0.36,0.91)	0.02	0.018
**GAD-7**					
gender: male vs female	0.71 (0.48,1.03)	0.071	0.62 (0.37,1.03)	0.066	0.064
Physical health (cont. var.)	5.62 (3.99,7.92)	< 0.001	4.96 (3.37,7.29)	< 0.001	< 0.001
Job type: doctor vs nurse	0.84 (0.59,1.2)	0.343	0.55 (0.34,0.91)	0.019	0.018
Self-infection: yes vs no	2508169.79 (0,Inf)	0.97	2530278.72 (0,Inf)	0.979	0.045
Patient infection: yes vs no	4.16 (2.44,7.1)	< 0.001	2.36 (1.27,4.39)	0.007	0.006
Colleague infection: yes vs no	3.19 (2.14,4.77)	< 0.001	2.17 (1.31,3.61)	0.003	0.002
Friend infection: yes vs no	1.73 (0.9,3.35)	0.102	0.48 (0.2,1.11)	0.086	0.087
Neighbour infection: yes vs no	2.25 (1.37,3.7)	0.001	2.13 (1.14,3.96)	0.018	0.016
MHS psychological support material: yes vs no	0.56 (0.41,0.77)	< 0.001	0.66 (0.43,0.99)	0.045	0.045
Having enough personal protection (PP): no vs yes	2.22 (1.53,3.23)	< 0.001	1.7 (1.09,2.65)	0.02	0.02
Worryinng about infection: no vs yes	0.17 (0.11,0.28)	< 0.001	0.25 (0.14,0.44)	< 0.001	< 0.001
Information from TV: yes vs no	0.67 (0.49,0.93)	0.015	0.73 (0.49,1.1)	0.131	0.131
Information from other methods: yes vs no	1.46 (0.91,2.35)	0.119	2.36 (1.29,4.32)	0.005	0.005
Thinking Information helpful? (cont. var.)	1.33 (1.18,1.49)	< 0.001	1.17 (1.01,1.36)	0.035	0.035
**ISI**					

For peer review only - http://bmjopen.bmj.com/site/about/guidelines.xhtml

gender: male vs female	0.79 (0.55,1.15)	0.221	0.97 (0.62,1.5)	0.88	0.88
education (cont. var.)	1.44 (0.82,2.5)	0.201	1.76 (0.95,3.27)	0.073	0.07
Physical health (cont. var.)	6.35 (4.46,9.06)	< 0.001	5.68 (3.92,8.25)	< 0.001	< 0.001
Patient infection: yes vs no	2.76 (1.62,4.71)	< 0.001	1.91 (1.06,3.46)	0.032	0.027
MHS psychological support material: yes vs no	0.62 (0.45,0.85)	0.003	0.74 (0.51,1.07)	0.111	0.111
MHS psychotherapy: no vs yes	1.65 (0.56,4.89)	0.366	2.63 (0.8,8.63)	0.112	0.103
Having enough training: no vs yes	1.95 (1.37,2.79)	< 0.001	1.45 (0.95,2.21)	0.088	0.087
Having enough personal protection (PP): no vs yes	1.99 (1.36,2.91)	< 0.001	1.4 (0.9,2.2)	0.135	0.134
Worrying about infection: no vs yes	0.34 (0.23,0.52)	< 0.001	0.58 (0.36,0.92)	0.020	0.019
Information from social media: yes vs no	2.08 (0.75,5.81)	0.16	2.76 (0.81,9.4)	0.103	0.093
Information from TV: yes vs no	0.68 (0.49,0.94)	0.019	0.7 (0.49,1.02)	0.067	0.066
**IES-R**					
education (cont. var.)	1.54 (0.88,2.7)	0.129	1.58 (0.84,2.97)	0.154	0.15
gender: male vs female	0.71 (0.49,1.02)	0.067	0.81 (0.52,1.25)	0.34	0.341
Physical health (cont. var.)	4.1 (2.93,5.74)	< 0.001	3.22 (2.24,4.64)	< 0.001	< 0.001
Patient infection: yes vs no	4.37 (2.4,7.98)	< 0.001	2.97 (1.54,5.73)	0.001	< 0.001
Having enough training: no vs yes	1.92 (1.35,2.75)	< 0.001	1.42 (0.95,2.14)	0.089	0.089
Worrying about infection: no vs yes	0.22 (0.14,0.34)	< 0.001	0.35 (0.22,0.56)	< 0.001	< 0.001
Information from relatives: yes vs no	2.11 (1.53,2.93)	< 0.001	2.16 (1.46,3.21)	< 0.001	< 0.001
Information from TV: yes vs no	0.61 (0.44,0.85)	0.003	0.52 (0.35,0.77)	0.001	< 0.001
Think information helpful? (cont. var.)	1.24 (1.1,1.39)	< 0.001	1.15 (1,1.31)	0.045	0.044

For peer review only

For peer review only - http://bmjopen.bmj.com/site/about/guidelines.xhtml

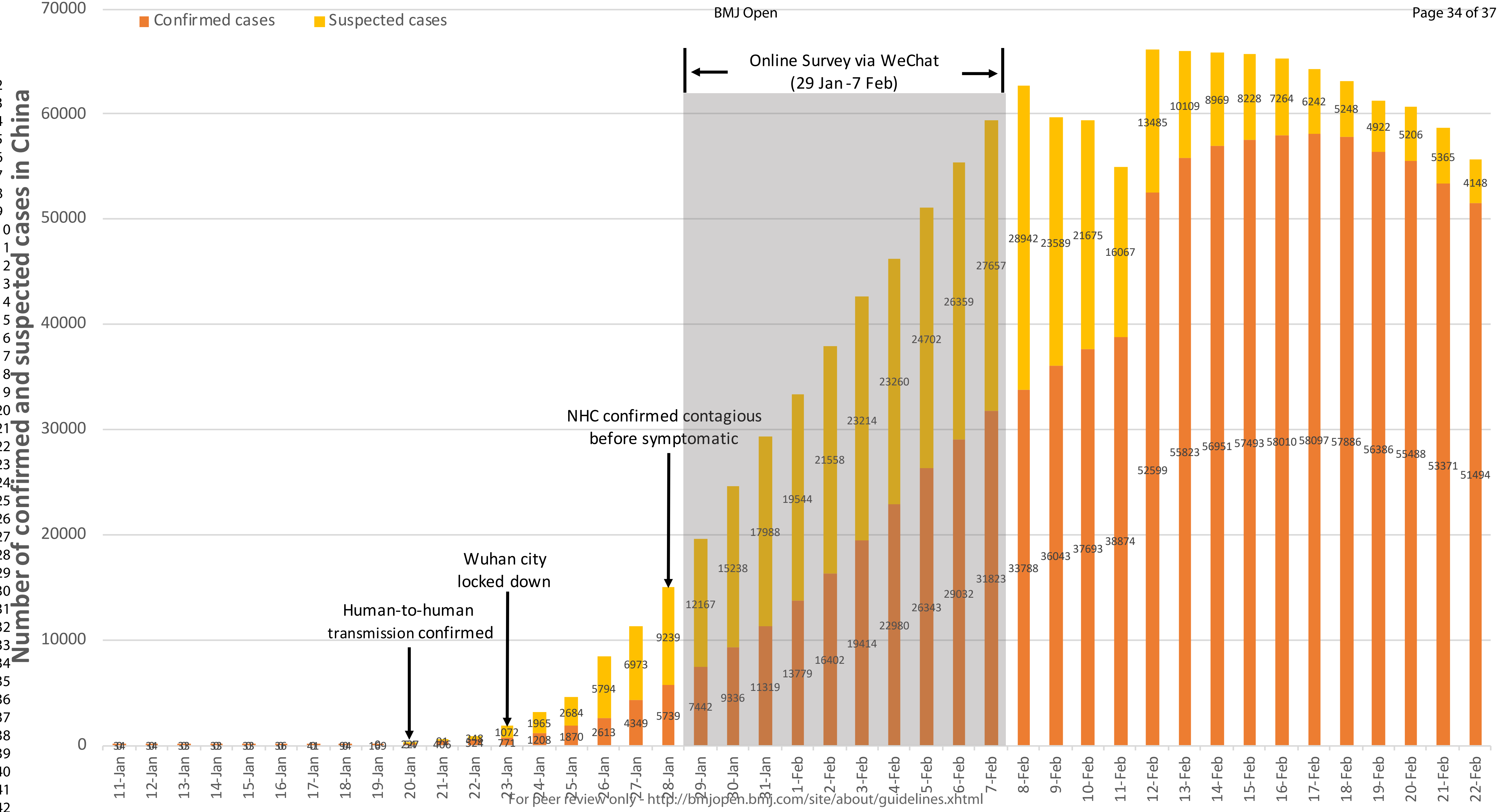
Confirmed cases
Suspected cases
Number of confirmed and suspected cases in China
70000
60000
50000
40000
30000
20000
10000
0
Online Survey via WeChat
(29 Jan -7 Feb)
NHC confirmed contagious
before symptomatic
Wuhan city
locked down
Human-to-human
transmission confirmed
11-Jan
12-Jan
13-Jan
14-Jan
15-Jan
16-Jan
17-Jan
18-Jan
19-Jan
20-Jan
21-Jan
22-Jan
23-Jan
24-Jan
25-Jan
26-Jan
27-Jan
28-Jan
29-Jan
30-Jan
31-Jan
1-Feb
2-Feb
3-Feb
4-Feb
5-Feb
6-Feb
7-Feb
8-Feb
9-Feb
10-Feb
11-Feb
12-Feb
13-Feb
14-Feb
15-Feb
16-Feb
17-Feb
18-Feb
19-Feb
20-Feb
21-Feb
22-Feb

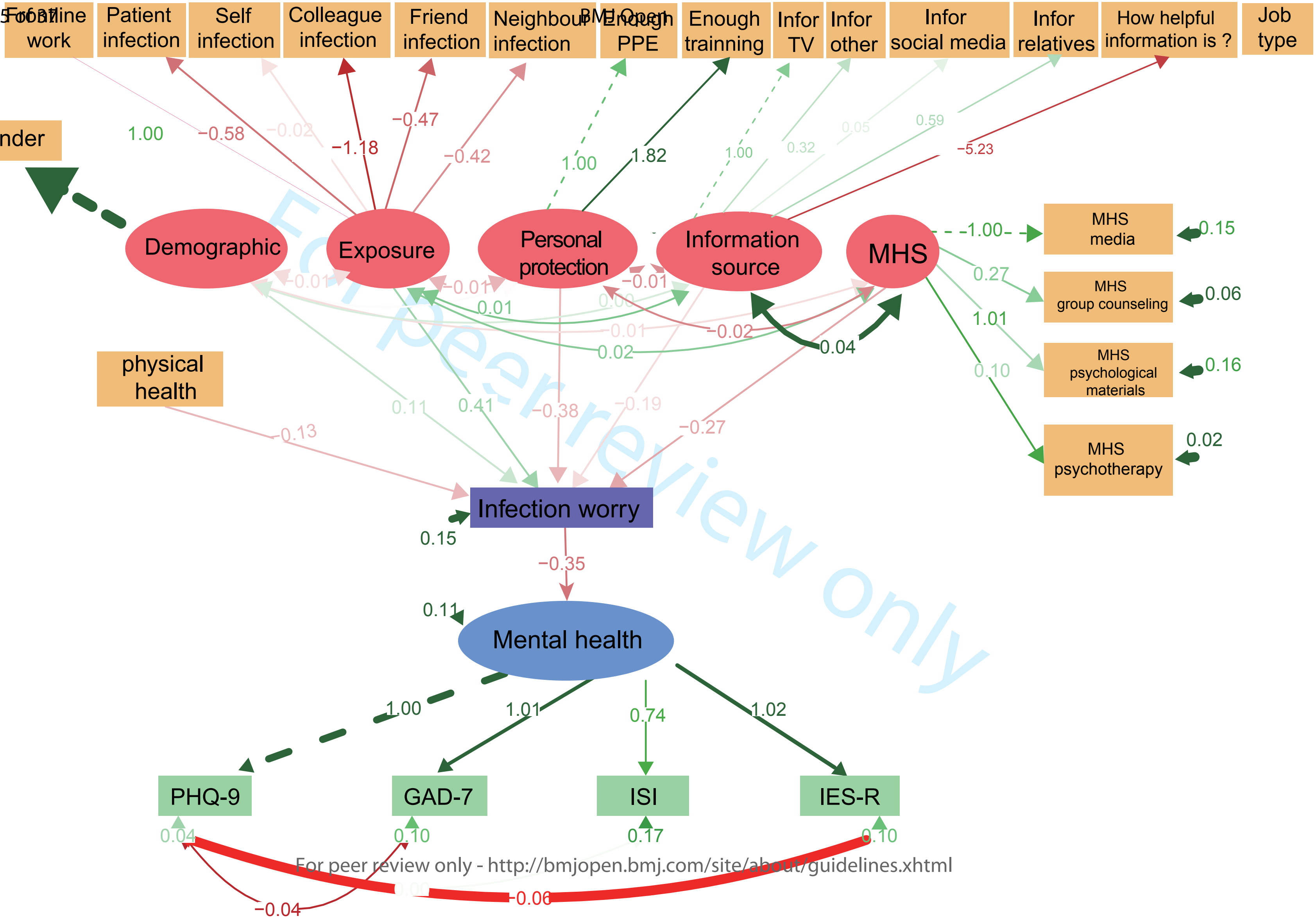

For peer review only - http://bmjopen.bmj.com/site/about/guidelines.xhtml

Reporting checklist for cross sectional study.

Based on the STROBE cross sectional guidelines.

Instructions to authors

Complete this checklist by entering the page numbers from your manuscript where readers will find each of the items listed below.

Your article may not currently address all the items on the checklist. Please modify your text to include the missing information. If you are certain that an item does not apply, please write "n/a" and provide a short explanation.

Upload your completed checklist as an extra file when you submit to a journal.

In your methods section, say that you used the STROBE cross sectionalreporting guidelines, and cite them as:

von Elm E, Altman DG, Egger M, Pocock SJ, Gotzsche PC, Vandenbroucke JP. The Strengthening the Reporting of Observational Studies in Epidemiology (STROBE) Statement: guidelines for reporting observational studies.

		Reporting Item	Page Number
**Title and abstract**			
Title	#1a	Indicate the study's design with a commonly used term in the title or the abstract	1
Abstract	#1b	Provide in the abstract an informative and balanced summary of what was done and what was found	3-5
**Introduction**			
Background / rationale	#2	Explain the scientific background and rationale for the investigation being reported	6
Objectives	#3	State specific objectives, including any prespecified hypotheses	6-7
**Methods**			
Study design	#4	Present key elements of study design early in the paper	7-8
Setting	#5	Describe the setting, locations, and relevant dates, including periods of	7-8

For peer review only

		recruitment, exposure, follow-up, and data collection	
Eligibility criteria	#6a	Give the eligibility criteria, and the sources and methods of selection of participants.	7-8
	#7	Clearly define all outcomes, exposures, predictors, potential confounders, and effect modifiers. Give diagnostic criteria, if applicable	8-11
Data sources / measurement	#8	For each variable of interest give sources of data and details of methods of assessment (measurement). Describe comparability of assessment methods if there is more than one group. Give information separately for for exposed and unexposed groups if applicable.	8-11
Bias	#9	Describe any efforts to address potential sources of bias	13
Study size	#10	Explain how the study size was arrived at	13
Quantitative variables	#11	Explain how quantitative variables were handled in the analyses. If applicable, describe which groupings were chosen, and why	8-11
Statistical methods	#12a	Describe all statistical methods, including those used to control for confounding	11-13
Statistical methods	#12b	Describe any methods used to examine subgroups and interactions	12-13
Statistical methods	#12c	Explain how missing data were addressed	
Statistical methods	#12d	If applicable, describe analytical methods taking account of sampling strategy	
Statistical methods	#12e	Describe any sensitivity analyses	
**Results**			
Participants	#13a	Report numbers of individuals at each stage of study—eg numbers potentially eligible, examined for eligibility, confirmed eligible, included in the study, completing follow-up, and analysed. Give information separately for for exposed and unexposed groups if applicable.	13
Participants	#13b	Give reasons for non-participation at each stage	13
Participants	#13c	Consider use of a flow diagram	

For peer review only

Descriptive data	#14a	Give characteristics of study participants (eg demographic, clinical, social) and information on exposures and potential confounders. Give information separately for exposed and unexposed groups if applicable.	
Descriptive data	#14b	Indicate number of participants with missing data for each variable of interest	14
Outcome data	#15	Report numbers of outcome events or summary measures. Give information separately for exposed and unexposed groups if applicable.	14-15
Main results	#16a	Give unadjusted estimates and, if applicable, confounder-adjusted estimates and their precision (eg, 95% confidence interval). Make clear which confounders were adjusted for and why they were included	15-18
Main results	#16b	Report category boundaries when continuous variables were categorized	15-18
Main results	#16c	If relevant, consider translating estimates of relative risk into absolute risk for a meaningful time period	15-18
Other analyses	#17	Report other analyses done—e.g., analyses of subgroups and interactions, and sensitivity analyses	15-18
**Discussion**			
Key results	#18	Summarise key results with reference to study objectives	19-21
Limitations	#19	Discuss limitations of the study, taking into account sources of potential bias or imprecision. Discuss both direction and magnitude of any potential bias.	21
Interpretation	#20	Give a cautious overall interpretation considering objectives, limitations, multiplicity of analyses, results from similar studies, and other relevant evidence.	19-21
Generalisability	#21	Discuss the generalisability (external validity) of the study results	22
**Other Information**			
Funding	#22	Give the source of funding and the role of the funders for the present study and, if applicable, for the original study on which the present article is based	23

None The STROBE checklist is distributed under the terms of the Creative Commons Attribution License CC-BY. This checklist can be completed online using https://www.goodreports.org/, a tool made by the EQUATOR Network in collaboration with Penelope.ai

For peer review only
